# Supplementary material for: Urinary detection of therapy-induced senescence and fibrosis using an injectable albumin-based nanoprobe
Source: Nat Aging. 2026 May 13;6(5):1158–76. doi: 10.1038/s43587-026-01116-z (PMC13190281; doi:10.1038/s43587-026-01116-z)
Supplement: Supplementary file 1 — Supplementary Methods, Supplementary Figures 1–34, Supplementary Uncropped Immunoblots, and Supplementary Tables 1–6. [file 43587_2026_1116_MOESM1_ESM.pdf]

nature aging

Supplementary information

https://doi.org/10.1038/s43587-026-01116-z

Urinary detection of therapy-induced senescence and fibrosis using an injectable albumin-based nanoprobe

In the format provided by the authors and unedited

Supplementary Material For:

Urinary Detection of Senescence and Fibrosis Using Injectable Albumin-based Nanoprobe

Muhamad Hartono[1,2], Jianfeng Ge[2], Mary Denholm[2,5], Matthew G. Ellis[3], Joaquín Araos Henríquez[2], Andrew G. Baker[1], Robert C. Rintoul[2,4], Tijmen Euser[3], Ljiljana Fruk[1*], Daniel Muñoz-Espín[2*]

[1]Department of Chemical Engineering and Biotechnology, University of Cambridge, Cambridge CB3 0AS, UK.

[2]Early Cancer Institute, Department of Oncology, University of Cambridge, Cambridge CB2 0XZ, UK.

[3]Nanophotonics Centre, Department of Physics, Cavendish Laboratory, University of Cambridge, Cambridge CB3 0HE, UK.

[4]Royal Papworth Hospital NHS Foundation Trust, Cambridge Biomedical Campus, Cambridge CB2 0AY, UK.

[5]Department of Oncology, Addenbrooke's Hospital, Cambridge CB2 0QQ, UK.

*Corresponding authors: lf389@cam.ac.uk and dm742@cam.ac.uk

Contents

Supplementary Methods 4

Supplementary Fig. 1. Chemotherapeutic drugs induced senescence in A549, L1475 and HPF-a cells. 9

Supplementary Fig. 2. Uncropped western blot for senescence validation and intracellular MMP-7 level. 10

Supplementary Fig. 3. Senescent human and murine cancer cells abundantly secrete MMP-7 into the conditioned media, while fibroblast cells do not. 12

Supplementary Fig. 4. Kinetics of MMP-7 secretion during senescence. 13

Supplementary Fig. 5. Drug-treated mice showed slower tumor growth, partially due to senescence induction. 14

Supplementary Fig. 6. Higher p21$^+$ levels in tumors correlated positively with higher levels of circulating MMP-7 in the serum of drug-treated mice. 15

Supplementary Fig. 7. Cisplatin and pemetrexed-treated mice had a significantly higher increase in MMP-7 level in their serum following treatment. 16

Supplementary Fig. 8. MMP-7 in the CM of senescent A549 cells was enzymatically active. 17

Supplementary Fig. 9. Cisplatin treatment induces tumor senescence in orthotopically transplanted lung cancer mouse models, accompanied by a higher MMP-7 level. 18

Supplementary Fig. 10. Validation of senescence in other cancer cell lines 19

Supplementary Fig. 11. Different human cancer cells secrete distinct proteases when undergoing senescence. 20

Supplementary Fig. 12. Histological images of lungs, heart, and kidney obtained from 2-month-old vs 19-month-old C57BL/6 mice. 21

Supplementary Fig. 13. Histological images of lungs, heart, and kidney obtained from non-tumour-bearing, cisplatin- vs vehicle-treated C57BL/6 mice 22

Supplementary Fig. 14. Preparation and characterization of AuNC-azide. 23

Supplementary Fig. 15. AuNC-azide with varying peptide content. 24

Supplementary Fig. 16. Elemental analysis of AuNC-azide 25

Supplementary Fig. 17. Molar mass determination of AuNC-azide. 26

Supplementary Fig. 18. Functionalizing albumin with DBCO groups. 27

Supplementary Fig. 19. MALDI spectra of DBCO-functionalized albumin. 28

Supplementary Fig. 20. Characterization of ALBANC nanoprobe. 29

Supplementary Fig. 21. Preparation of nanoprobe using AuNC with different peptide amount. 30

Supplementary Fig. 22. Optimization of AuNC loading in ALBANC nanoprobe. 31

Supplementary Fig. 23. Stability of AuNC and ALBANC nanoprobe *in vitro*. 32

Supplementary Fig. 24. Assembly of non-cleavable, control nanoprobe. 33

Supplementary Fig. 25. Preparation of AF750-labelled ALBANC nanoprobe. 34

Supplementary Fig. 26. Optimization of peroxidase assay for AuNC detection. 36

Supplementary Fig. 27. Sensitivity and kinetics of AuNC detection using peroxidase assay. 37

Supplementary Fig. 28. Exploring the ability of AuNC to form metallic nanoparticles. 38

Supplementary Fig. 29. Characterization of AuNC-Ag alloy formation assay. 39

Supplementary Fig. 30. GSH is important for AuNC-Ag alloy to form. 40

Supplementary Fig. 31. Optimization of alloy formation assay using silver nitrate. 41

Supplementary Fig. 32. Nanoprobes could be cleaved by recombinant MMP-7. 42

Supplementary Fig. 33. Representative images of untreated and cisplatin-treated wild-type (WT) and MMP-7$^{-/-}$ A549 cells fixed and stained for SA-β-gal activity. 43

Supplementary Fig. 34. Nanoprobes did not show any toxic effects *in vitro* and *in vivo*. 44
Supplementary Fig. 35. Renal clearance, biodistribution, and pharmacokinetics of AuNC and nanoprobes. 45
Supplementary Fig. 36. Colorimetric signals from free AuNC versus nanoprobe. 46
Supplementary Fig. 37. Colorimetric assays enabled sensitive detection of renally cleared AuNC in the urine. 47
Supplementary Fig. 38. Platinum-treated tumor specimens from non-small cell lung (NSCLC) patients showed a high expression of senescence markers and MMP-7. 48
Supplementary Fig. 39. Platinum-treated tumor specimens showed a higher expression of MMP-7 and p21 compared to treatment-naive tumor specimens. 50
Supplementary Fig. 40. Peroxidase assay enabled spectroscopic, urinary detection of chemotherapy-induced senescence in lung cancer. 52
Supplementary Fig. 41. Urinary signal correlates positively with circulating MMP-7 level in the serum. 53
Supplementary Fig. 42. Histological validation of pulmonary fibrosis in bleomycin-treated mice. 54
Supplementary Fig. 43. Serum concentration of MMP-7 in untreated mice vs mice after bleomycin treatment (10 days) 56
Supplementary Fig. 44. The levels of fibrotic marker (Masson’s) and MMP-7 correlated positively with the levels of senescence marker in the lungs of fibrotic mice 57
Supplementary Fig. 45. Peroxidase assay enabled spectroscopic, urinary detection of pulmonary fibrosis. 58
Supplementary Fig. 46. Urinary signals correlated positively with MMP-7 levels in the lungs and serum of fibrotic mice. 59
Supplementary Fig. 47. Peroxidase assay for early/incipient fibrosis experiment. 60
Supplementary Fig. 48. Uncropped polyacrylamide gel electrophoresis (PAGE) blots. 61
Supplementary Table 1. Patient data. 62
Supplementary Table 2. List of primary antibodies used for immunohistochemistry**.** 62
Supplementary Table 3. Parameters used for RT-qPCR. 62
Supplementary Table 4. Sequences of oligonucleotide primers employed in the amplification of target genes during RT-qPCR. 62
Supplementary Table 5. List of antibodies used for western blot. 62

Supplementary Materials and Methods

Materials

Gold (III) chloride trihydrate (HAuCl4, 254169), L-glutathione reduced (GSH, G4251), hydrogen peroxide (H_2O_2, H1009), L-cysteine methyl ester hydrochloride (410209), calcium chloride anhydrous ($CaCl_2$, 1.02378), dimethyl sulfoxide (DMSO, 276855), Brij-35 (8.01962) were purchased from Sigma-Aldrich or Merck and used as received. HEPES (17ppn-737E) was purchased from BioWhittaker. Silver nitrate ($AgNO_3$, CHE3244) and ethylenediamine tetra acetic acid (EDTA, 10339513) were purchased from Scientific Laboratories. L-Ascorbic acid sodium salt (A17759.22) was purchased from Thermo Fisher and cetyl trimethyl ammonium chloride (CTAC) (10762402) was purchased from Fisher Scientific. Copper (II) sulphate pentahydrate (CuSO4, 23174.460) was purchased from VWR. Sodium bicarbonate (195497) was purchased from MP Biomedicals. Azidoacetyl-KGRPLALWRSGGGC peptide was procured from Peptide Synthetics whereas Azidoacetyl-KGGGGGGGC peptide was procured from Bioserv UK.

Senescence-associated β-galactosidase (SA-β-gal) staining

Senescent and non-senescent cells were plated on a 6-well plate (300,000 cells/well) and allowed to adhere for 24 h. Cells were then thoroughly rinsed twice with pre-warmed PBS, then underwent fixation and staining to examine senescence associated β-galactosidase (SA-β-gal) activity using the Senescence β-Galactosidase Staining Kit (Cell Signaling, 23833) in accordance with the manufacturer's guidelines. Stained cells were imaged using an Olympus Compact Brightfield Modular Microscope (Life Technologies) at 10X and 20X magnification and analyzed using ZEN Blue software (v2.6). Similarly, xenograft tumors that were removed from mice were stained entirely for SA-β-Gal, utilizing a similar methodology and kit, except the incubation with X-gal was done at 37°C for 4 h.

Serum extraction from blood samples

Blood samples were collected into tubes with anticoagulants (EDTA-treated). By spinning the tubes in a refrigerated centrifuge (4°C) for 10 minutes at 2,500 *x g*, cells and debris were separated from the serum as pellets. The serum was transferred into a new tube. An extended spin of 10 minutes at 16,200 *x g* further removes platelets from the serum. The clear fluid that remains on top after centrifugation, the serum, was transferred into a new microcentrifuge tube using a pipette. Samples were kept at a temperature range of 2-8°C during processing. Serum samples were aliquoted and stored at -80°C until further use or analysis.

Proliferation assay

Senescent (3500 cells/well) and non-senescent (1500 cells/well) were plated in a 96-well plate (Corning) and allowed to adhere for 24 h. Following this, 4 pictures per well were taken at 10X magnification every 2 h for 72 h with an IncuCyte S3 Live Cell Analysis System microscope (Essen Bioscience). Cell confluence was analyzed for each time point using the IncuCyte ZOOM^{TM} software (Essen Bioscience). The confluence over time was normalized to the initial confluence at t = 0 h.

Reverse transcription quantitative polymerase chain reaction (RT-qPCR)

RNA was isolated from cells using the Monarch® Total RNA Miniprep Kit (New England Biolabs, T2010S) and was then resuspended in RNase-free water. To analyse gene expression, complementary DNA (cDNA) was synthesized using the High-Capacity RNA-to-cDNA^{TM} Kit (Thermo Fisher Scientific, 4368814), with a total of 500 ng of RNA per reaction. Reverse transcription quantitative polymerase chain reaction (RT-qPCR) was conducted using 1 µl of the cDNA solution and 500 nM of each primer per well, combined with the Luna®

Universal qPCR Master Mix (New England Biolabs, M3003X), as per the manufacturer's guidelines. For the amplification process, a QuantStudio® 1 Real-Time PCR instrument (Applied Biosystems) was employed with the amplification parameters as specified in Supplementary Table 3. The primers to amplify the target genes were pre-designed KiCqStart® SYBR® Green Primers (Sigma), as enumerated in Supplementary Table 4. Finally, log2 fold change of genes relative to the reference (β-actin) was quantified using the established ΔΔCT method.

Western blot

Protein extraction was carried out using Radioimmunoprecipitation Assay buffer (RIPA, Sigma Aldrich, R0278), supplemented with 1 mM EDTA, cOmplete^{TM} EDTA-free EASYpak protease inhibitor cocktail (Roche, 04693132001) and PhosSTOP^{TM} EASYpak phosphatase inhibitor cocktail (Roche, 4906837001). The lysates underwent a 15-minute incubation on ice and were then centrifuged at 16,000 *x g* for another 15 minutes. The protein levels in the supernatant were measured using the Pierce^{TM} BCA Protein Assay Kit (Thermo Fisher Scientific). For each sample, 30 µg of protein was mixed with Laemmli Sample Buffer (Bio-Rad) and electrophoresed for 1 h at 120V on a Mini-PROTEAN® TGX Precast Gel. The proteins were then transferred from the gel to a PDVF membrane using a wet tank transfer process, which was carried out for 2 h at room temperature (100 V). The membrane was first activated in methanol for 30 s and subsequently washed with Tris Buffered Saline with 1% Tween 20 (TBS-T buffer) and blocked using a 5% milk solution overnight. After 1 h incubation with primary antibodies at room temperature, the membranes were washed three times (10 mins/wash) with TBS-T buffer and then incubated with HRP-conjugated secondary antibodies for 1 h at room temperature. Lastly, after three rounds of washing with TBS-T, the membranes were treated with Enhanced Chemiluminescence Detection Solution (ECL, Amersham) and imaged using a ChemiDoc Imager (Bio-Rad) with Image Lab^{TM} software (v6.1). A list of antibodies used for western blotting is available in Supplementary Table 5.

Generation of conditioned media

Conditioned media (CM) from senescent and non-senescent cells were generated as follows. Senescent cells were plated at 70-80% confluence on a 10 cm dish, whereas non-senescent cells were plated at 40-50% confluence in a complete medium. The following day, the adhered cells were rinsed twice with pre-warmed PBS (10 ml) to ensure no proteases from the serum used for culturing the cells were left. Then, as much as 8 ml of fresh serum-free DMEM was added to the dish and left to condition for 24 hours. After this period, the CM was gathered and transferred to Falcon tubes before being centrifuged for 10 minutes at 102 *x g* at 4°C. The supernatant was then carefully transferred to new Falcon tubes and underwent another centrifugation for 10 minutes at 1125 *x g* at 4°C to remove any cell debris. This conditioned medium was immediately stored at -80°C until it was used for analysis.

Human protease array and human protease inhibitors array

The CM derived from senescent and non-senescent cells were analyzed for the relative levels of secreted proteases present using the Proteome Profiler Human Protease Array Kit (R&D systems, ARY021B) and Proteome Profiler Human Protease Inhibitor Array Kit (R&D systems, ARY023), as per the instructions provided by the manufacturer. A total of 1000 µl of CM was combined with 500 µl of blocking buffer supplied by the kit. The membranes were imaged with a ChemiDoc imager (Bio-Rad) at different exposure times (from 30 seconds to 2 minutes). Following the subtraction of background noise, pixel density was quantified using the Image-J Software. Since the number of proteins secreted by the cells depends on the number of cells, the measured levels of secreted proteases need to be adjusted by cell numbers, which was set to be between 150,000 to 200,000 cells.

MTS cytotoxicity studies

In vitro cytotoxicity of nanoprobe and AuNC was assessed by MTS assay. A549 cells (3000 cells/well), senescent A549 cells (5000 cells/well), and HPF-a cells (5000 cells/well) were plated in a 96-well plate and incubated to adhere to the wells. Twenty-four hours after seeding, the cells were treated with different concentrations of nanoprobe and AuNC (in PBS) for 72 h. Controls include untreated cells (negative control) and cells treated with 0.2% Triton-X (positive control). Cell viability was measured using the MTS (3-(4,5-dimethylthiazol-2-yl)5-(3-carboxymethoxyphenyl)-2-(4-sulfophenyl)-2H-tetrazolium) assay (Promega, G3582) following manufacturer's instructions.

Tissue homogenization

Tumor samples (100 mg) were homogenized in ice-cold RIPA buffer (Roche) using the TissueLyser (Qiagen). All homogenized samples were then centrifuged twice at 16,200 *x g* for 15 min in a refrigerated centrifuge at 4°C to remove any cell debris as pellets. The supernatants were collected at stored at -80°C until further analysis.

Human and murine MMP-7 enzyme-linked immunoassay (ELISA)

The concentration of human MMP-7 in the CM derived from control and senescent cells, as well as other samples (tissue homogenates and blood samples), was quantified using the Human Total MMP-7 Quantikine enzyme-linked immunoassay (ELISA) Kit (R&D systems, DMP700). The assay was subsequently carried out as described in the kit, with each sample tested in technical duplicates. Absorbance at 450 nm was measured using an Infinite 200 PRO Plate Reader (Tecan Life Sciences), after applying a wavelength correction set to 540 nm. A dilution factor of 1:1.5 was used. A similar protocol was carried out to measure the concentration of murine MMP-7 using Mouse MMP-7 ELISA Kit (Antibodies, A314109) following manufacturer's instructions.

Histology

Samples for histological examination were carefully collected, preserved, dried, and prepared for paraffin embedding and slicing. Cryo-sectioning was performed, and the resulting slides were then stored at -80°C until used for SA-β-gal staining. Meanwhile, immunohistochemistry (IHC) staining was conducted on the 5-7 μm paraffin sections. The tissue sections were positioned on Superfrost®plus slides and allowed to dry overnight. IHC was facilitated through an automatic immunostaining platform (Autostainer Link by Dako and Bond by Leica). Antigen retrieval was the initial step and was performed using Tris-EDTA buffer at pH 9, followed by the blockage of innate peroxidase using 3% H_2O_2. The slides were subsequently subjected to the relevant primary antibodies. At post-primary antibody application, the slides were treated with matching secondary antibodies and necessary visualization systems (Bond Polymer Refine Detection, Bond, Leica; EnVision FLEX+, Dako), each of which was conjugated with horseradish peroxidase. The immunohistochemical reaction was enabled using 3,3'-diaminobenzidine tetrahydrochloride (DAB) obtained from Dako. The final stages involved dehydration, clarification, and application of a permanent mounting medium to prepare the slides for a microscopic study. Finally, the complete slides were scanned with an AxioScan Z1 from Zeiss, and images were captured with the Zen Blue Software (Zeiss). Supplementary Table 1 provides a list of the antibodies used and the methods employed for antigen retrieval. All procedures were performed at the Early Cancer Institute, University of Cambridge.

MMP-7 activity assay

MMP-7 activities in serum samples were determined using SensoLyte 520 MMP-7 assay kit (Anaspec, AS-71153) as per manufacturer's protocol. A dilution factor of 1:2 was used.

ICP-MS

AuNC samples, including urine samples from *in vivo* experiments, were first digested using aqua regia (Trace Metal Grade hydrochloric acid, Fisher Chemical and nitric acid, VWR) for 18 h. These samples were then diluted further into a matrix (4% hydrochloric acid and 4% nitric acid). The quantification of gold content in samples was conducted using an iCAP7400 Duo ICP spectrometer (Thermo Fisher Scientific).

Estimation of elemental content in AuNC

The molecular weight of the AuNCs was determined using liquid chromatography-mass spectrometry (LC-MS). The content of ligands (GSH and peptides) attached to the AuNCs was estimated by measuring the percentage of sulfur (S) and gold (Au) using inductively coupled plasma mass spectrometry (ICP-MS) analysis. To quantify the number of biotinylated ligands on each AuNC, the biotin concentration in the filtrate after purification was measured and subtracted from the initial concentration of the biotinylated peptide used. The amount of biotin in the filtrate was determined using the Pierce Biotin Quantitration Kit (28005), following the manufacturer's protocol (Thermo Fisher). Since the sulfur content from ICP reflects the total amount of GSH and peptides attached, the number of GSH molecules could then be estimated.

Nanoprobe cleavage using recombinant proteases

For cleavage kinetics, nanoprobe (0.116 mM [AuNC]) was incubated with recombinant MMP-7 (Merck, 444270, 20 mg/mL, 104.5 µM, approximately 3000 Units/mg protein) for a final enzyme concentration of 50 nM in 50 mM HEPES, pH 7.5, 10 mM $CaCl_2$ and 0.05% Brij-35. The mixtures (20 µl) contained about 500 molar excesses of AuNC to MMP-7. The reaction mixtures were incubated at 37°C with gentle shaking (450 rpm) for 1 – 1440 min. The cleaved AuNC was separated by centrifugation (Amicon, 30 kDa Sigma, 3195 *x g*, 10 min) where it remained in the supernatant. The liberated AuNC was measured using peroxidase assays. For cleavage sensitivity studies, similar experiments were performed, while keeping the nanoprobe concentration and incubation time (3 h) constant with varying recombinant MMP-7 concentrations (0.001 - 1674 µM). For cleavage specificity studies, similar experiments were performed by incubating the cleavable and non-cleavable nanoprobes with different proteases (50 µM), namely human MMP-1 (Eurogentec, AS-72008), MMP-2 (Abcam, ab81550), MMP-3 (Abcam, ab96555), MMP-8, MMP-9 (Sigma Aldrich, PF024- 5UG), MMP-12 (Enzo Life Sciences, BML-SE138-0010), CTSB (Abcam, ab283434), CTSD (Abcam, ab307480).

Polyacrylamide gel electrophoresis (PAGE)

Polyacrylamide gel electrophoresis (PAGE) for AuNC, nanoprobe, and for cleavage study was performed as follows. Samples (16 µl, with varying weight from 1 to 10 µg) were mixed with 4 µl Pierce*TM* lithium dodecyl sulfate (LDS) Sample Buffer, Non-Reducing (4X) (Thermo Fisher, 84788). Samples were loaded onto 4–20% Mini-PROTEAN® TGX*TM* Pre-cast Protein Gels (Biorad, 4561094) and run at 70 V for 50-60 min using NuPAGE*TM* MES SDS Running Buffer (Thermo Fisher, NP0002). Following which, gels were washed with deionized water and stained with Quick Coomassie Stain (Proteinark, GEN-QC-STAIN-1L) for 3-4 h at RT and/or SuperSignal*TM* West Pico PLUS Chemiluminescent Substrate (Thermo Fisher, 34580) for 15 min. Prior to imaging, gels were washed with deionized water twice. Gels were then imaged using ChemiDoc Imaging System (Bio-rad).

Preparation of Alexa Fluor 750 (AF750)-labelled ALBANC nanoprobe

AuNC was reacted with Alexa Fluor 750 (AF750) NHS ester (Thermo Fisher, A20011) *via* amide coupling reactions. AuNC (0.552 mM, 500 µl) was first buffer exchanged into sodium carbonate 0.1 M, pH 8.3 using ultracentrifugation (10 kDa, 3195 *x g*, 15 min). Then, AuNC (500 µl) was mixed with AF750 (10 mg/ml, 100 µl) for 2 h at RT under shaking at 500 rpm. The resulting AF750-functionalized AuNC (AuNC-AF750) was purified using

ultracentrifugation (10 kDa, 3195 *x g*, 15 min) by washing with PBS containing 10% DMSO (five times) followed by washing with PBS (three times). To prepare AF750-labelled nanoprobe, a protocol similar to the preparation of ALBANC nanoprobe was performed involving AuNC-AF750.

MMP-7$^{-/-}$ A549 knockout experiment

Plasmids psPAX2 and pMD2.G were gifts from Didier Trono (Addgene # 12260 and # 12259), lenti cas9-blast was a gift from Feng Zhang (Addgene# 52962). pKLV2-U6gRNA5(BbsI)-PGKpuro2ABFP-W (Addgene#67974) was a gift from Kosuke Yusa. The sgRNA sequence used to knock-out MMP-7 was AATCGATCCACTGTAATATG (Thermo Fisher). MMP7 sgx and sgy was cloned into pKLV2-U6gRNA5(BbsI)-PGKpuro2ABFP-W plasmid. HEK293T cells were transfected with a mixture of the lentiviral transfer plasmid containing genes of interest, psPAX2 and pMD2.G using PEI reagent (MW 25,000, Alfa Aesar). The supernatant containing the lentivirus was collected 72 hours post-transfection and filtered by a 0.45μM PVDF sterile filter (ELKAY). Cells were transduced with the lentiviral supernatant in the presence of 5 μg/mL polybrene (Millipore). Puromycin (4 μg/mL) was used for infected cells selection which started 48 hours post-transduction.

Young-old mice experiments

Female C57BL/6 mice, either 2 months or 19 months old were used. Lungs, kidney, and heart were collected and processed for histological analysis.

Healthy tissues with cisplatin mice experiments

C57BL/6 mice (12.3-week-old) were injected either with saline or with cisplatin (0.1 ml/ml) twice, one week apart, culled 1 week after final injections. At the endpoint, major organs were collected and processed for histological analysis.

L1475(luc) **orthotopic mice experiments**

For orthotopic transplantation of lung cancer cells, female C57BL/6J mice were sublethally irradiated at 4 Gy using a Caesium source irradiator 6 h prior cell injection. Luciferase-expressing Kras$^{G12D/WT}$ murine lung tumour L1475(luc) cells were injected intravenously (2x105 cells/mouse). Baseline luminescence was recorded 24 h after transplantation, and tumour growth was monitored twice a week by bioluminescence imaging following intraperitoneal injection with D-luciferin (150 mg/kg body weight, Perkin Elmer) using an IVIS Spectrum Xenogen imaging system (Caliper Life Sciences) and Living Image software (version 4.7.3).

Re-analysis of single-cell RNA sequencing datasets

Processed and annotated single-cell RNA sequencing data from Huang et al (PMID: 39729352) was downloaded from https://figshare.com/articles/dataset/scRNA-sequencing_raw_data_of_LUAD/24797265 as stated from the original publication. A Seurat object compiling all detected cells by Huang et al (PMID: 39729352) was created from the available count and meta data matrices using the CreateSeuratObject function of the Seurat package (v5.3). The epithelial cell cluster was obtained from the complete dataset using the subset function within Seurat (v5.3). Data from epithelial cells were re-processed using the standard Seurat pipeline and integrated using the Canonical Correlation Analysis (CCA) method. Uniform manifold approximation and projection (UMAP) was used to visualise the dataset in a two-dimensional space. Differential expression testing was performed using the FindMarkers function selecting "MAST" as test type. Significant upregulated genes between lung adenocarcinoma patients treated with neoadjuvant chemotherapy (NCT) (n=5) and naïve lung adenocarcinoma patients (Control, n=4) were defined by showing $Log_2Fold \geq 1$ and significant adjusted p-value or false discovery rate (FDR) <0.05. Gene set enrichment analysis was performed using the clusterProfiler R package (v4.14.6) against the Hallmark, the C2

canonical pathway collection (C2.cp.v5.1) and Human SenMayo (PMID: 35974106) gene sets that were downloaded from the Molecular Signatures Database (https://www.gsea-msigdb.org/gsea/msigdb).

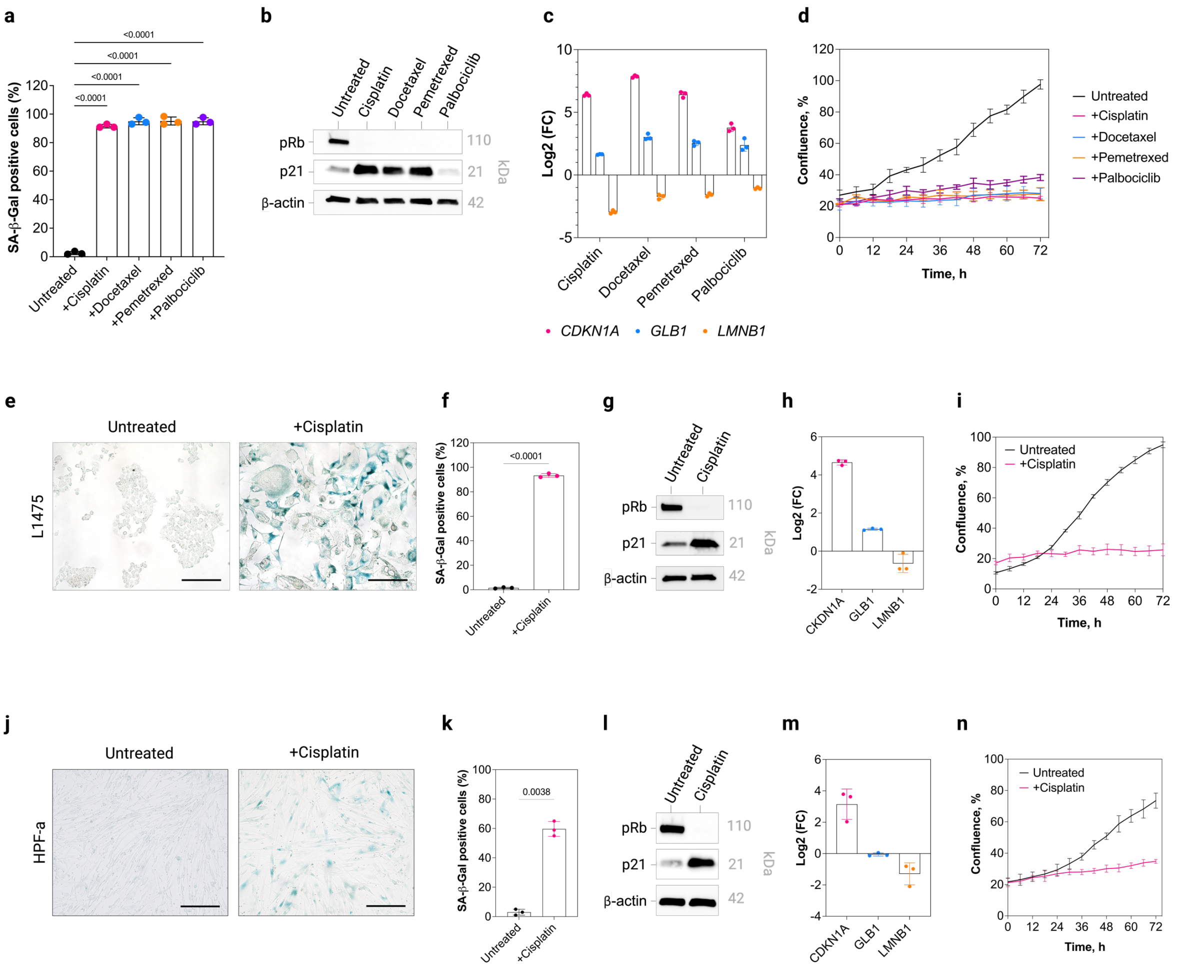

Supplementary Fig. 1. **Chemotherapeutic drugs induced senescence in A549, L1475 and HPF-a cells. a.** The percentage of SA-β-Gal$^+$ A549 cells from each treatment group, corresponding to **Fig. 2b**. **b**. Western blot for the expression of relevant senescence markers in untreated and drug-treated A549 cells. **c**. Log2 fold change (FC) gene expression levels of different senescence markers in senescent A549 cells relative to untreated (non-senescent) cells. **d**. Growth curve (confluence vs time) of untreated and drug treated A549 cells (N = 3, mean ± s.d.; one-way ANOVA with Dunnett's multiple comparisons test). **e.** Representative images of untreated and cisplatin-treated L1475 cells fixed and stained for SA-β-gal activity. Scale bar = 100 µm. **f**. The corresponding percentage of SA-β-gal$^+$ L1475 cells from each treatment group (N = 3, mean ± s.d.; t-test). **g**. Western blot for the expression of relevant senescence markers in untreated and treated L1475 cells. **h**. Log2 FC gene expression levels of different senescence markers in cisplatin treated L1475 cells relative to untreated cells. **i.** Growth curve (confluence vs time) of control and senescent L1475 cells (N = 3, mean ± s.d.). **j**. Representative images of untreated and cisplatin-treated HPF-a cells fixed and stained for SA-β-Gal activity. Scale bar = 100 µm. **k**. The corresponding percentage of SA-β-gal$^+$ HPF-a cells from each treatment group (N = 3, mean ± s.d.; t-test). **l**. Western blot for the expression of relevant senescence markers in untreated and treated HPF-a cells. **m**. Log2 FC gene expression levels of different senescence markers in cisplatin-treated HPF-a cells relative to untreated cells. **n**. Growth curve (confluence vs time) of untreated and cisplatin-treated HPF-a cells (N = 3, mean ± s.d.). pRb: hyperphosphorylated Retinoblastoma.

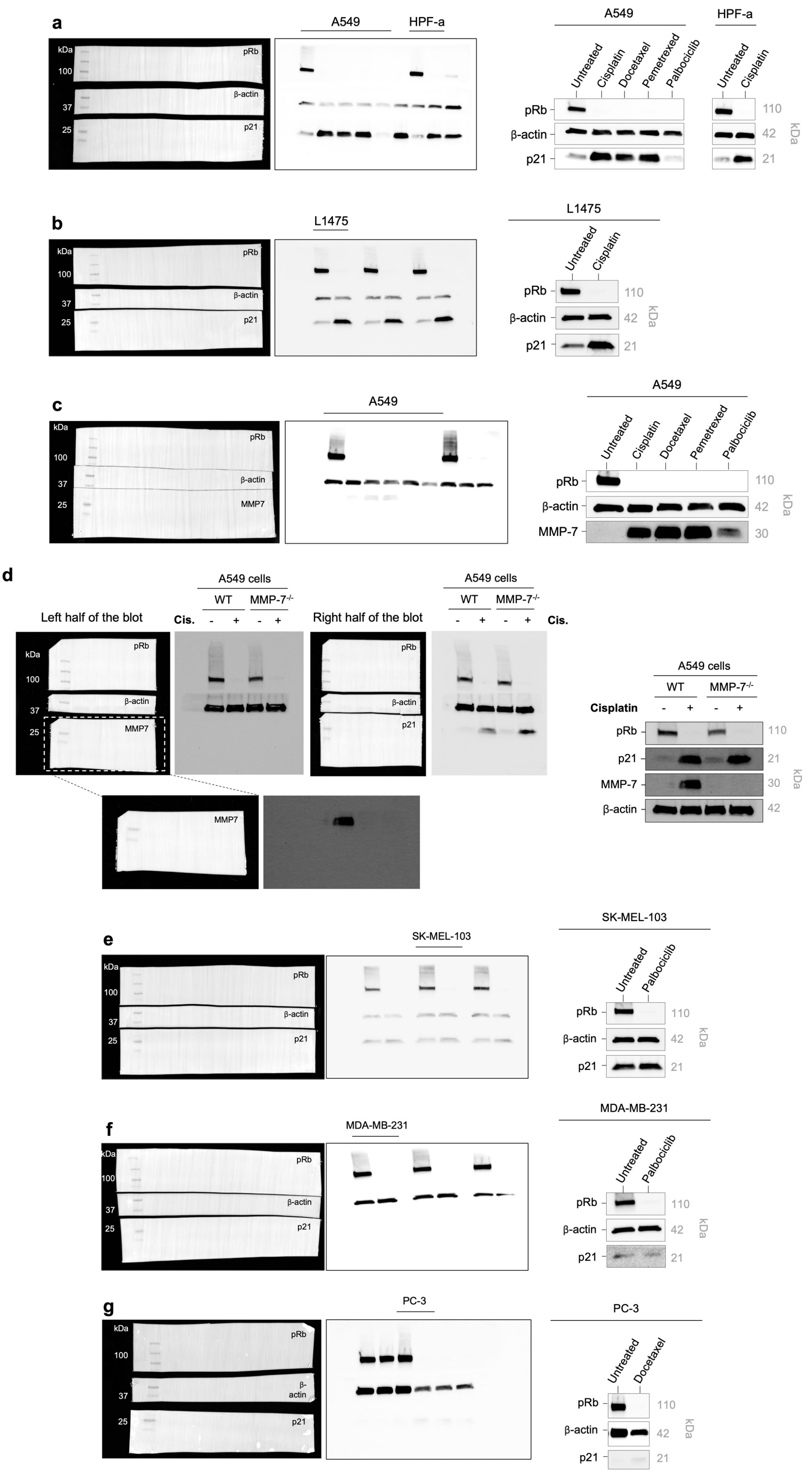
a
A549
HPF-a
kDa
100
37
25
pRb
β-actin
p21
Untreated
Cisplatin
Docetaxel
Pemetrexed
Palbociclib
110
42
21
b
L1475
c
MMP7
MMP-7
30
d
A549 cells
WT
MMP-7-/-
Left half of the blot
Right half of the blot
Cis.
Cisplatin
e
SK-MEL-103
f
MDA-MB-231
g
PC-3

Supplementary Fig. 2. Uncropped western blot for senescence validation and intracellular MMP-7 level. Analysis of senescence markers, pRb and p21, in (**a**) A549 (Supplementary Fig. 1b) and HPF-a cells (Supplementary Fig. 1i) and (**b**) L1475 cells (Supplementary Fig. 1g). Three biological replicates were performed for each cell line. **c**. Uncropped western blot for intracellular MMP-7 level in A549 cells (Supplementary Fig. 3d). **d.** Analysis of senescence markers, pRb and p21, and MMP-7 in Wild Type (WT) vs. MMP-7 deficient A549 cells (**Fig. 4g**). Analysis of senescence markers, pRb and p21, in (**e**) SK-MEL-103, (**f**) MDA-MB-231, and (**g**) PC-3 cells (Supplementary Fig. 10g). Three biological replicates were performed for each.

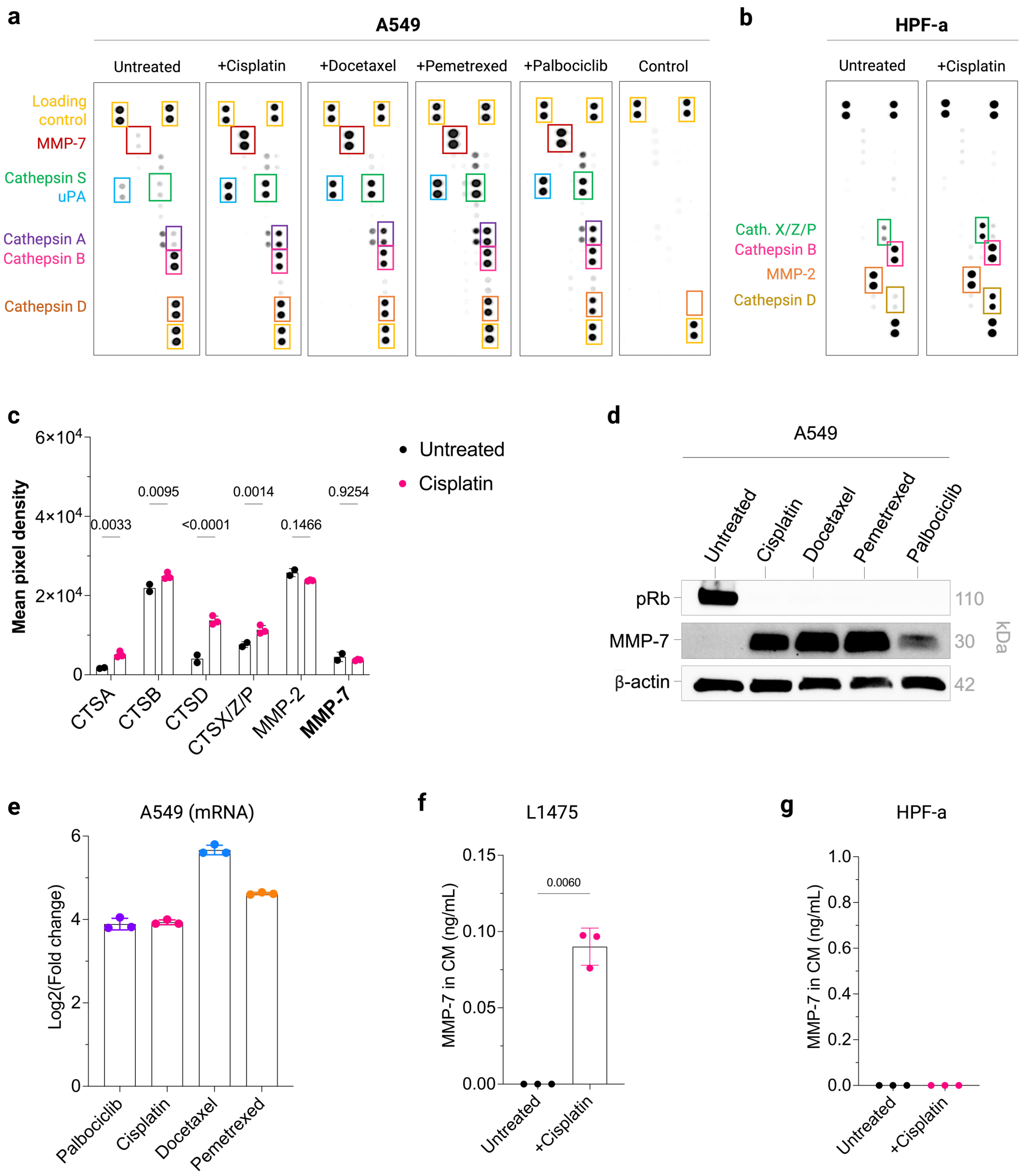

Supplementary Fig. 3. Senescent human and murine cancer cells abundantly secrete MMP-7 into the conditioned media, while fibroblast cells do not. a. Photograph of uncropped human protease array panels of conditioned media (CM) samples from untreated and treated A549 cells, as well as only media without cells (control). All blots are exposed for 2 min. **b**. Photograph of human protease array panels of CM samples from untreated and treated HPF-a cells. Both blots are exposed for 3 min. **c**. Pixel intensity z-score of secreted proteases corresponding to (**b**) (*N* = 3, mean ± s.d.). **d**. Western blot analysis of pro-MMP-7 level (28 kDa) from lysates of senescent and non-senescent A549 cells. **e**. Log2 fold change gene expression levels of *MMP7* in treated A549 cells relative to untreated cells (*N* = 3). Concentration of MMP-7 in the CM of untreated (non-senescent) and treated (senescent) (**f**) L1475 cells (N = 3, mean ± s.d.; t-test) and (**g**) HPF-a cells. pRb: hyperphosphorylated Retinoblastoma.

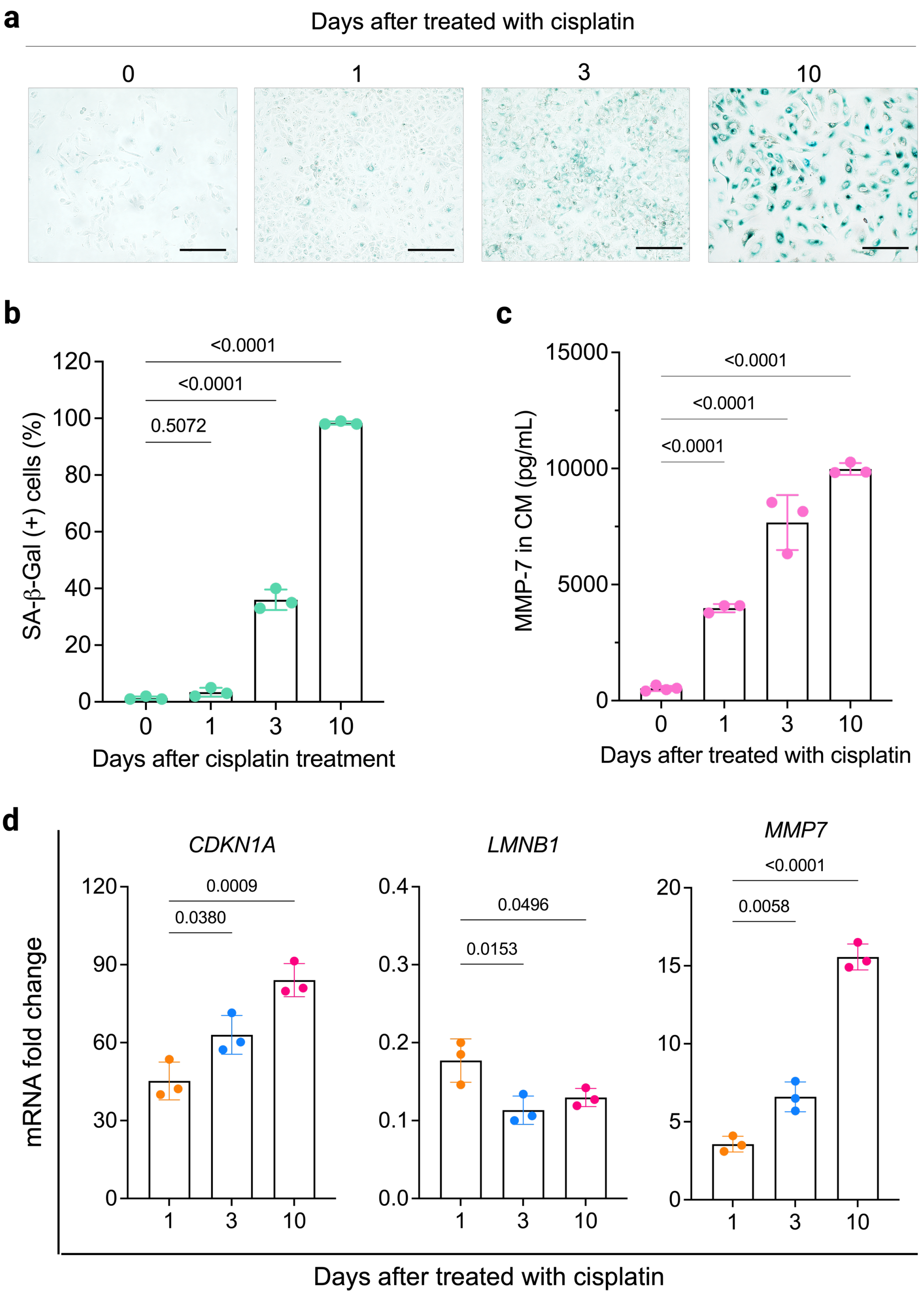

Supplementary Fig. 4. **Kinetics of MMP-7 secretion during senescence. a**. SA-β-Gal staining of untreated (non-senescent) and treated (senescent) A549 cells after different days of cisplatin treatment, with the corresponding quantification in (**b**) (mean ± s.d., N = 3 independent experiments, ordinary one-way ANOVA with Dunnett's multiple comparisons test). **c**. Concentration of MMP-7 in the CM of untreated (non-senescent) and treated (senescent) A549 cells after different days of cisplatin treatment. Data are shown as mean ± SD (N = 3, ordinary one-way ANOVA with Dunnett's multiple comparisons test). **d**. Log2 fold change gene expression levels of senescence markers and *MMP7* in A549 cells after different days of cisplatin treatment (N = 3, ordinary one-way ANOVA with Dunnett's multiple comparisons test).

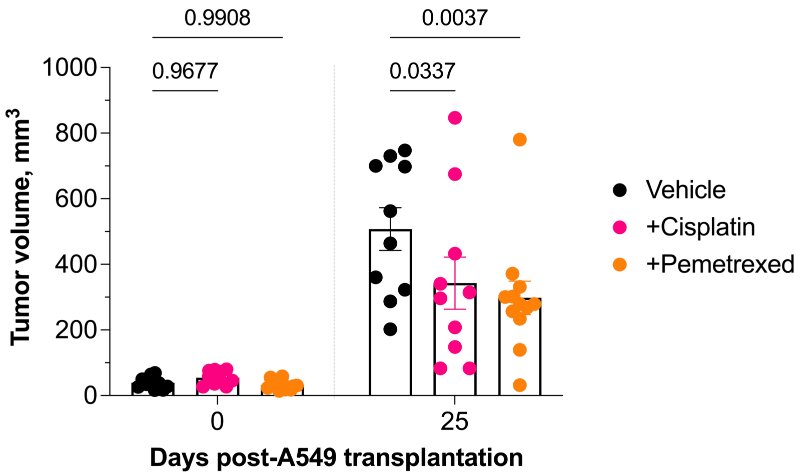

Supplementary Fig. 5. **Drug-treated mice showed reduced tumor growth, partially due to senescence induction.** Tumor volume at the start of the experiment (day 0) versus at the endpoint of treatment (day 25). Data is presented as mean ± s.d.; one-way ANOVA with Dunnett's multiple comparisons test.

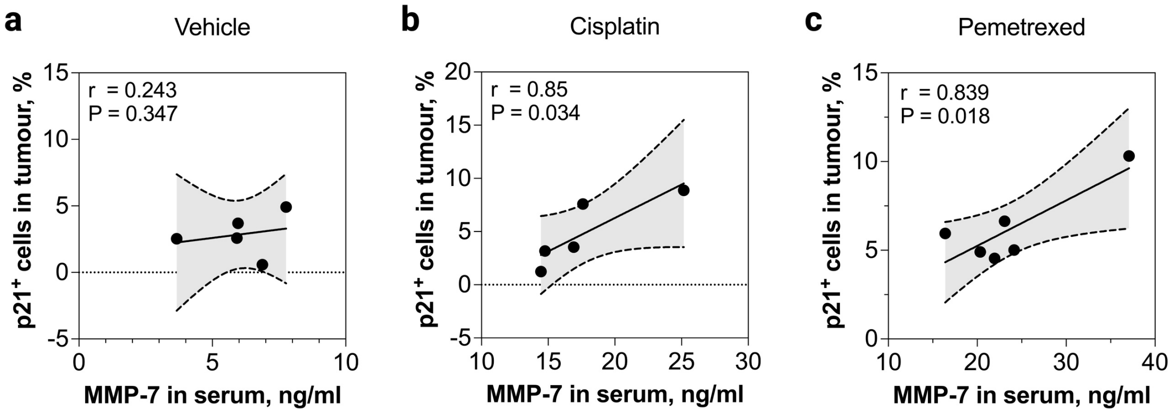

Supplementary Fig. 6. **Higher $p21^+$ levels in tumors correlated positively with higher levels of circulating MMP-7 in the serum of drug-treated mice.** Plot of $p21^+$ cells in the tumor versus MMP-7 level in the serum for (**a**) vehicle, (**b**) cisplatin, and (**c**) pemetrexed-treated mice. Statistics calculated using the two-tailed Pearson correlation.

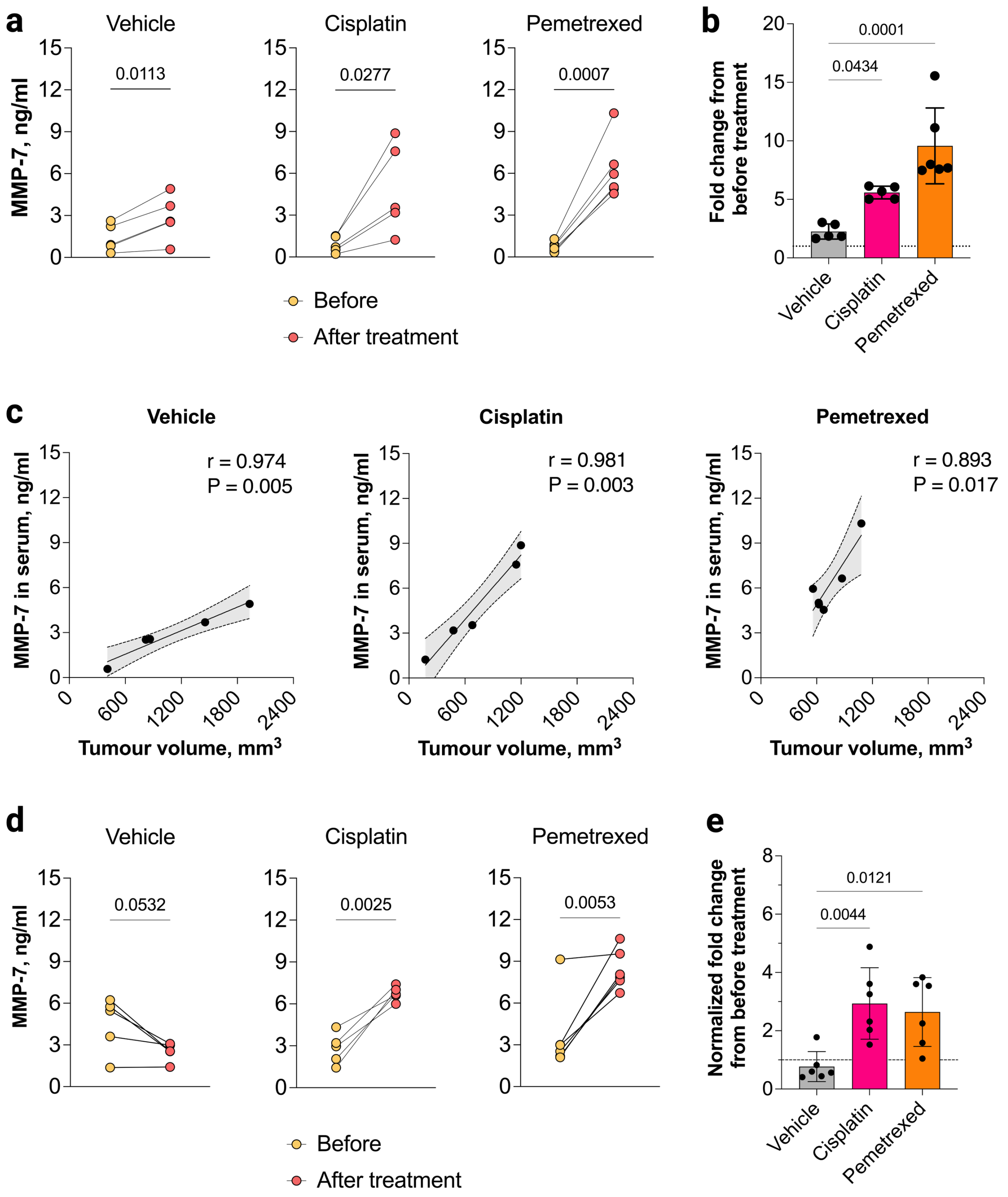

Supplementary Fig. 7. **Cisplatin and pemetrexed-treated mice had a significantly higher increase in MMP-7 level in their serum following treatment. a**. Serum concentration of MMP-7, before and after treatment (paired t-test). **b**. Fold change of serum concentration of MMP-7 before and after treatment (N = 5 mice for vehicle and cisplatin, N = 6 mice for pemetrexed, mean ± s.d.; ordinary one-way ANOVA with Dunnett's test multiple comparisons). **c**. Plot of MMP-7 in the serum versus tumor volume. MMP-7 level in the serum positively correlates with tumor volume for all groups. Statistics calculated using the two-tailed Pearson correlation. **d**. Concentration of MMP-7 in the serum, before and after treatment, normalized to tumor volume before and after treatment (paired t-test). **e**. Fold change of concentration of MMP-7 in the serum before and after treatment normalized to tumor volume (mean ± s.d.; ordinary one-way ANOVA with multiple comparisons).

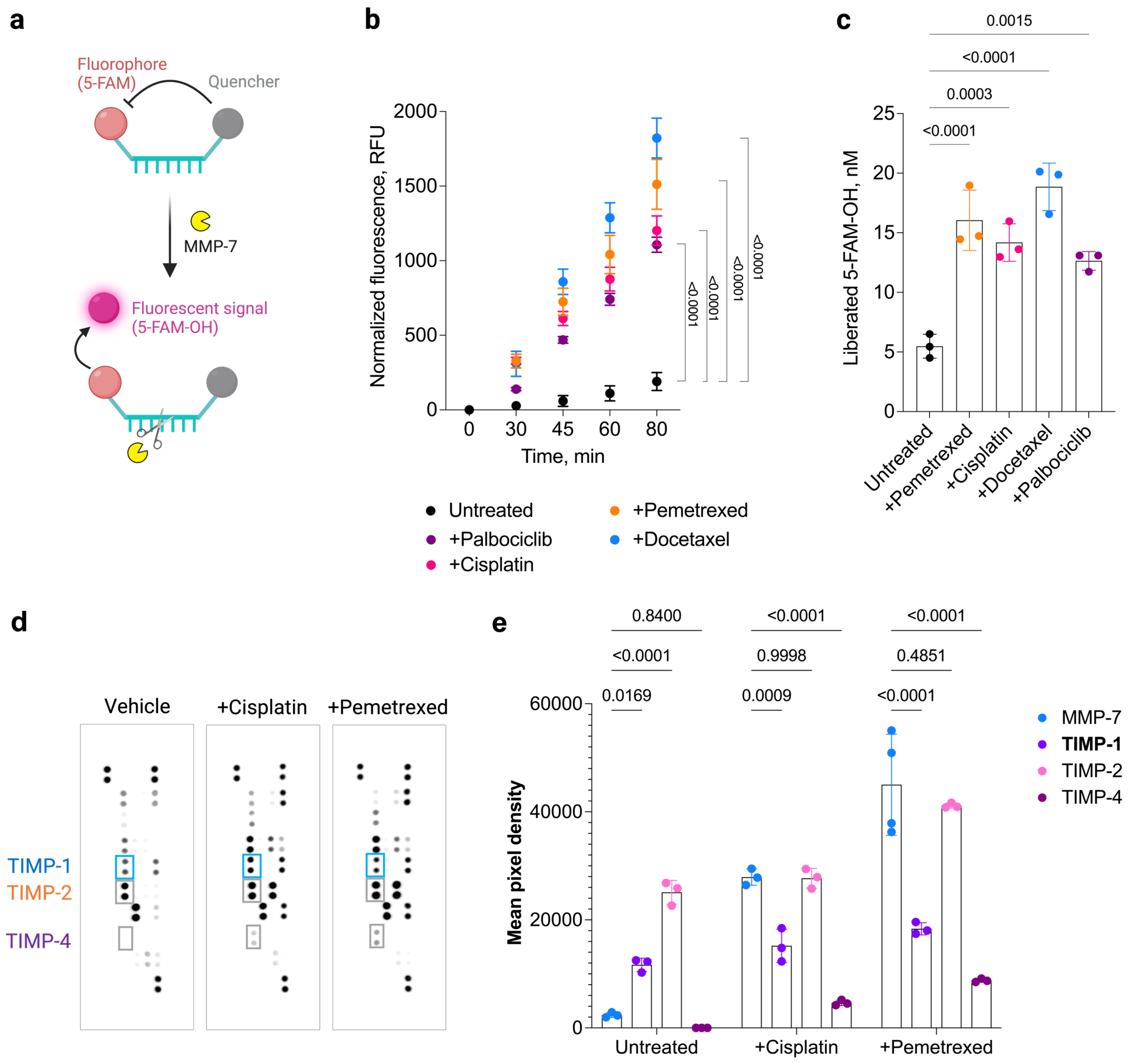

Supplementary Fig. 8. MMP-7 in the CM of senescent A549 cells was enzymatically active. a. Schematic illustration of the commercial MMP-7 activity assay used in this study. **b**. Secreted MMP-7 was active as enzyme, as measured by fluorescence-based assay (N = 3 independent experiments, mean ± s.d.; two-way ANOVA with Dunnett's multiple comparisons test). **c**. Graph of fluorophore liberated due to MMP-7 activity (N = 3, mean ± s.d.; one-way ANOVA with multiple comparisons test). **d**. Photograph of human protease inhibitor array panels of CM samples from untreated and treated A549 cells. All blots were exposed for 2 min. **e**. Pixel intensity z-score of secreted TIMP corresponding to (a) (N = 3, mean ± s.d.; two-way ANOVA with Dunnett's multiple comparisons test). Note that MMP-7 level was taken from a different blot shown in Supplementary Fig. 3.

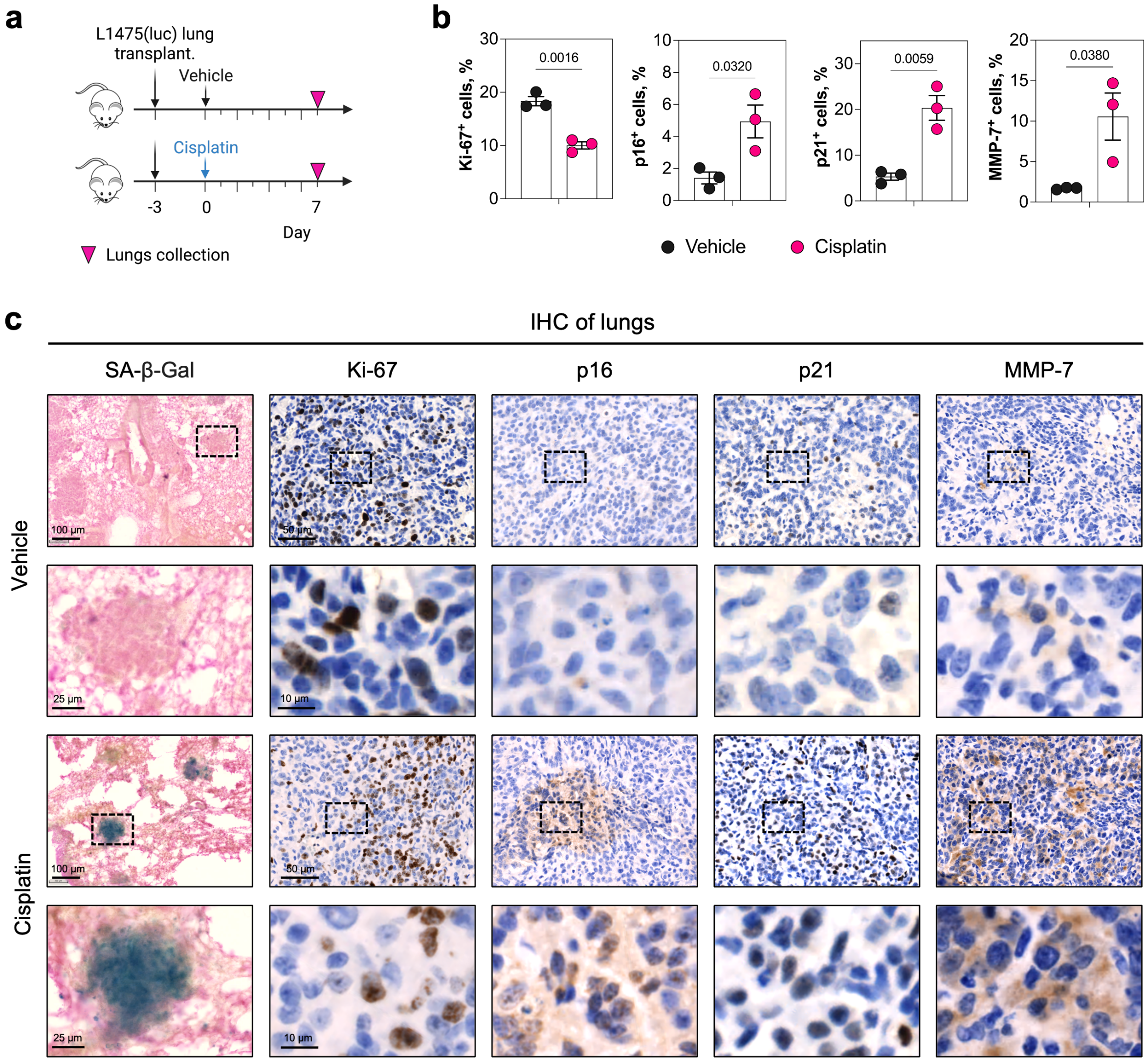

Supplementary Fig. 9. Cisplatin treatment induces tumor senescence in orthotopically transplanted lung cancer mouse models, accompanied by a higher MMP-7 level. a. Mice were transplanted with L1475(luc) cells in the lung via tail-vein injection, and after 3 days, they were subjected to 1.5 mg/kg body CDDP or vehicle as shown in the timeline. Lungs were resected at day 7 post-transplantation. **b**. Histological quantification and (**c**) representative histological images for SA-β-gal activity, Ki-67, p16, p21, and MMP-7 staining of the lungs in the indicated experimental groups. Scale bars: 100 μm, 50 μm, 25 μm, or 10 μm as indicated. Data is presented as mean ± s.d., unpaired t-test.

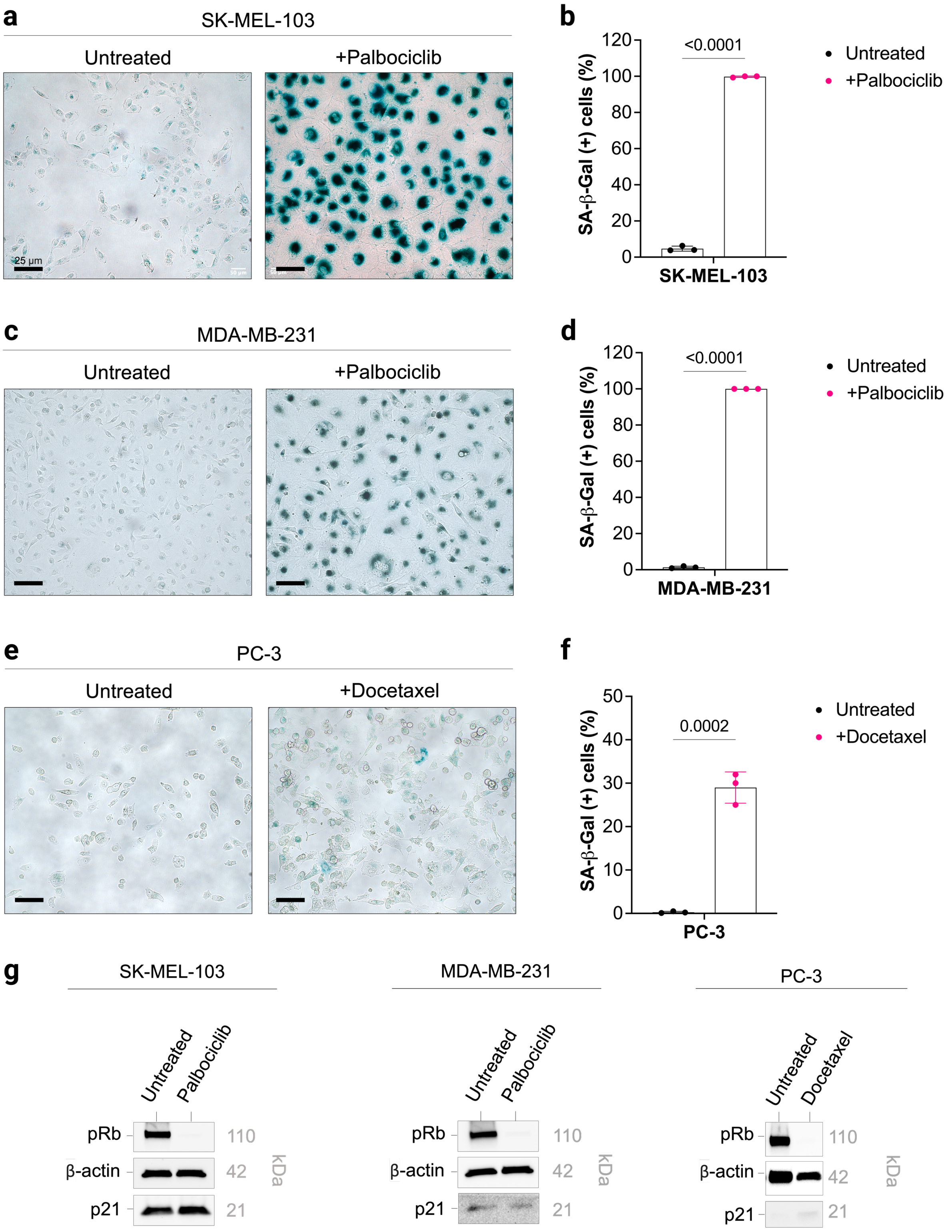

Supplementary Fig. 10. Validation of senescence in other cancer cell lines. Representative images of untreated (non-senescent) and chemotherapy-treated (senescent) cells fixed and stained for SA-β-Gal activity after 10 days of treatment; (**a-b**) for SK-MEL-103 cells, (**c-d**) for MDA-MB-231 cells, and (**e-f**) for PC-3 cells. Data is presented as mean ± s.d. (n = 3 biological replicates with three technical replicates each, unpaired t-test). Scale bar = 25 µm. **g**. Western blot for the expression of relevant senescence markers in control and senescent SK-MEL-103, MDA-MB-231 and PC-3 cells.

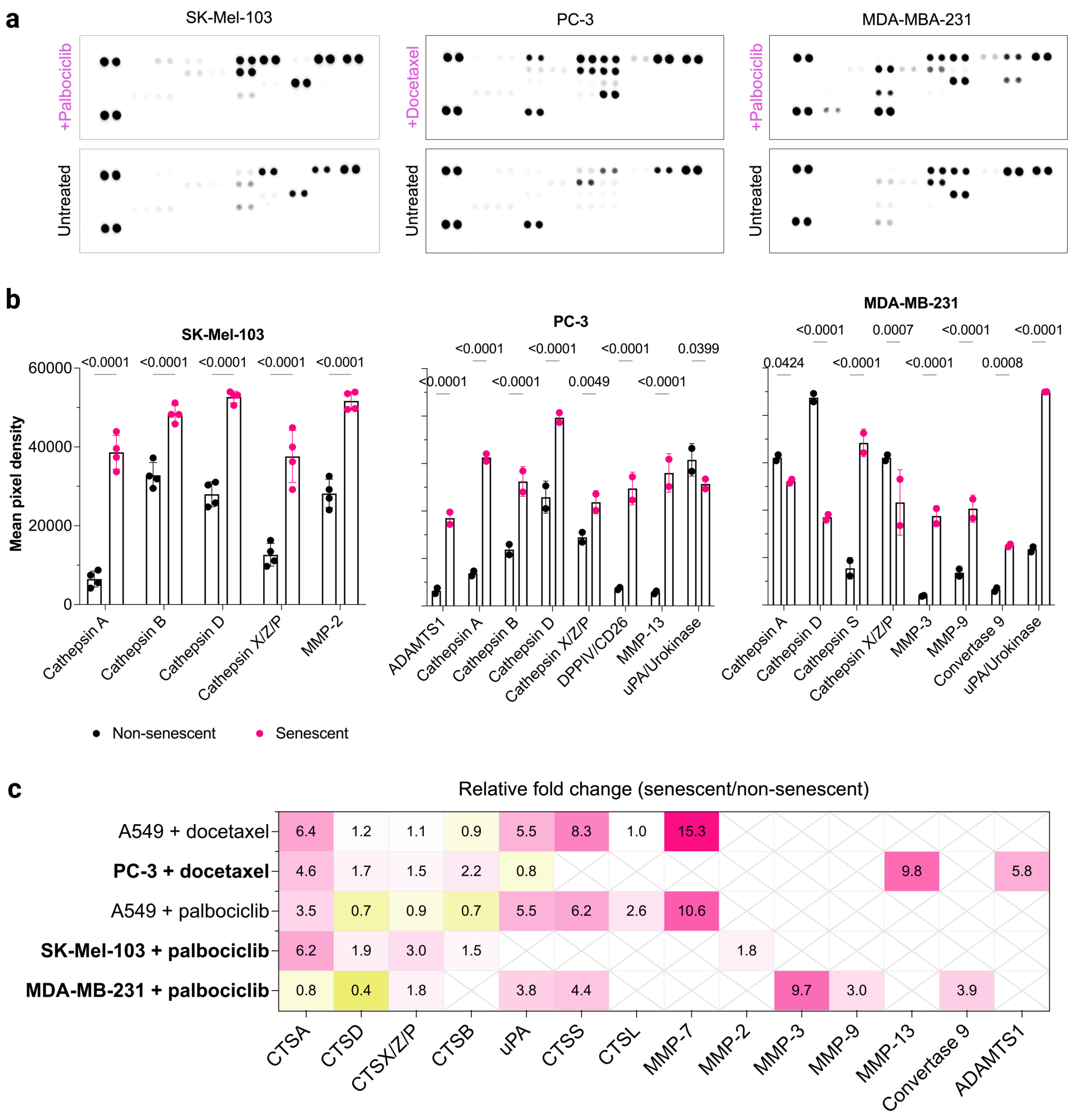

Supplementary Fig. 11. Different human cancer cells secrete distinct proteases when undergoing senescence. a. Photograph of human protease array panels of conditioned media (CM) samples from untreated (non-senescent) and drug-treated (senescent) Sk-Mel-103, PC-3, and MDA-MB-231 cells. All blots were exposed for 2 min. **b**. Relative level of proteases secreted by chemotherapy-induced senescent and non-senescent cells corresponding to (a) (n = 2 biological replicates, mean ± s.d.; two-way ANOVA with Dunnett's multiple comparisons test) **c**. Fold change of proteases in the CM of senescent versus non-senescent cells corresponding to (b).

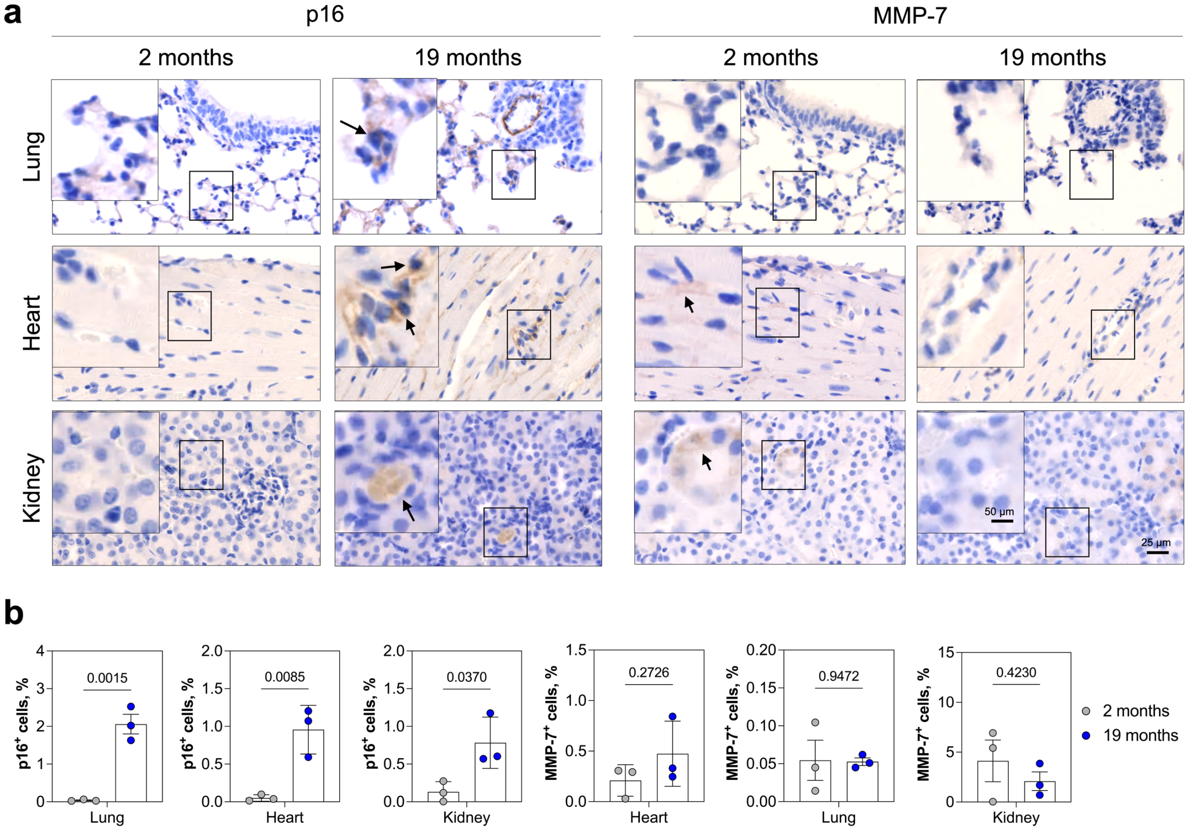

Supplementary Fig. 12. Histological images of lungs, heart, and kidney obtained from 2-month-old vs 19-month-old C57BL/6 mice. a. Representative staining for p16 and MMP-7 (consecutive sections). **b**. Quantification of the staining. Scale bars = 25 and 50 μm. N = 3 mice for each group, mean ± s.d., unpaired t-test. Arrows shows marker-positive cells.

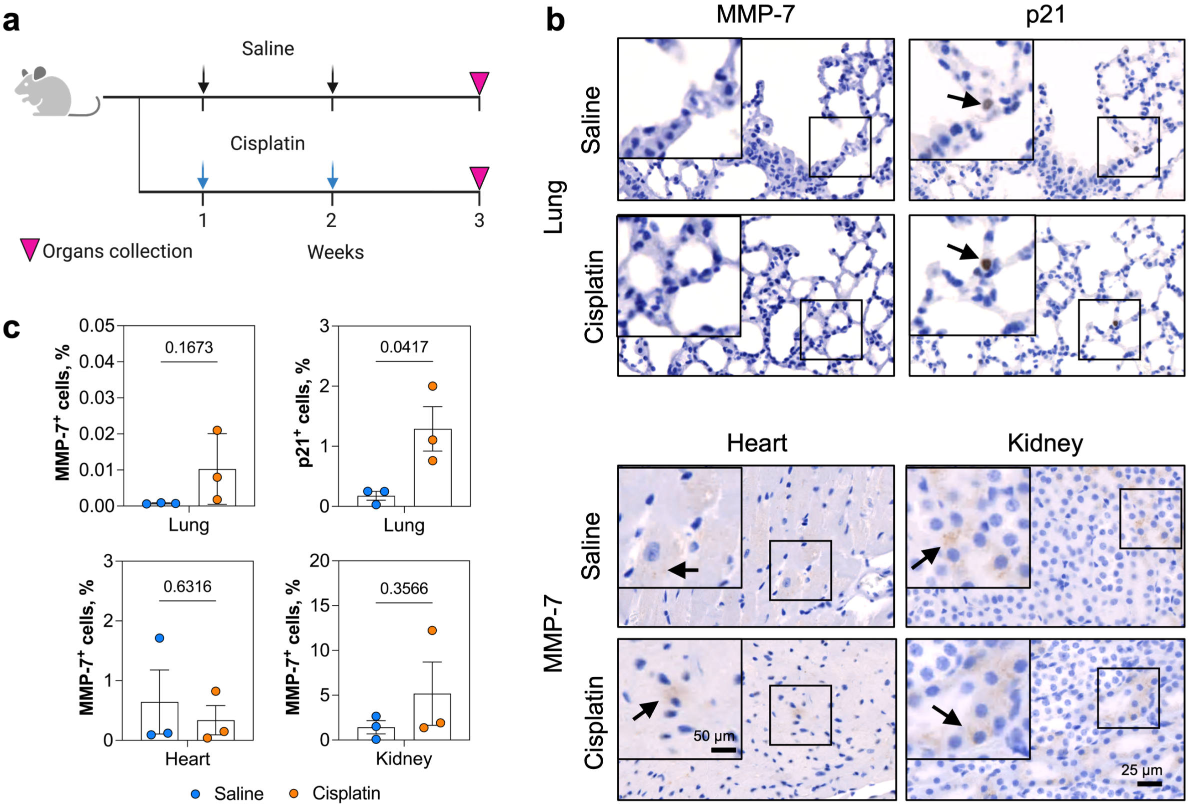

Supplementary Fig. 13. Histological images of lungs, heart, and kidney obtained from non-tumour-bearing, cisplatin- vs vehicle-treated C57BL/6 mice. a. Mice were treated with either two cycles of cisplatin or with saline. **b**. Representative staining for the lungs with p21 and MMP-7 (consecutive sections), and the heart and kidney with MMP-7. **c.** Quantification of the staining. Scale bars = 25 and 50 µm. N = 3 mice for each group, mean ± s.d., unpaired t-test. Arrows shows marker-positive cells.

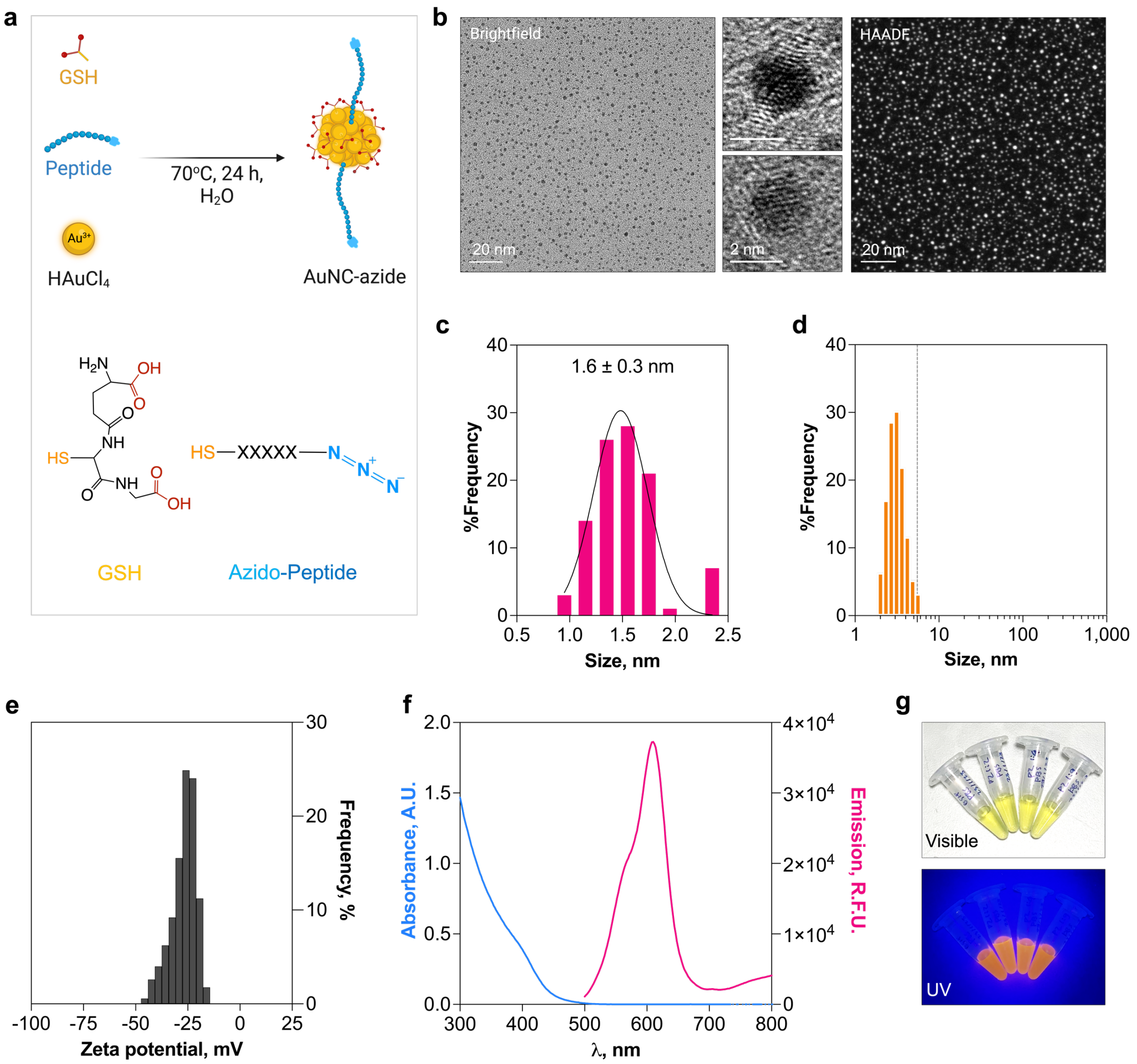

Supplementary Fig. 14. **Preparation and characterization of AuNC-azide. a**. Schematic illustration of AuNC-azide preparation and molecular structure of GSH and azidopeptide. Note that both GSH and azidopeptides have thiol (-SH) groups for bonding with Au. **b**. TEM images of AuNC in brightfield and HAADF mode. Scale bar = 20 nm and 2 nm. **c**. Histogram of size distribution of AuNC measured from TEM (N = 200 particles). **d**. Hydrodynamic size of AuNC measured from DLS. **e**. Surface charge or zeta potential of AuNC in PBS. **f**. UV-Vis absorbance and emission spectra of AuNC in PBS ($\lambda_{excitation}$ = 400 nm). **g**. Photographs of AuNC suspension in PBS under visible and UV light.

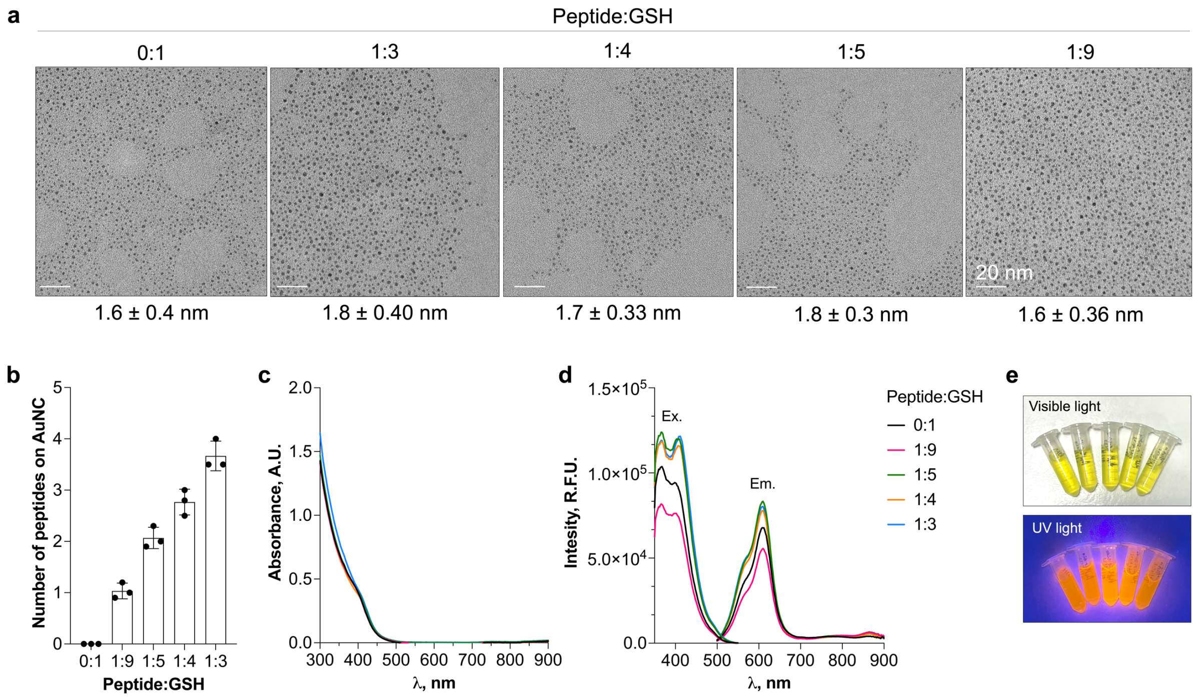

Supplementary Fig. 15. **AuNC-azide with varying peptide content.** **a**. TEM images of AuNC with different amount peptide loading (N = 200 particles, mean ± s.d.). **b**. Estimated number of attached peptides in AuNC for different loading amount (N = 3 independent experiments, mean ± s.d.). UV-Vis absorbance (**c**) and excitation and emission (**d**) spectra of AuNC with different amount peptide loading, with the corresponding photographs of the suspension in (**e**).

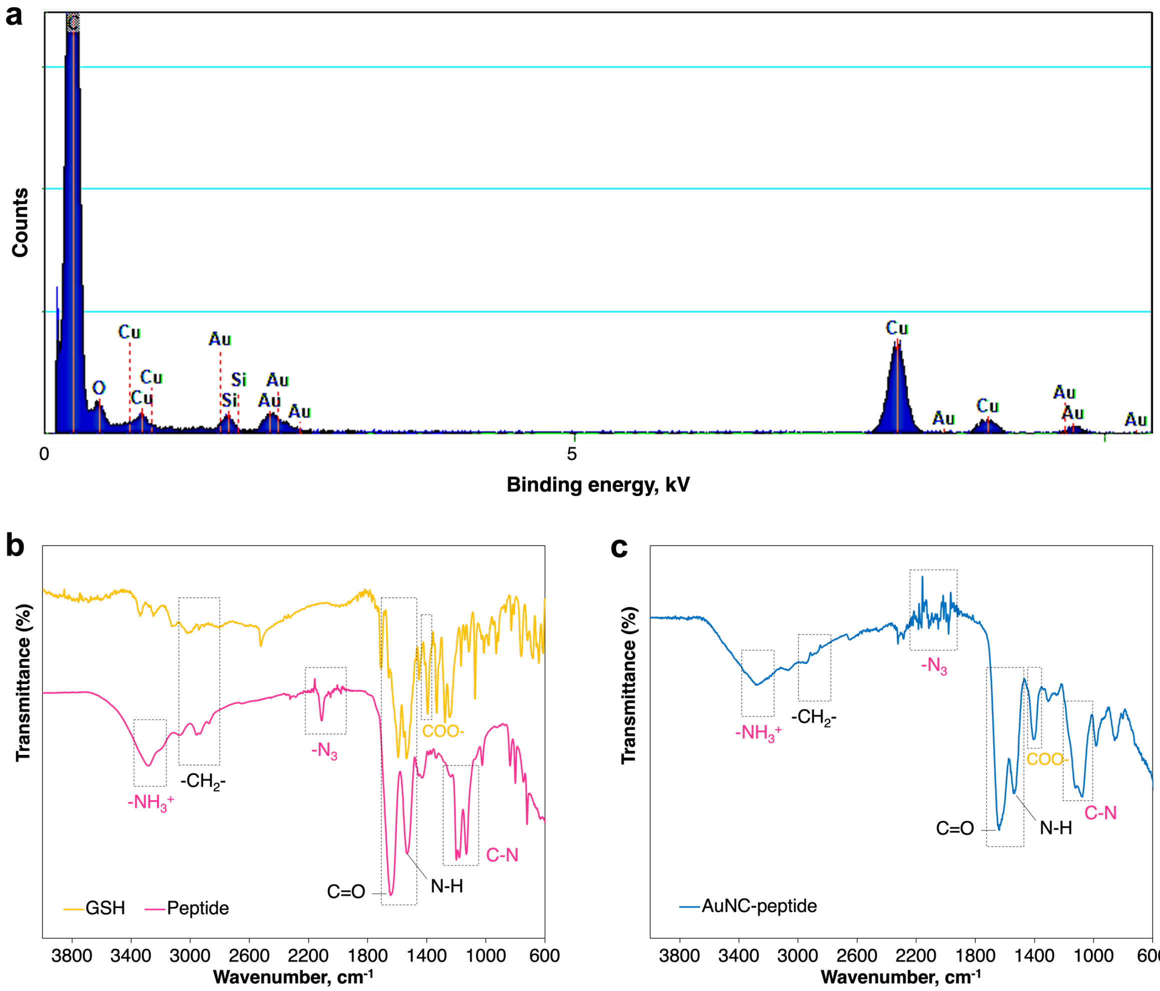

Supplementary Fig. 16. Elemental analysis of AuNC-azide. **a**. Energy Dispersive X-ray (EDX) spectra analysis of AuNC, showing the presence of gold and copper from the grid. **b**. Fourier-Transform Infrared (FT-IR) spectrum of GSH and azidopeptide. **c**. FT-IR spectrum of AuNC peptide, showing the presence of characteristic peaks corresponding to the attached GSH and azidopeptide.

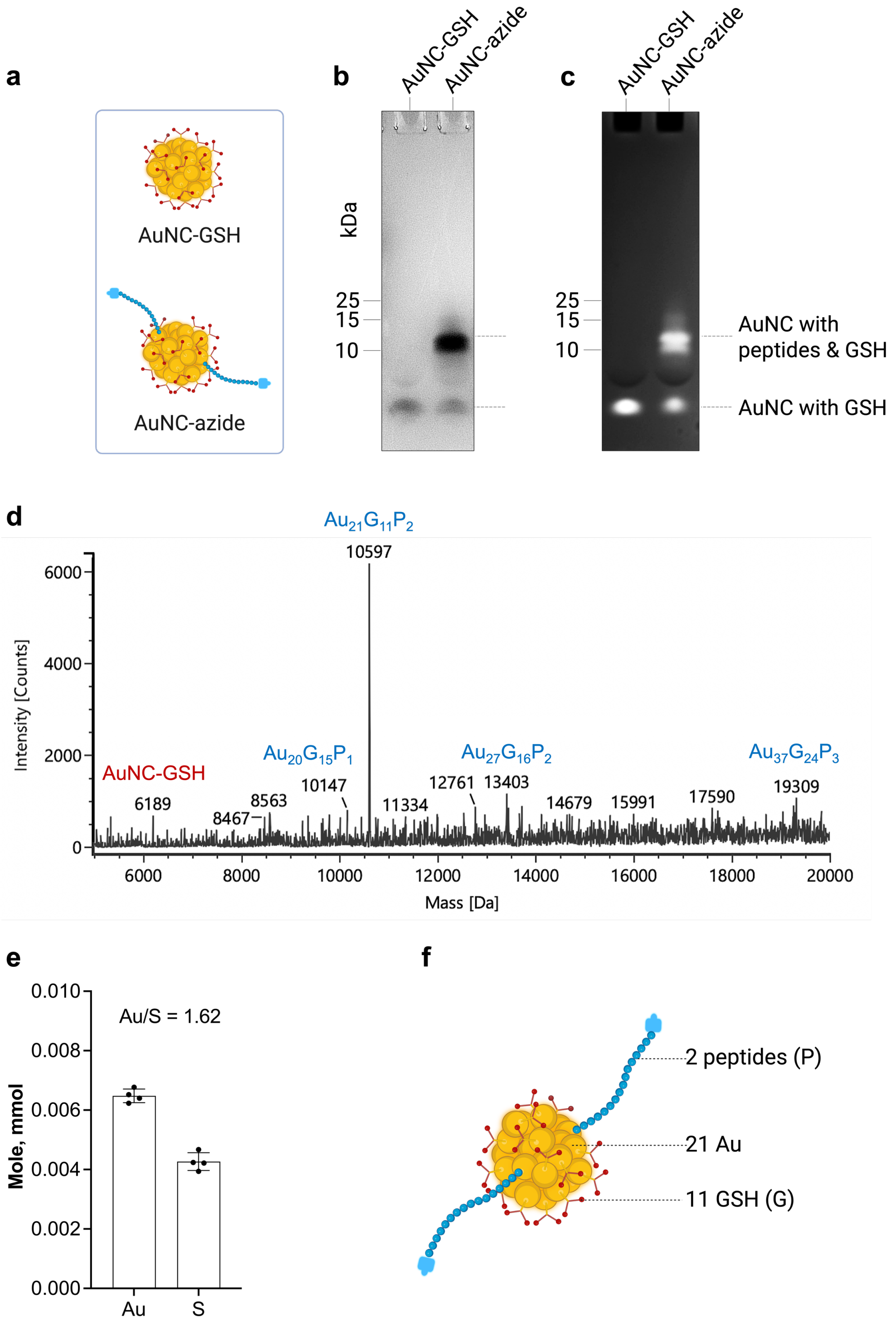

Supplementary Fig. 17. **Molar mass determination of AuNC-azide. a.** Schematic of AuNC-GSH and AuNC-azide. PAGE of AuNC stained with (**b**) Coomassie and (**c**) luminol showing an estimated molecular mass of around 10-15 kDa, corroborating to the calculated mass from LC-MS (**d**). **e**. Au and S content of AuNC measured from ICP (N = 3 independent experiments, mean ± s.d.). **f**. Estimated number of peptides, Au and GSH in AuNC determined from LC-MS and ICP results.

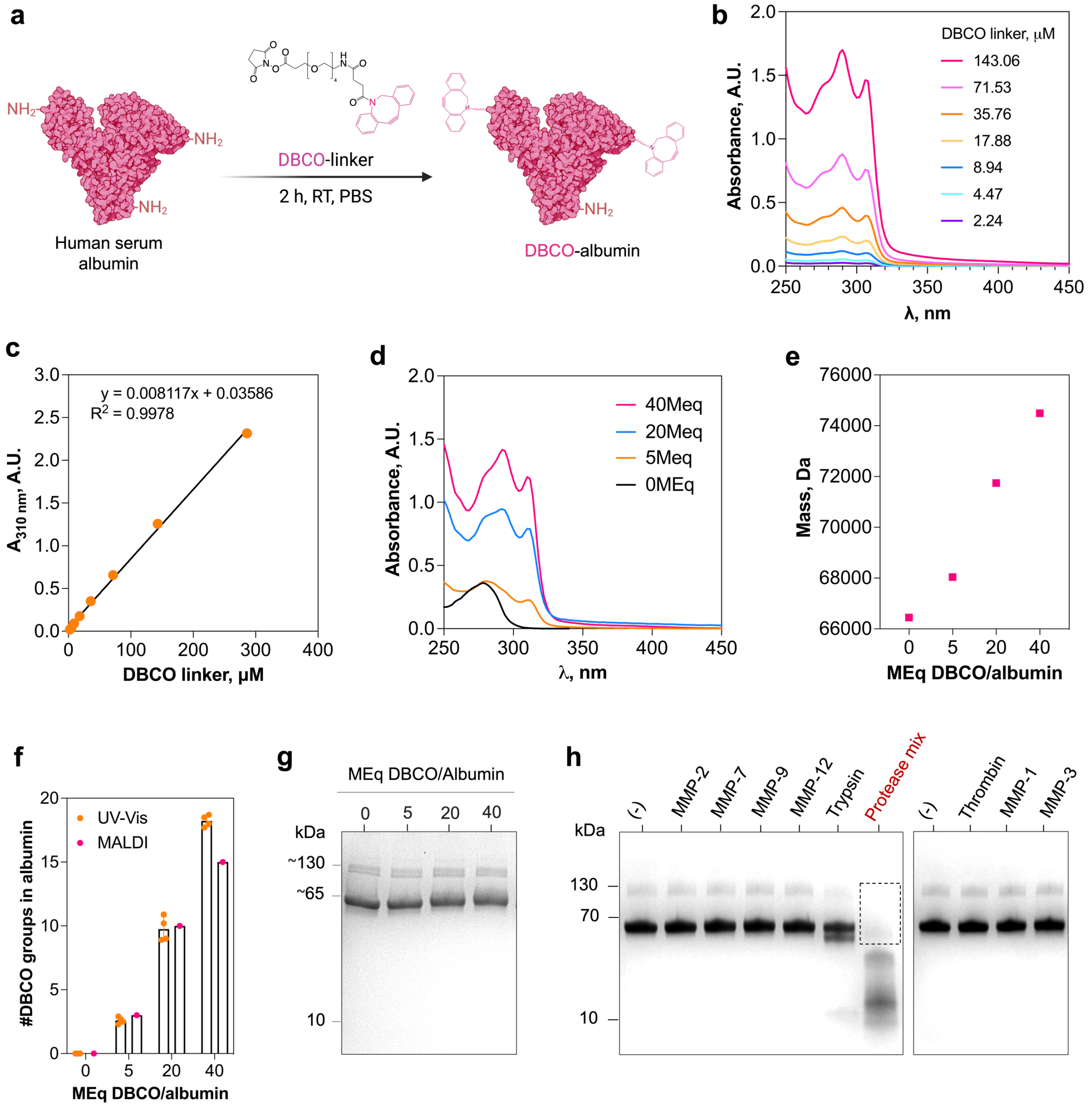

Supplementary Fig. 18. Functionalizing albumin with DBCO groups. a. Schematic illustration of functionalization of human serum albumin with DBCO groups. **b**. UV-Vis absorbance spectra of DBCO linker in varying concentration. **c**. Calibration curve to quantify the concentration of attached DBCO based on absorbance at $\lambda = 310$ nm (N = 3, mean ± s.d.). **d**. UV-Vis absorbance spectra of albumin (in PBS) after conjugation reaction with varying molar equivalent (MEq) of DBCO-linker. Experiment was repeated independently 3 times with similar result. **e**. Calculated molar mass of albumin (using MALDI) after conjugation reaction with varying MEq of DBCO-linker. Higher MEq of DBCO linker resulted in higher absorbance peak at 310 nm and higher molar mass, indicating successful attachment of DBCO groups. Full spectra is available in Supplementary Fig. 37. **f**. Estimated number of DBCO groups attached to albumin after conjugation reaction, calculated from UV-Vis spectroscopy (**b-d**) and MALDI analysis (**f**) (N = 3, mean ± s.d.). **g**. Gel electrophoresis of non-functionalized and DBCO-functionalized albumin. Functionalization with DBCO did not cause aggregation or fragmentation of albumin. **h**. Gel electrophoresis of DBCO-functionalized albumin (prepared from 40 MEq DBCO-linker) after 3 h incubation with different proteases (10 μM) at 37◦C, including protease mix (100 μg/ml) as a positive control.

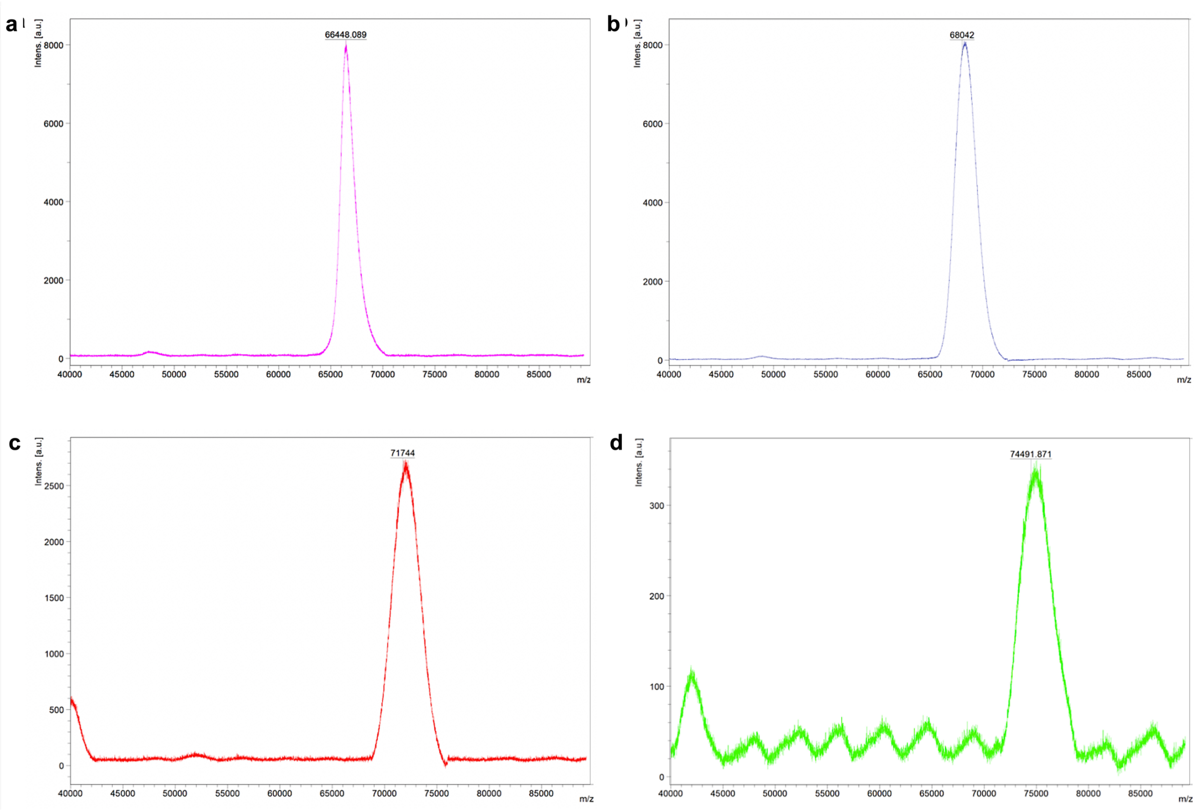

Supplementary Fig. 19. MALDI spectra of DBCO-functionalized albumin. Spectra of (**a**) native albumin and albumin functionalized with (**b**) 5, (**c**) 20 and (**d**) 40 molar equivalents of DBCO-NHS esters. The corresponding peaks: m/z = 66448.089, 68042, 71744, 74491.871, respectively.

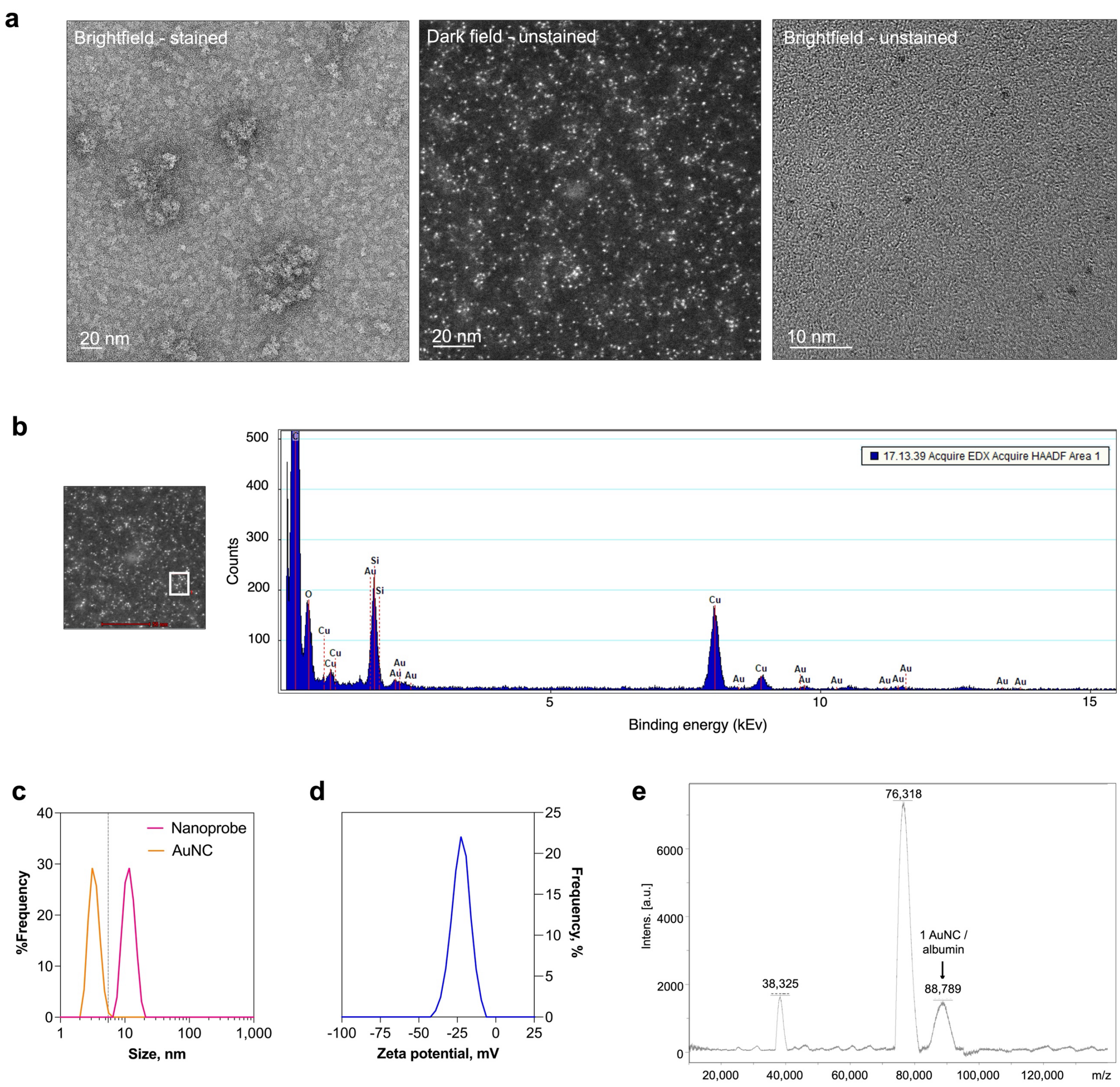

Supplementary Fig. 20. Characterization of ALBANC nanoprobe. **a**. TEM images of nanoprobe in brightfield mode after staining with uranyl acetate (left), in HAADF mode (middle) and in brightfield mode unstained (right). **b.** EDS spectrum of nanoprobe, showing the presence of Au. **c.** Histogram plotting the hydrodynamic size of AuNC and nanoprobe in PBS. Experiment was repeated independently 3 times with similar results. **d**. Surface charge of AuNC and nanoprobe. Experiment was repeated independently 3 times with similar results. **e**. LC-MS spectrum of nanoprobe, showing peaks corresponding to unbound DBCO-albumin and the nanoprobe peak in which albumin is conjugated to one AuNC.

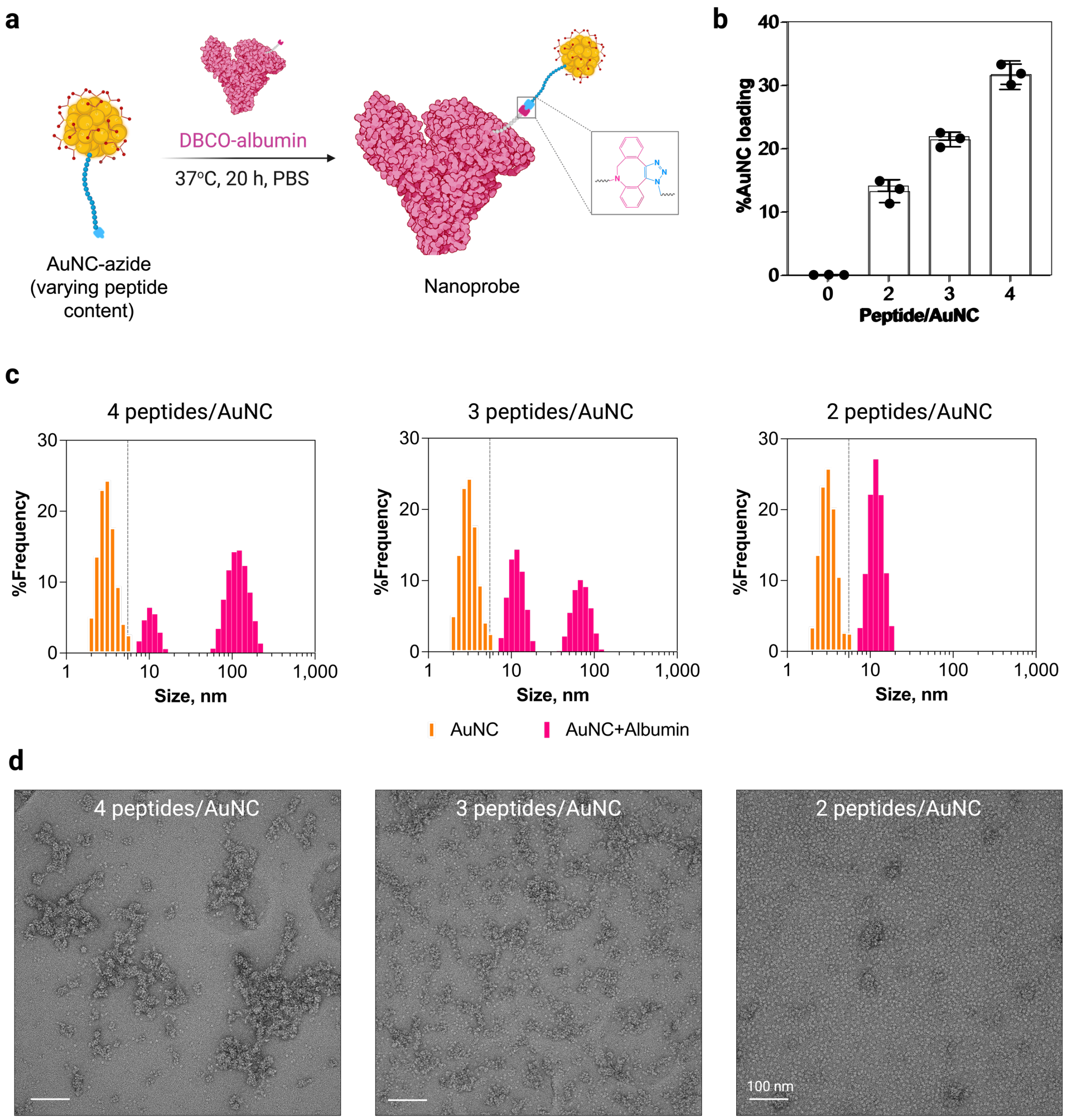

Supplementary Fig. 21. **Preparation of nanoprobe using AuNC with different peptide amount. a.** Schematic illustration of nanoprobe preparation. **b.** Percentage loading of AuNC to DBCO-albumin as a function of the peptide amount in AuNC (N = 2, mean ± s.d.). Hydrodynamic size (**c**) and TEM image of nanoprobe prepared using AuNC with different peptide amount (**d**). Although using AuNC that has more peptides resulted in higher %AuNC loading, it produced nanoprobe that were more prone to aggregation as observed from DLS and TEM, possibly due to crosslinking of the nanoprobe as one AuNC could be attached to multiple albumins. In contrast, conjugation using AuNC with 2 peptides resulted in nanoprobe with minimum aggregation. From here it was decided that AuNC with 2 peptides were used to make the nanoprobe.

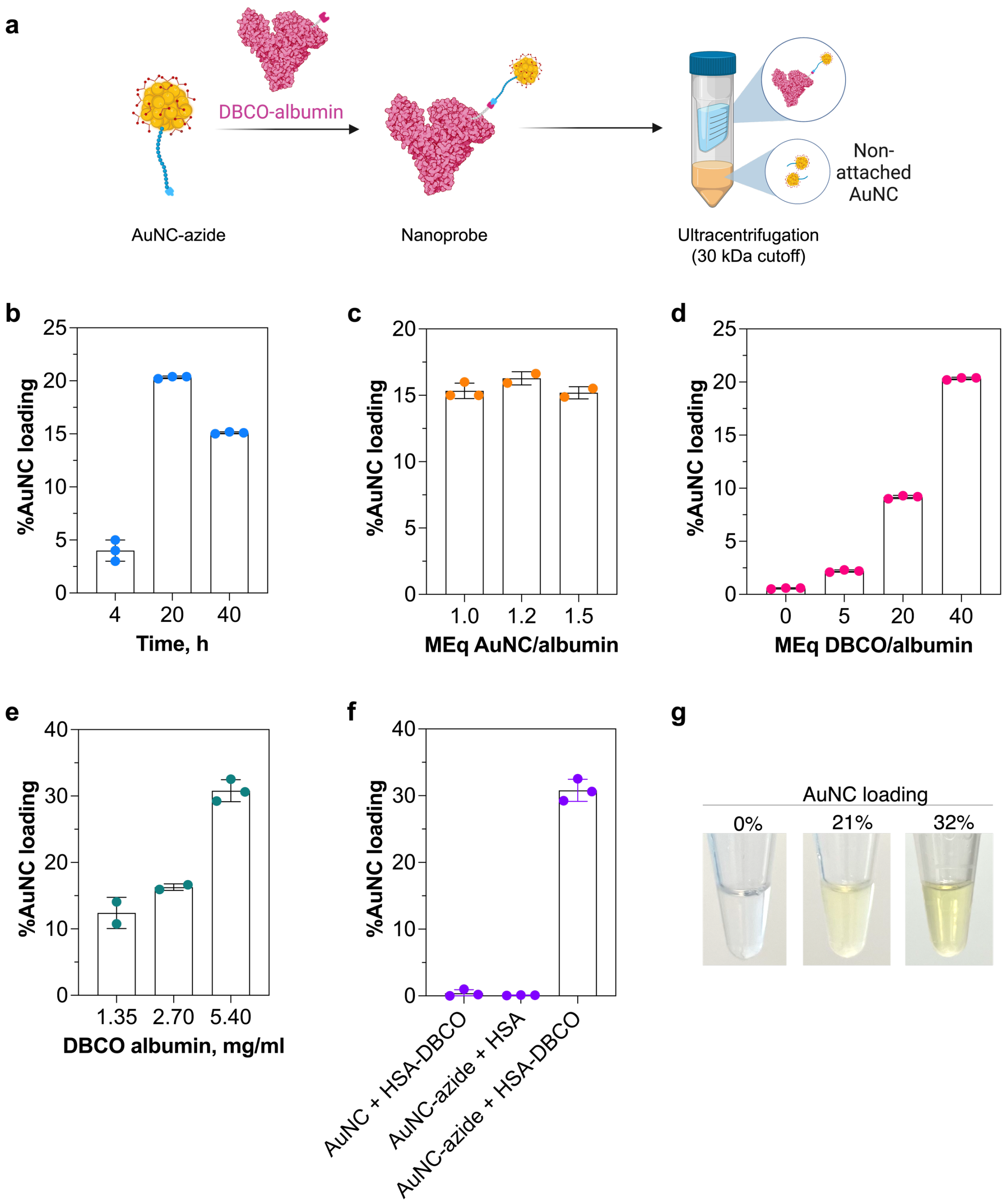

Supplementary Fig. 22. **Optimization of AuNC loading in ALBANC nanoprobe. a.** Schematic illustration of the nanoprobe preparation and purification. Optimization included varying conjugation reaction time (**b**), molar equivalent (MEq) of AuNC to albumin (**c**), MEq of DBCO-albumin (**d**), and (**e**) the concentration of DBCO-albumin (N = 3 independent experiments, mean ± s.d.). AuNC loading into DBCO was measured by calculating the ratio of the concentration of unbound AuNC (in the supernatant after ultrafiltration) to the initial AuNC concentration. **f**. Control experiments showing AuNC (without azidopeptide) + albumin-DBCO and AuNC-azide + albumin (without DBCO) resulted in negligible AuNC loading, indicating negligible non-specific binding of AuNC to albumin (and therefore conjugation only occurred *via* DBCO-azide click chemistry). **g**. Photograph of nanoprobe suspension in PBS with different AuNC loading. Yellowish color arose from the attached AuNC. Note: all experiments used AuNC with 2 peptides (molar ratio of peptide:GSH = 1:5).

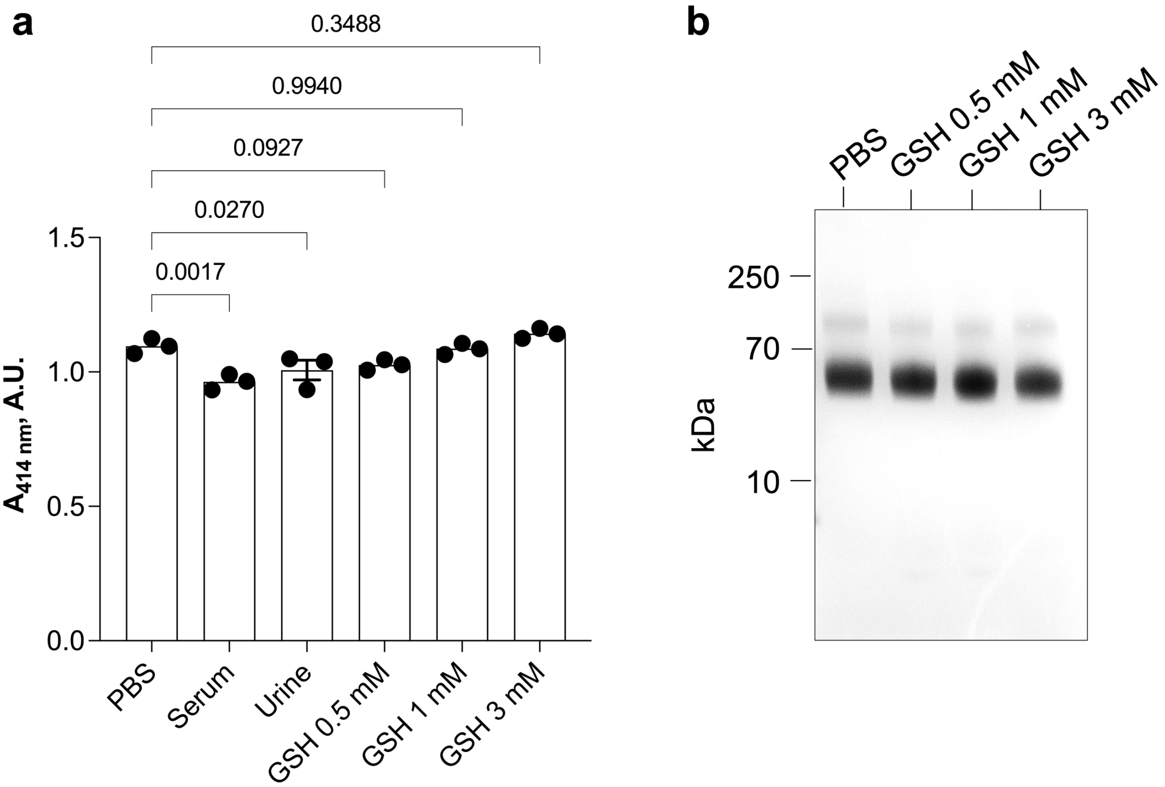

Supplementary Fig. 23. **Stability of AuNC and ALBANC nanoprobe. a.** Alloy formation assay of AuNC (GSH:peptide 1:5 molar ratio) incubated with either PBS, urine or physiological concentrations of GSH for 2 h at 37 ºC (mean ± s.d., N = 3, one-way ANOVA). **b**. Comassie-stained gel electrophoresis of ALBANC nanoprobe after being incubated with either PBS or physiological concentrations of GSH for 2 h at 37 ºC (GSH was removed from the nanoprobe by washing with 10 kDa Amicon centrifugal filter).

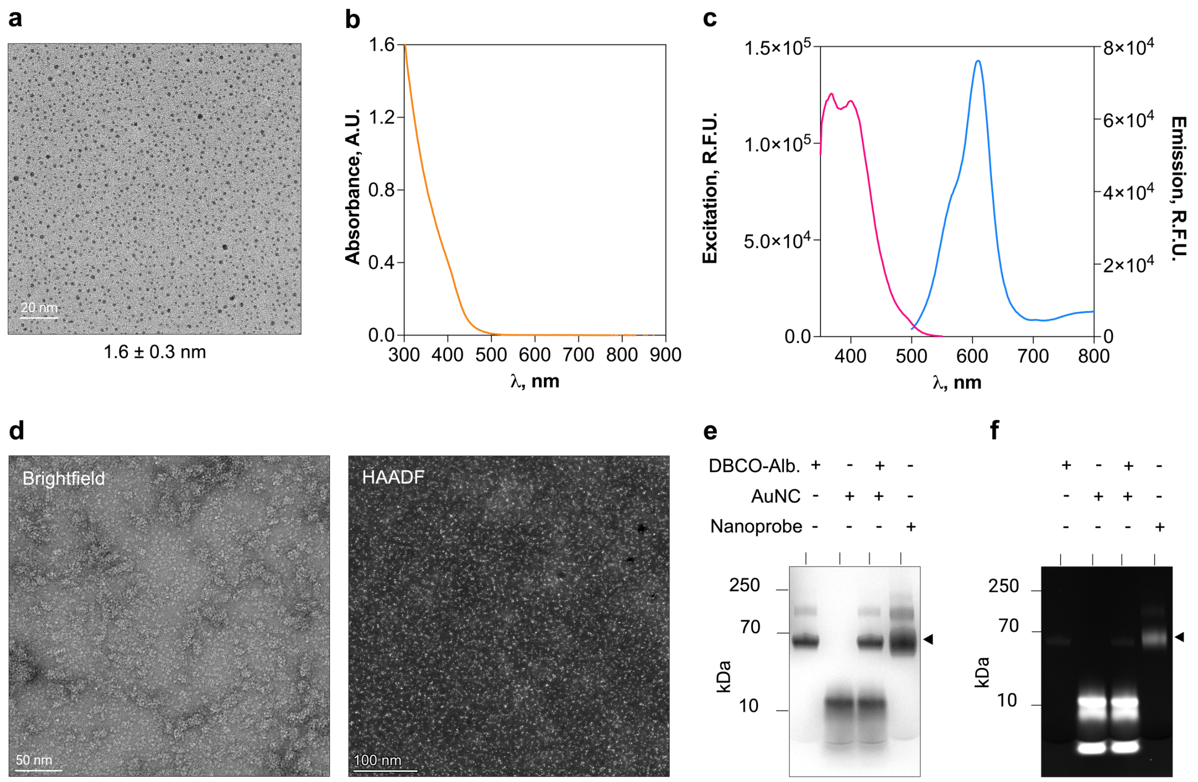

Supplementary Fig. 24. Assembly of non-cleavable, control nanoprobe. a. TEM images of AuNC with -GGGGGG- peptide sequence (N = 200 particles, mean ± s.d.). UV-Vis absorbance (**b**) and excitation and emission (**c**) spectra of AuNC. Different peptide sequences did not significantly alter the size distribution and UV-Vis and fluorescence emission of AuNC. TEM image of non-cleavable nanoprobe in (**d**) brightfield and HAADF mode. Gel electrophoresis of non-cleavable nanoprobe stained with (**e**) Coomassie and (**f**) luminol. The arrows correspond to the nanoprobe bands. Scale bars = 20, 50, or 100 nm, as specified.

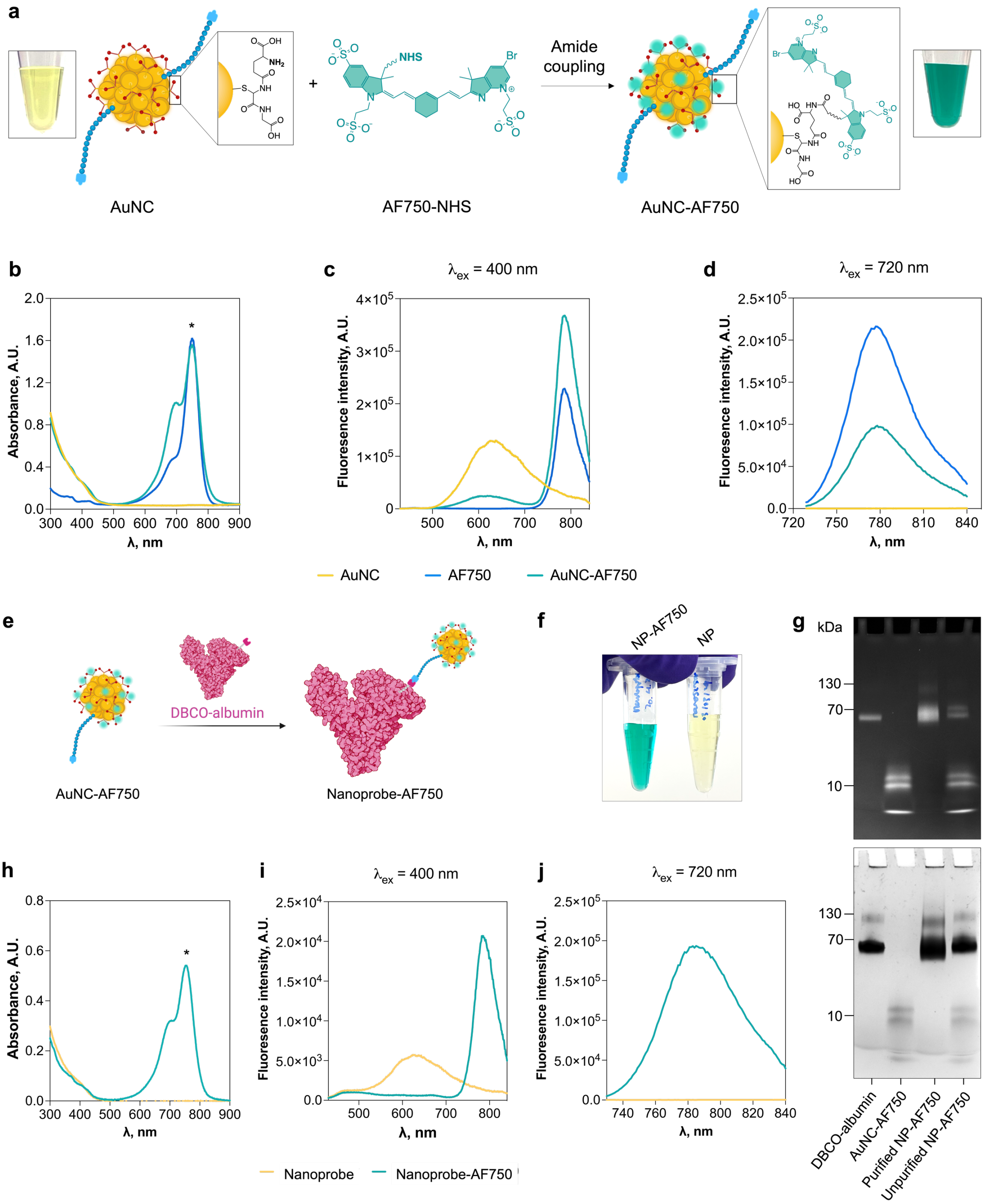

Supplementary Fig. 25. Preparation of AF750-labelled ALBANC nanoprobe. The first step involves functionalization of AuNC with AF750. **a**. GSH in AuNC can react with AF750-NHS ester *via* amide coupling reactions. Note the photographs of AuNC versus AuNC-AF750 suspension, showing color change post-functionalization. **b**. Absorbance spectra of AuNC, AF750, and AF750-labelled AuNC. Asterisk indicates the new absorbance of AuNC-AF750 corresponding to the attached AF750 dyes. Fluorescence emission of AuNC, AuNC-AF750, and AF750 excited at (**c**) 400 nm and (**d**) 720 nm. **e**. Assembly of AF750-labelled AuNC with DBCO-albumin. **f**. Representative image of unlabelled nanoprobe (NP) and AF750-labelled NP suspension. The color green arose from rhodamine in AuNC. **g**. Gel electrophoresis stained with luminol (top) and Coomassie (bottom) showing successful assembly of AF750-

labelled nanoprobe. Absorbance (**h**) and fluorescence emission of rhodamine-labelled nanoprobe and unlabelled nanoprobe excited at (**i**) 400 nm and (**j**) 720 nm.

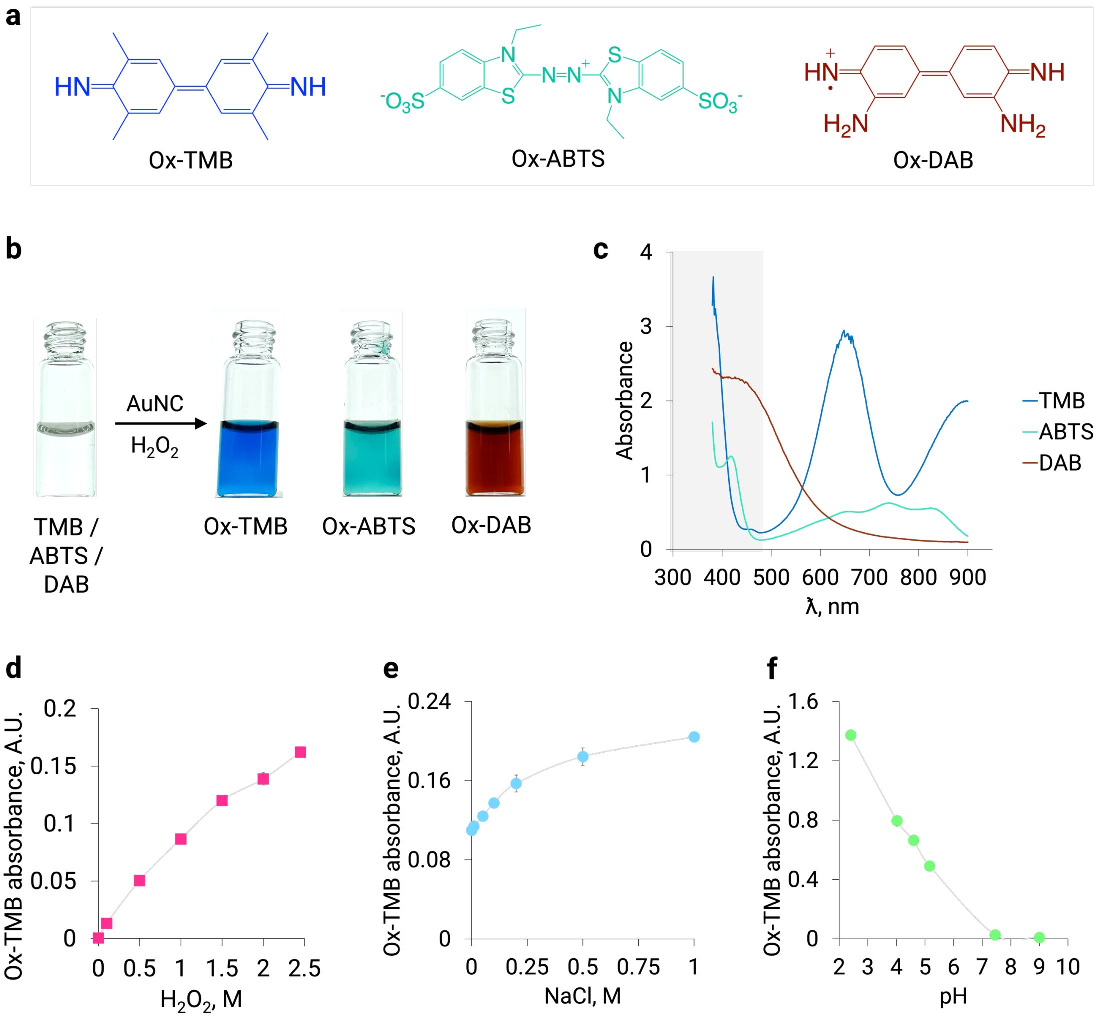

Supplementary Fig. 26. Optimization of peroxidase assay for AuNC detection. **a.** Chemical structures of peroxidase substrates after peroxidase assay. **b**. Photograph of peroxidase solution containing AuNC before and after the assay. Peroxidase-mimicking activity of AuNC converted the substrates into their oxidised, colored products. **c**. The corresponding UV-Vis spectra showing absorbance correlated to oxidation of peroxidase substrates. Catalytic activity of AuNC as a function of (**d**) hydrogen peroxide, (**e**) salt content, and (**f**) solution pH. Experiment was repeated independently 3 times with similar results.

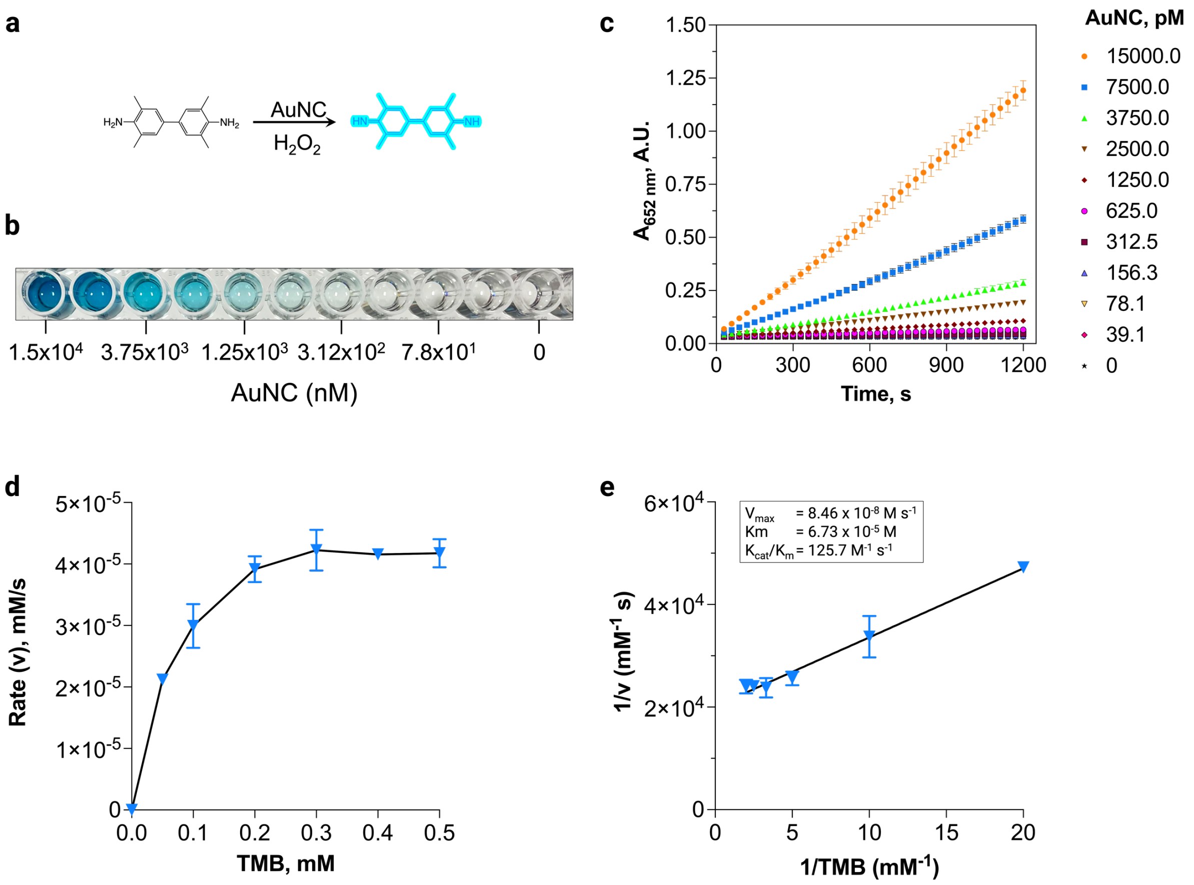

Supplementary Fig. 27. Sensitivity and kinetics of AuNC detection using peroxidase assay. **a.** Schematic illustration of the peroxidase assay using TMB. **b**. Representative photograph of 96-well plates containing different concentrations of AuNC after 15min peroxidase assay. **c**. Evolution of A_{652nm} in the presence of different concentrations of AuNC in synthetic urine (in nanomolar) over 20 min of assay. Kinetic measurement of the peroxidase activity of AuNC, by plotting the absorbance of oxidised TMB (λ = 652 nm, or $A_{652\ nm}$) over 20 min. **d.** Steady-state kinetic (*v*) of peroxidase activity of AuNC in converting TMB into oxidized TMB (*N* = 3, mean ± s.d.). Experiment was performed in 10 mM sodium acetate buffer pH 4.0 with with 10 μM AuNC at room temperature over 2.5 minutes, where H_2O_2 concentration was kept constant at 2 M. **e**. Lineweaver-Burk plot of kinetics of peroxidase activity of AuNC (*N* = 3, mean ± s.d), from which kinetic paramaters (V_{max}, K_m, K_{cat}/K_m) are extracted.

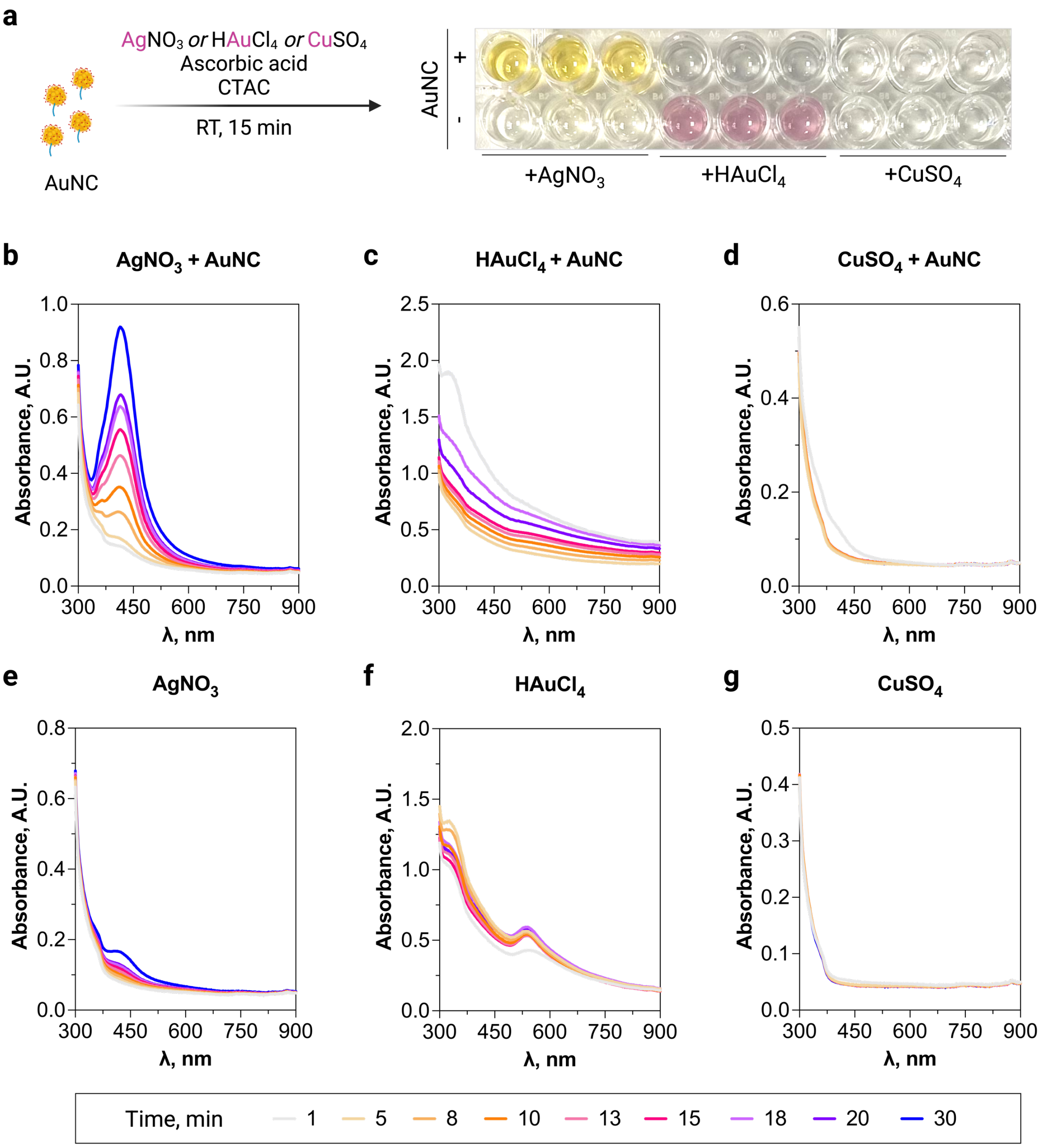

Supplementary Fig. 28. **Exploring the ability of AuNC to form metallic nanoparticles. a**. Visual representation of color change (or lack thereof) of AuNC suspension after addition of different metal salts in the presence of CTAC and ascorbic acid. Plot of absorbance of AuNC suspension with ascorbic acid and CTAC after addition of $AgNO_3$ (**b**), $HAuCl_4$ (**c**), and $CuSO_4$ (**d**). Plot of absorbance of ascorbic acid and CTAC solution (without AuNC) after addition of $AgNO_3$ (**e**), $HAuCl_4$ (**f**), and $CuSO_4$ (**g**). Experiments were repeated independently 3 times with similar results.

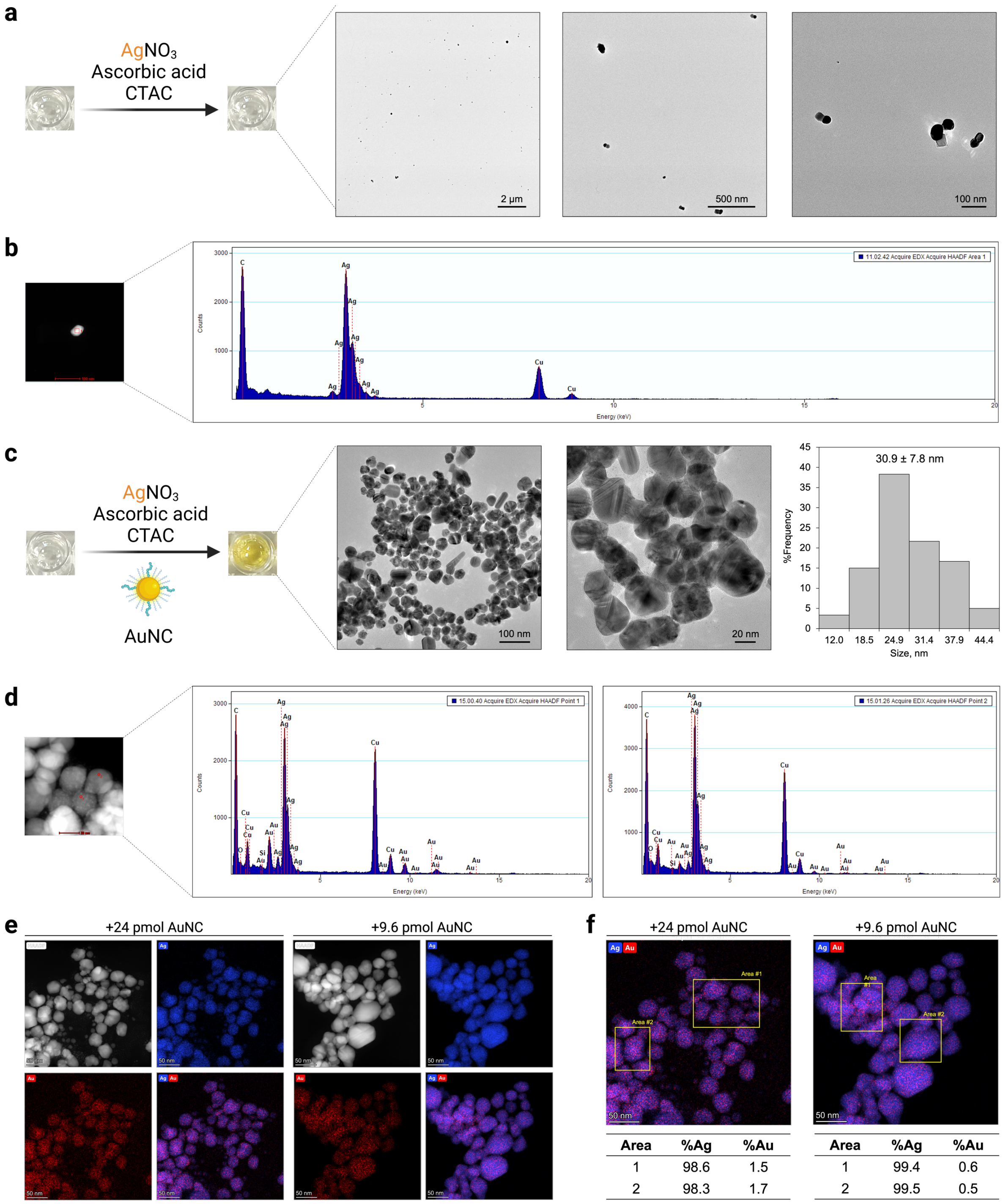

+24 pmol AuNC

Area	%Ag	%Au
1	98.6	1.5
2	98.3	1.7

+9.6 pmol AuNC

Area	%Ag	%Au
1	99.4	0.6
2	99.5	0.5

Supplementary Fig. 29. Characterization of AuNC-Ag alloy formation assay. **a**. Representation of alloy formation in the absence of AuNC, whereby negligible color change is detectable. Without AuNC, very little AgNP, that gives rise to 'background signal', is formed as shown by TEM. **b**. EDS spectrum confirming the presence of Ag in AgNP (and the absence of Au). **c**. Representation of alloy formation in the presence of AuNC, whereby orange color change is detectable. This color change is caused by the formation of AuNC-Ag alloy nanoparticles, as shown by TEM. **d**. EDS spectrum confirming the presence of Au and Ag in AuNC-Ag alloy nanoparticles. **e.** Elemental map photographs of the formed AuNC-Ag alloy prepared using two different concentrations of AuNC, with the corresponding elemental content measured by EDS shown in (**f**). The higher AuNC present in the alloy formation assay, the higher Au content in the formed AuNC-Ag alloy.

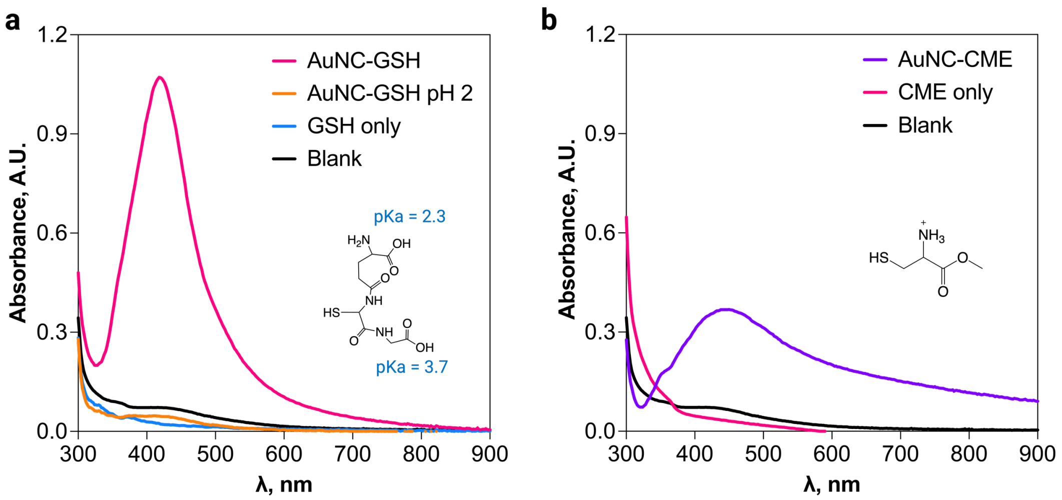

Supplementary Fig. 30. GSH is important for AuNC-Ag alloy to form. UV-Vis absorbance of alloy formation assay with (**a**) glutathione (GSH)-functionalized AuNC and (**b**) cysteine methyl ester (CME)-functionalized AuNC. AuNC-GSH could form AuNC-Ag alloy at pH 6. At pH 2 in which GSH is more protonated, the alloy formation is suppressed, likely indicating that the carboxylate form of GSH is important for AuNC-Ag to form. Contrastingly, AuNC-CME that possessed positive charge formed alloy less effectively. This suggests that AuNC-Ag formation is mediated by the complexation between carboxylates in GSH with Ag ions.

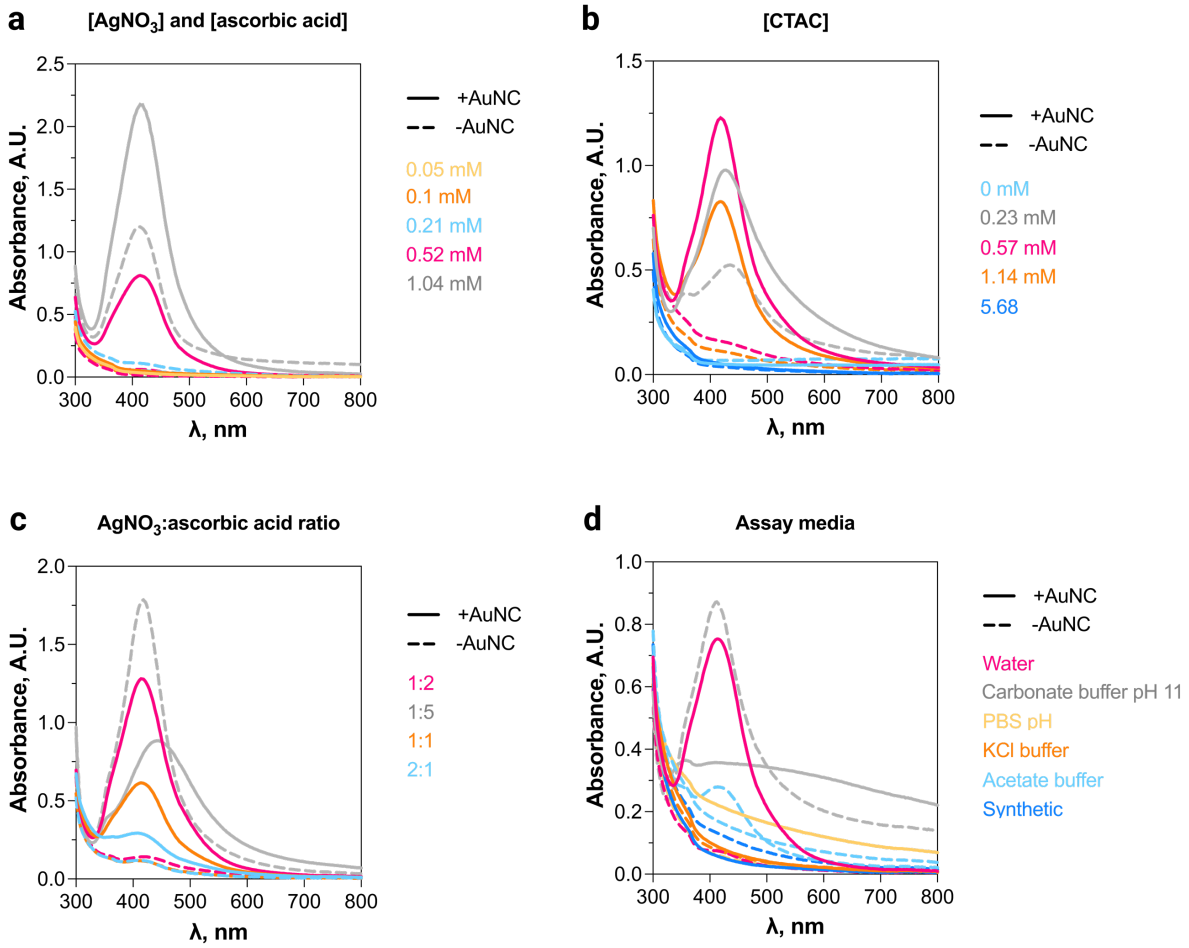

Supplementary Fig. 31. Optimization of alloy formation assay using silver nitrate. Formation of AuNC-Ag alloy (absorbance peak at λ = 414 nm) as a function of (**a**) $AgNO_3$ and ascorbic acid concentration, (**b**) CTAC concentration, (**c**) molar ratio of $AgNO_3$ to ascorbic acid and (**d**) assay media. Conditions that produced the most AuNC-Ag alloy formation with minimum background signal (coming from the formation of silver nanoparticles (AgNP)) are in pink. Solid lines represent assay with AuNC, dashed lines represent assay without AuNC (control). Experiment was repeated independently 3 times with similar results.

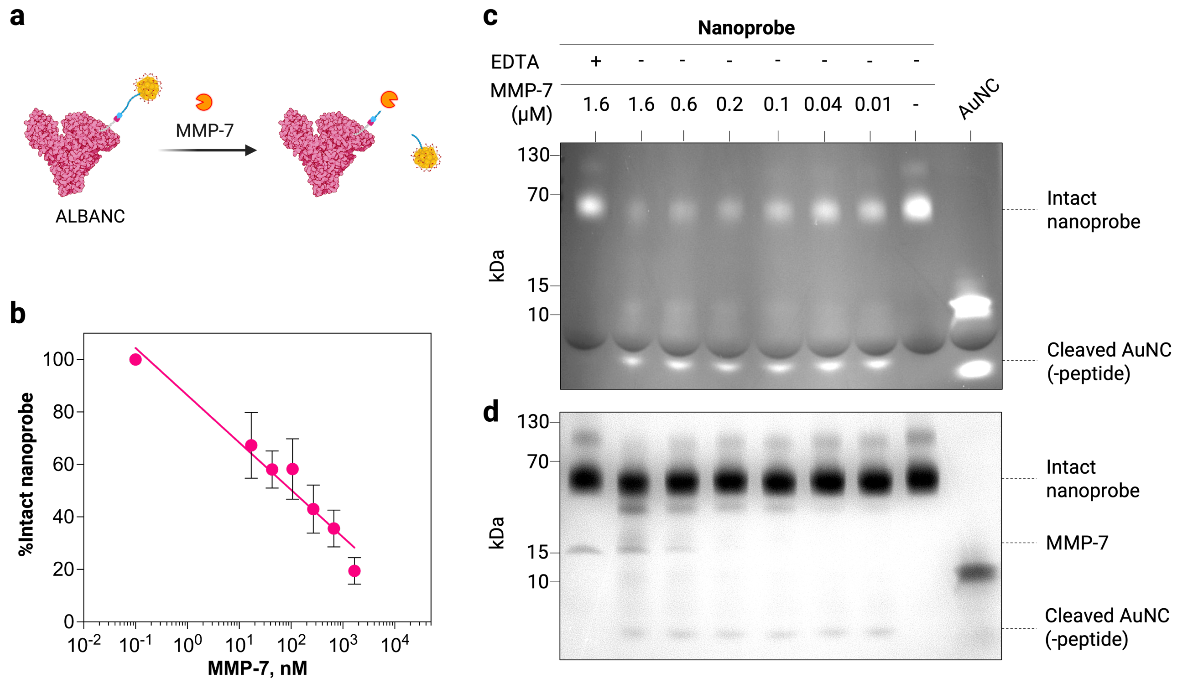

Supplementary Fig. 32. Nanoprobes could be cleaved by recombinant MMP-7. a. Schematic illustration of cleavage of ALBANC by recombinant MMP-7. **b.** Plot of %intact ALBANC after incubation with varying concentration of recombinant MMP-7. %Intact nanoprobe decreased with increasing concentration of MMP-7 (mean ± s.d., N = 3 independent experiments, unpaired t-test). Representative PAGE of reaction mixture of ALBANC nanoprobe, stained with (**c**) luminol and (**d**) Coomassie after incubation with varying concentrations of MMP-7.

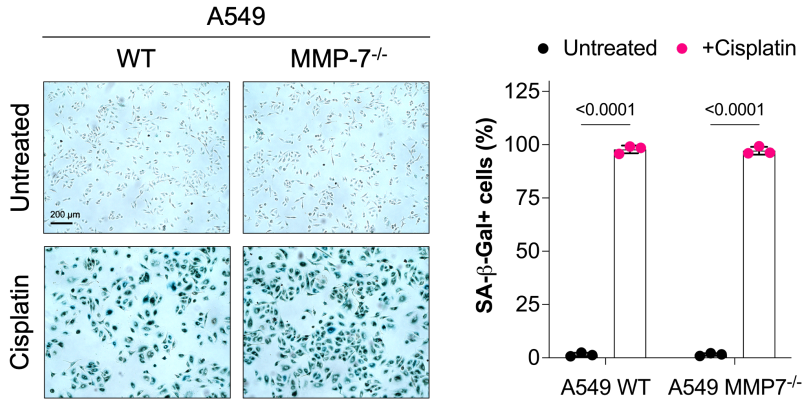

Supplementary Fig. 33. Representative images of untreated and cisplatin-treated wild-type (WT) and MMP-7$^{-/-}$ A549 cells fixed and stained for SA-β-gal activity. Scale bar = 200 μm (*N* = 3, mean ± s.d.; unpaired t-test).

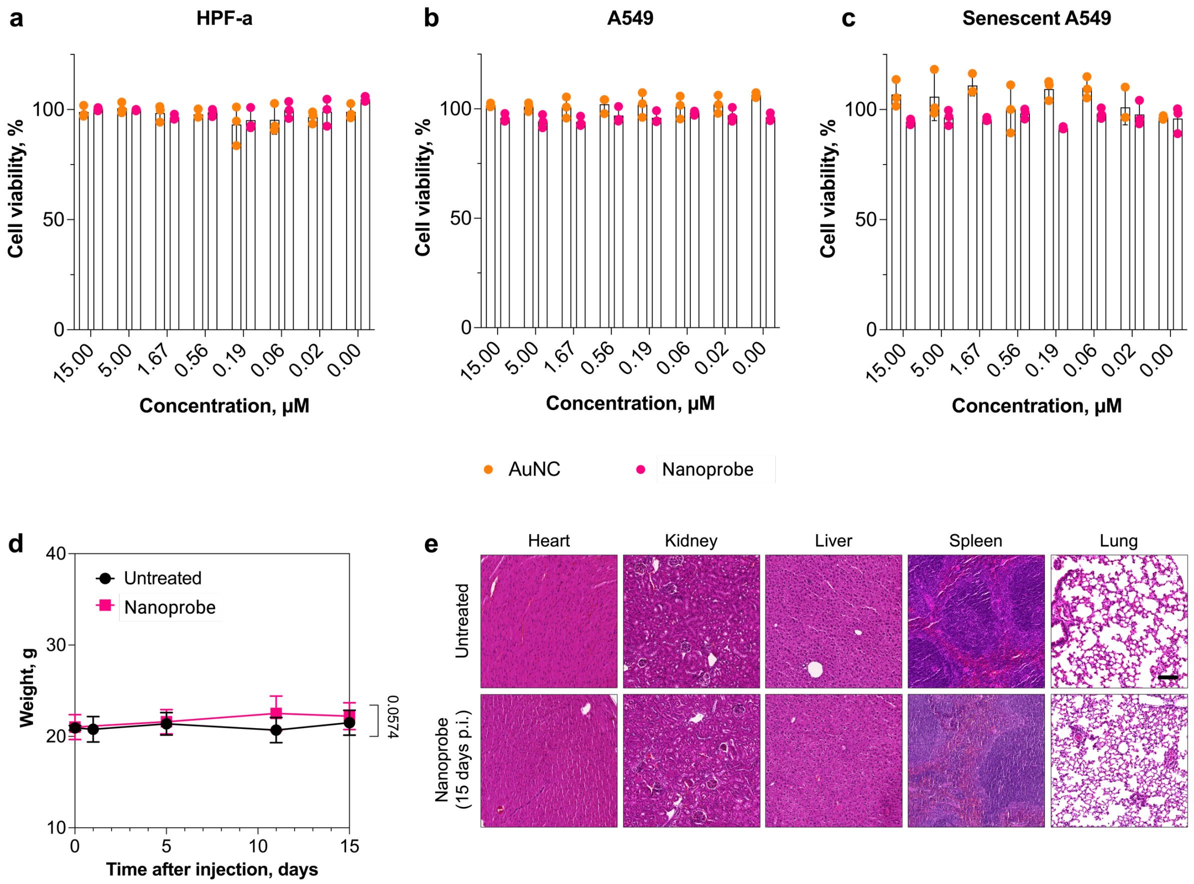

Supplementary Fig. 34. Nanoprobes did not show any toxic effects *in vitro* and *in vivo*. Both AuNC (orange) and ALBANC nanoprobes (pink) exhibited no significant changes in cell viability when incubated with (**a**) HPF-a, (**b**) A549, and (**c**) chemotherapy-induced senescent A549 cells over a 72-hour period in cell culture (mean ± s.e.m., N = 3 independent experiments). **d**. The body weight of C57BL/6 mice, either untreated or intravenously injected with ALBANC nanoprobes (3 nmol [AuNC]) (mean ± s.e.m., N = 3 mice per group). **e** Representative H&E staining of major organs from untreated and nanoprobe-treated mice. Scale bar = 200 μm.

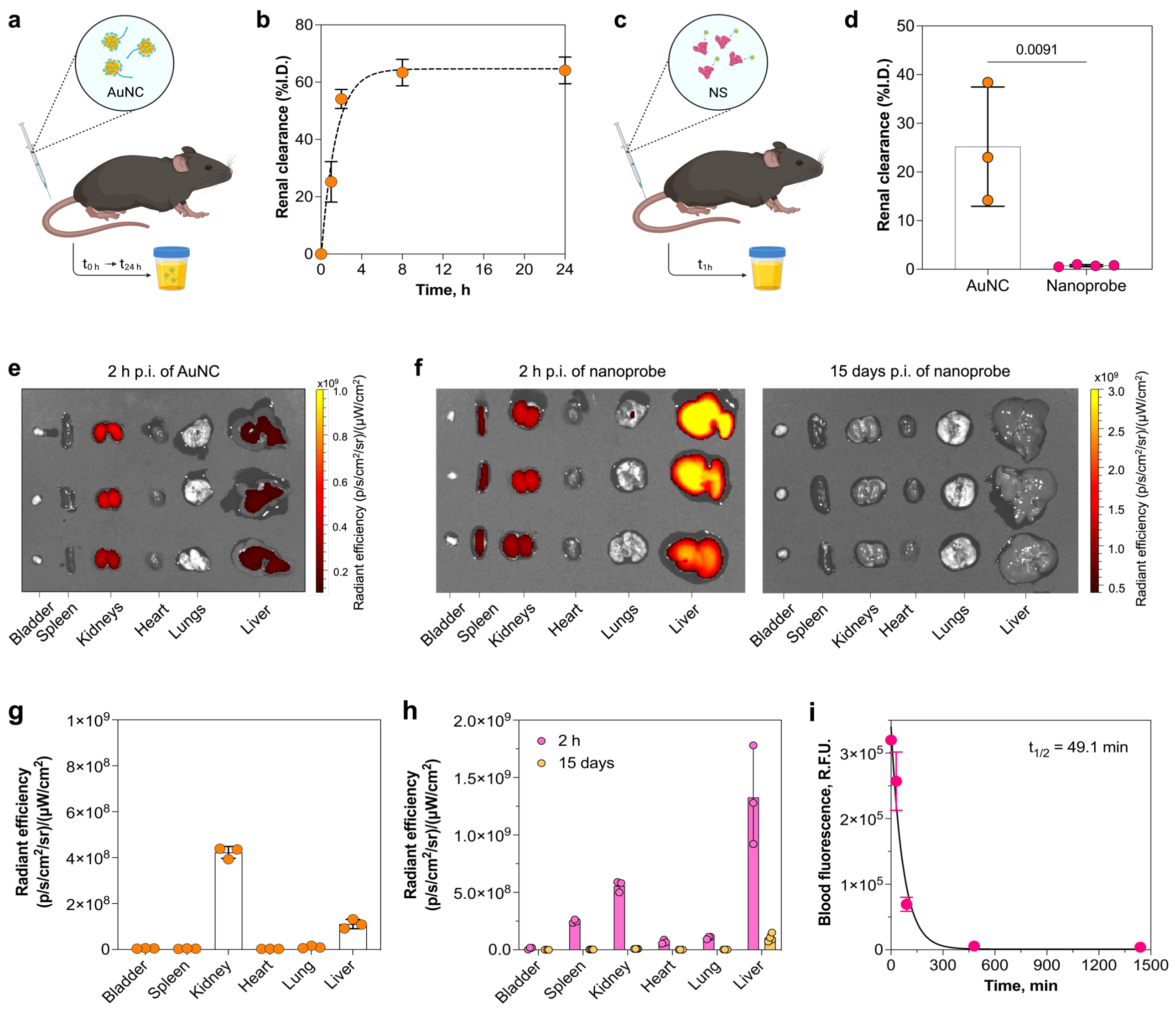

Supplementary Fig. 35. Renal clearance, biodistribution, and pharmacokinetics of AuNC and nanoprobes. a. Schematic illustration of renal clearance study, where AuNCs were intravenously (i.v). injected into C57BL/6 mice, and urine was collected within 24 h post-injection (p.i.). Renally cleared AuNC content in the urine was measured using ICP–MS, plotted in (**b**) as % injected dose (I.D.) (N = 3 mice, mean ± s.e.m.). **c**. Schematic illustration of renal clearance study, where nanoprobes were i.v. injected into C57BL/6 mice, and urine was collected within 2 h p.i. Urine was analyzed using ICP–MS for Au content, plotted in (**d**, N = 4 mice for nanoprobes, mean ± s.e.m, unpaired t test). Organs were harvested at 2 h and 15 days p.i. IVIS images of main organs of mice 2 h p.i. of AuNC (**e**) and of mice 2 h and 15 days p.i. of nanoprobes (**f**), with corresponding fluorescence emission quantifications for each organ plotted in (**g**) for AuNC and (**h**) for nanoprobes (N = 3 mice for AuNC and N = 4 mice for nanoprobes, mean ± s.e.m., ordinary one-way ANOVA and two-way ANOVA, respectively). **i**. Pharmacokinetic study of NIR-labelled nanoprobes in C57BL/6 mice. The serum concentration of nanoprobe was fit to a non-linear fit (N = 4 mice, mean ± s.e.m).

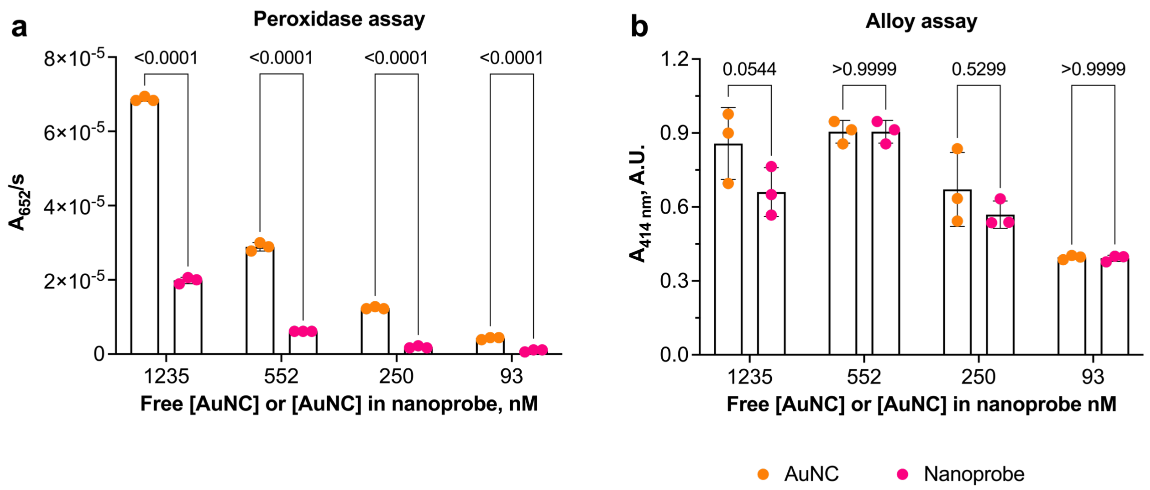

Supplementary Fig. 36. Colorimetric signals from free AuNC versus nanoprobe. a. Peroxidase assay and **b**. Alloy assay for different concentrations of AuNC and nanoprobes (based on [AuNC] content). Data is presented as mean ± s.d.; N = 3 independent experiments; two-way ANOVA with multiple comparisons.

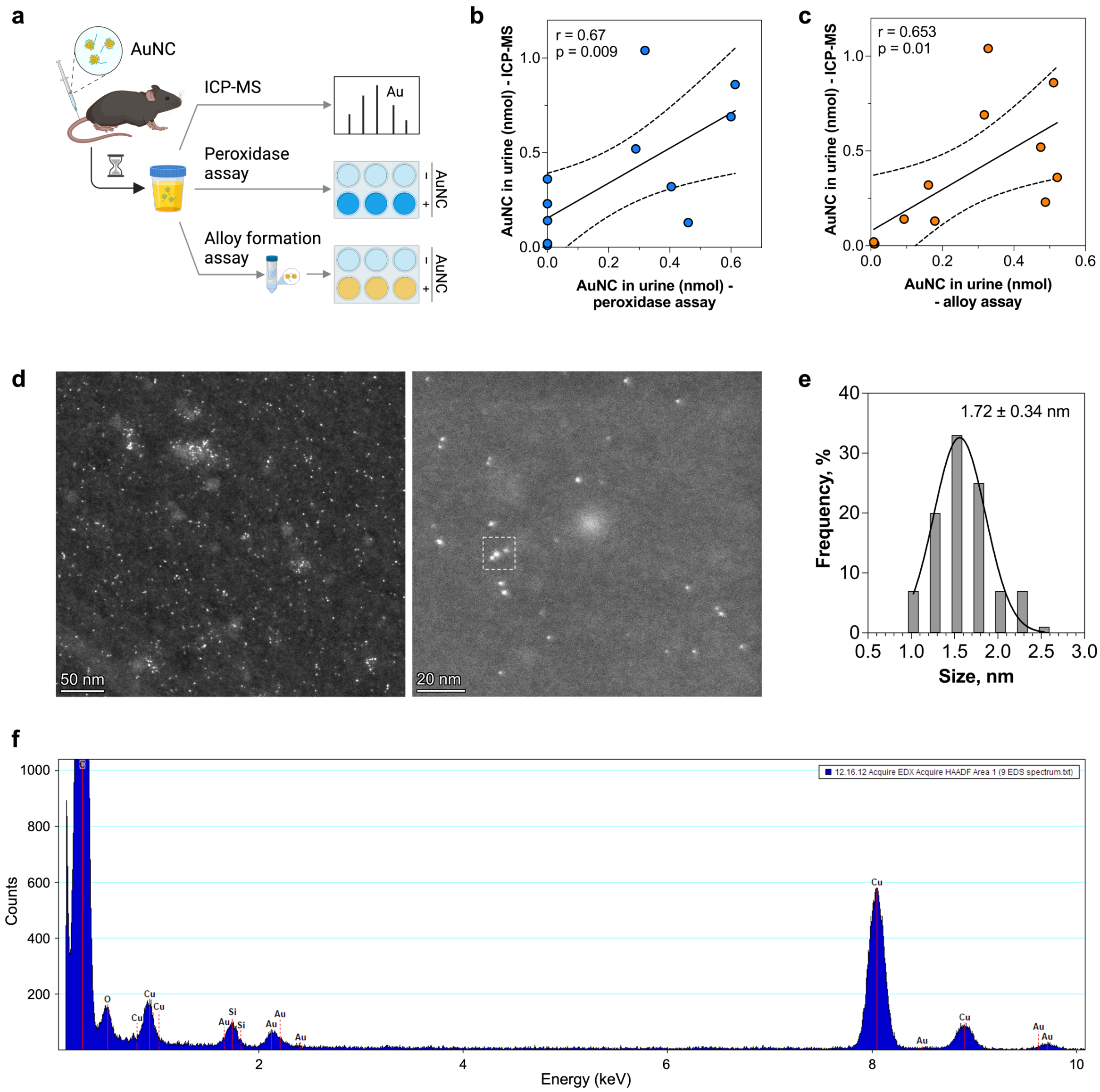

Supplementary Fig. 37. Colorimetric assays enabled sensitive detection of renally cleared AuNC in the urine. a. Schematic of methods to detect the renally cleared AuNCs from mice. ICP-MS, peroxidase assay, and alloy formation assays were performed on the same urine samples to compare their performance in detecting AuNC. Correlation between the estimated AuNC contents measured by (**b**) peroxidase assay and (**c**) alloy formation assay versus total Au content measured by ICP-MS. **d**. TEM images of renally cleared AuNCs and (**e**) their corresponding size distribution (N = 200 particles). **f**. EDX spectra of the urine samples (white box area in (**d**)). Three urine samples were imaged with similar results. Scale bar = 20 or 50 nm as specified.

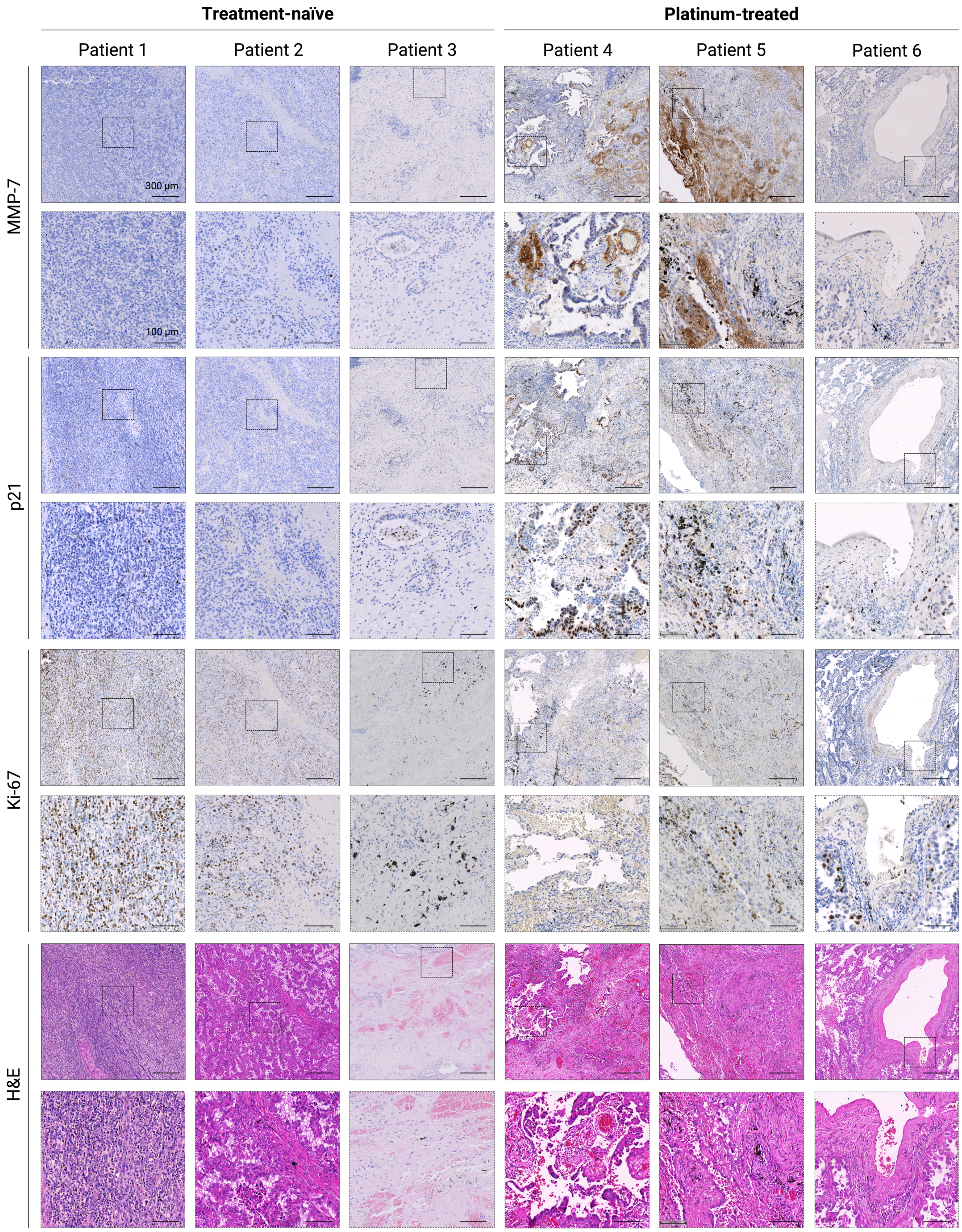

Supplementary Fig. 38. Platinum-treated tumor specimens from non-small cell lung (NSCLC) patients showed a high expression of senescence markers and MMP-7. Representative histological images of NSCLC specimens (consecutive sections) obtained from patients who have not undergone treatment or have received platinum-based therapy, stained for MMP-7, p21, Ki-67 and H&E. Scale bar = 300 µm or 100 µm, as indicated. The square indicates the area that is zoomed in and shown in detail below. Neoadjuvant platinum-based therapy triggers senescence in individuals with NSCLC, as shown by a higher

expression of p21 and lower expression of Ki-67, which is associated with elevated MMP-7 levels in neighboring cells.

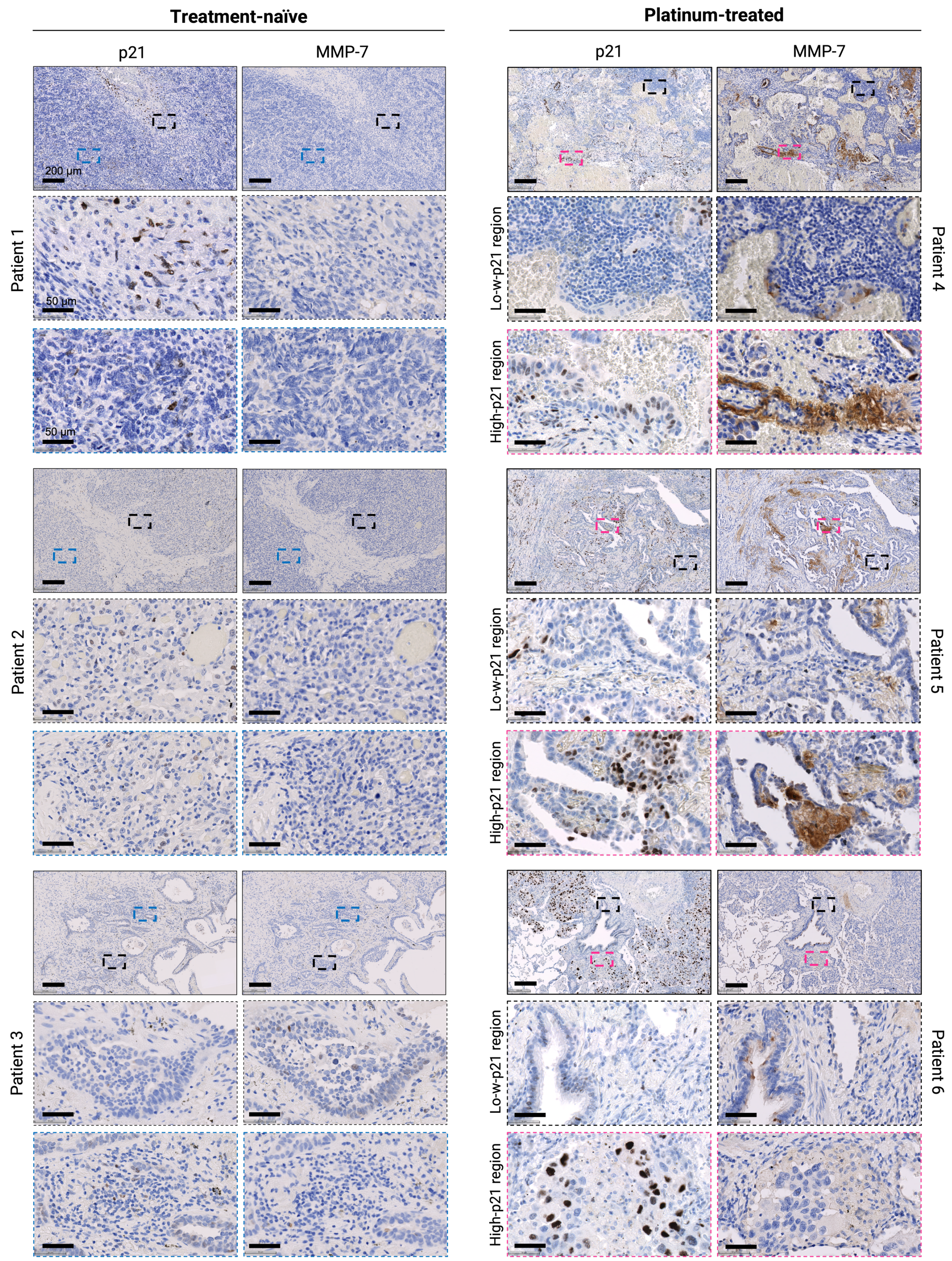

Supplementary Fig. 39. **Platinum-treated tumor specimens showed a higher expression of MMP-7 and p21 compared to treatment-naive tumor specimens.** Representative histological images of NSCLC biopsy samples from treatment-naïve patients and platinum-chemotherapy, analyzed for p21 and MMP-7. Scale bars = 200 µm (top row) or 50 µm (bottom rows). The square indicates the area that is zoomed in and shown in detail below. For platinum-treated specimens, areas with a high expression of p21 showed an elevated MMP-

7 expression in the surrounding area, whereas areas with low p21 expression showed negligible MMP-7 expression. In contrast, treatment-naive specimens showed negligible staining for both p21 and MMP-7.

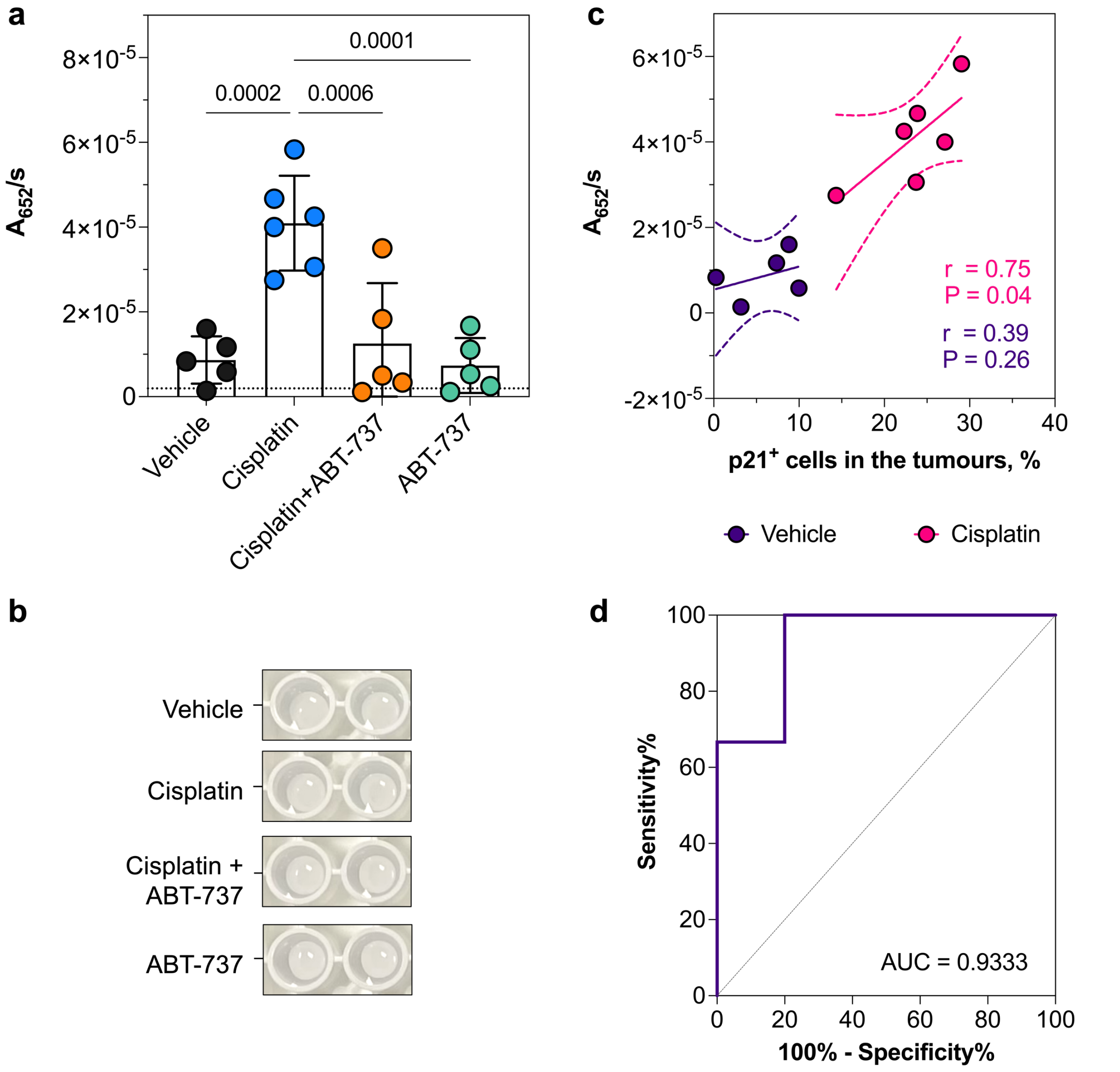

Supplementary Fig. 40. Peroxidase assay enabled spectroscopic, urinary detection of chemotherapy-induced senescence in lung cancer. a. Initial kinetics (A_{652nm}/s) from peroxidase assays of urine samples collected from xenograft mice 2 h p.i. with nanoprobes (n ≥ 5 mice per group, mean ± s.e.m, ordinary one-way ANOVA with Dunnett's multiple comparison test). **b**. Photograph of peroxidase assay on urine samples from xenograft mice injected with nanoprobes. **c**. Correlation analysis between the percentage of MMP-7 in the tumors and in the serum from the xenograft mice, either cisplatin-treated or vehicle-treated group, and intensity of signals of the urine samples detected from peroxidase assay, A_{652nm}/s. Dashed lines represent the 95% confidence interval; dots represent individual mouse. **d**. ROC analysis showing the diagnostic specificity and sensitivity of nanoprobes and peroxidase assay in detecting senescence between cisplatin-treated and vehicle-treated mice groups (AUC = 0.9333, 95% CI = 0.7787 – 1). r = Pearson's coefficient.

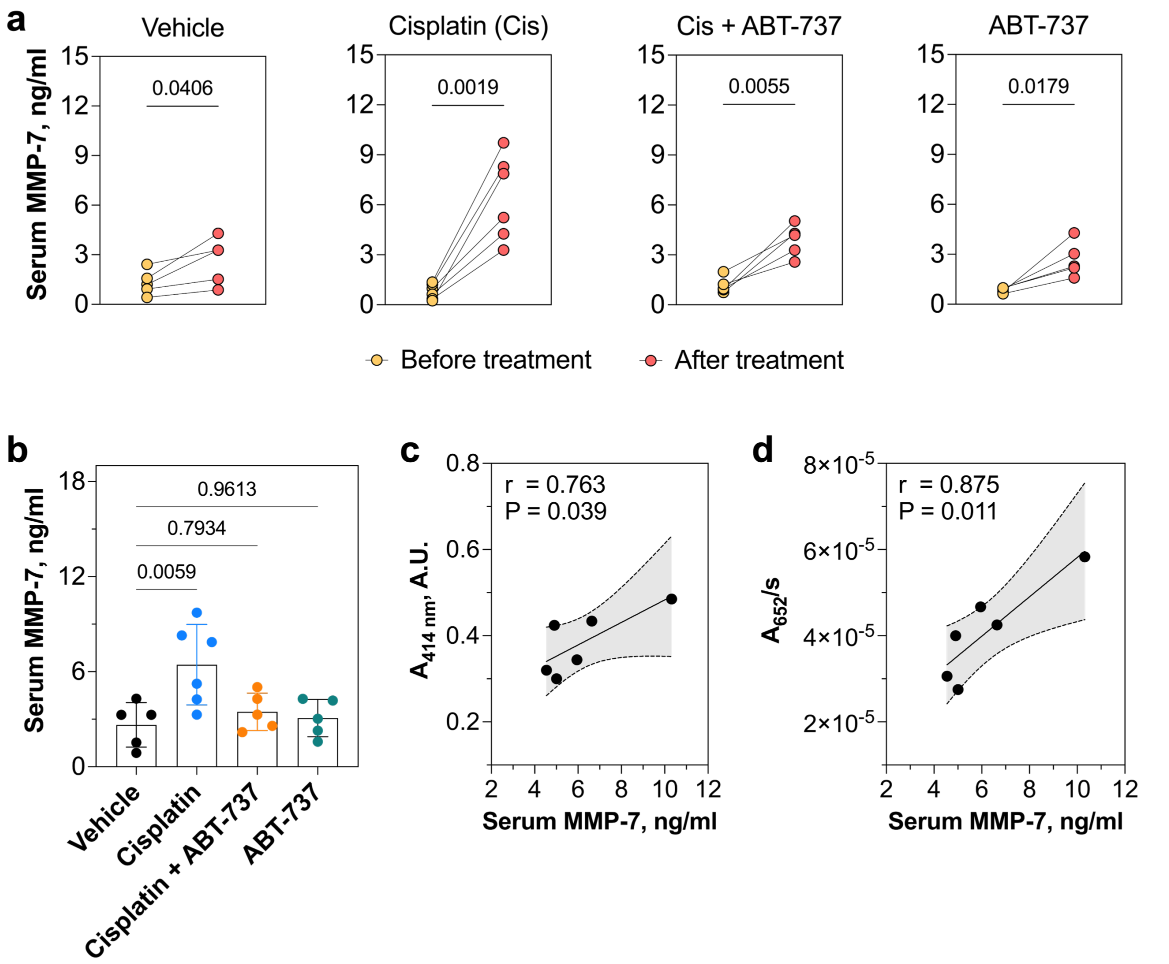

Supplementary Fig. 41. Urinary signal correlates positively with circulating MMP-7 level in the serum. a. Serum concentration of MMP-7, before and after treatment (paired t-test). **b**. Serum concentration of MMP-7 after treatment (mean ± s.d.; ordinary one-way ANOVA with multiple comparisons). Correlation analysis between serum MMP-7 concentration and intensity of signals of the urine samples detected from (**c**) alloy assay and (**d**) peroxidase assay. Dashed lines represent the 95% confidence interval; dots represent individual mouse.

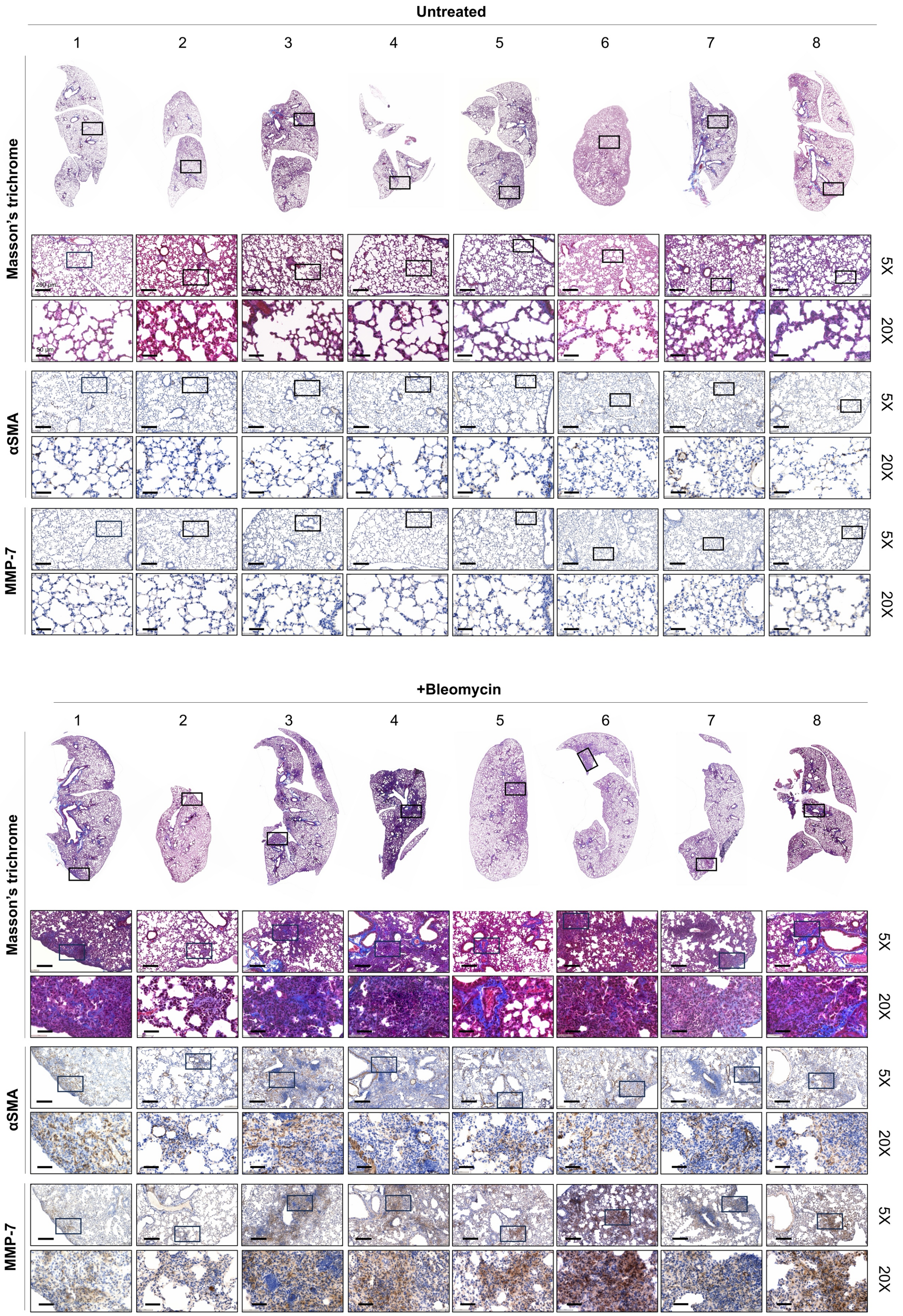

Supplementary Fig. 42. Histological validation of pulmonary fibrosis in bleomycin-treated mice. Lungs from either untreated or bleomycin-treated mice (at the endpoint), stained

for fibrosis markers (Masson's trichrome and *α*-SMA) and MMP-7 expression. Scale bar = 200 or 50 μm as specified (N = 8 mice per group). The square indicates the area that is zoomed in and shown in detail below**.**

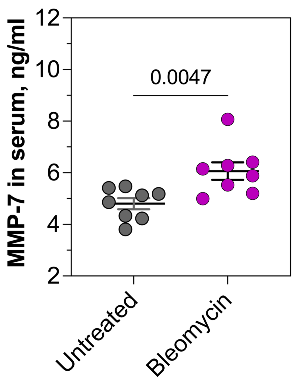

Supplementary Fig. 43. Serum concentration of MMP-7 in untreated mice vs mice after bleomycin treatment (10 days). Data is presented as mean ± s.d.; unpaired t-test.

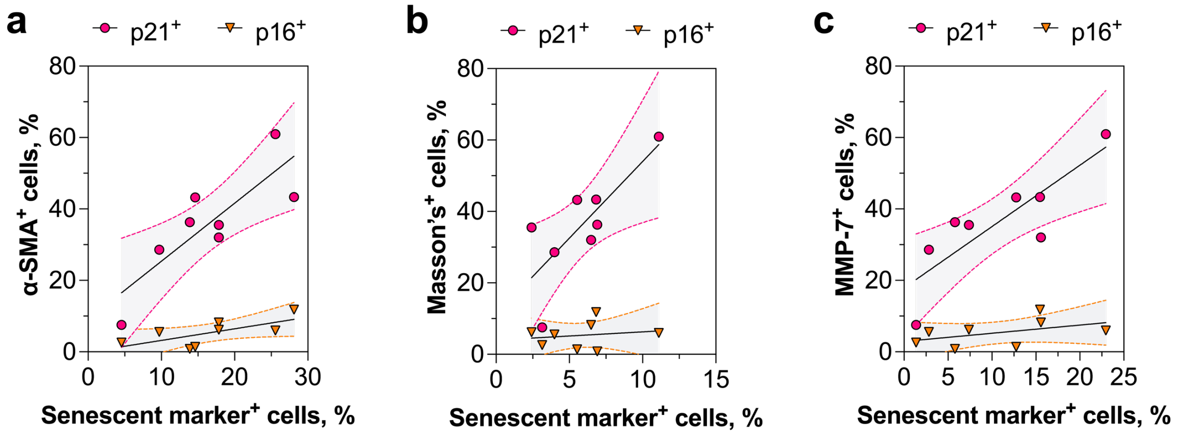

Supplementary Fig. 44. The levels of fibrotic marker (Masson's) and MMP-7 correlated positively with the levels of senescence marker in the lungs of fibrotic mice. Correlation analysis between the percentage of senescence marker, $p21^+$ and $p16^+$, cells with the percentage of α-SMA+ cells (**a**), Masson's$^+$ (**b**) and MMP-7^+ cells in the lungs. Dashed lines represent the 95% confidence interval; dots represent individual mouse. Pearson's coefficient (r) and P-values: (**a**) r=0.8292, P=0.0109 for p21; r=0.6846, P=0.0611 for p16; (**b**) r=0.7767, P=0.0234 for p21; r=0.1719, P=0.6840 for p16; (**c**) r=0.8393, P=0.0092 for p21; r=0.4611, P=0.2501 for p16.

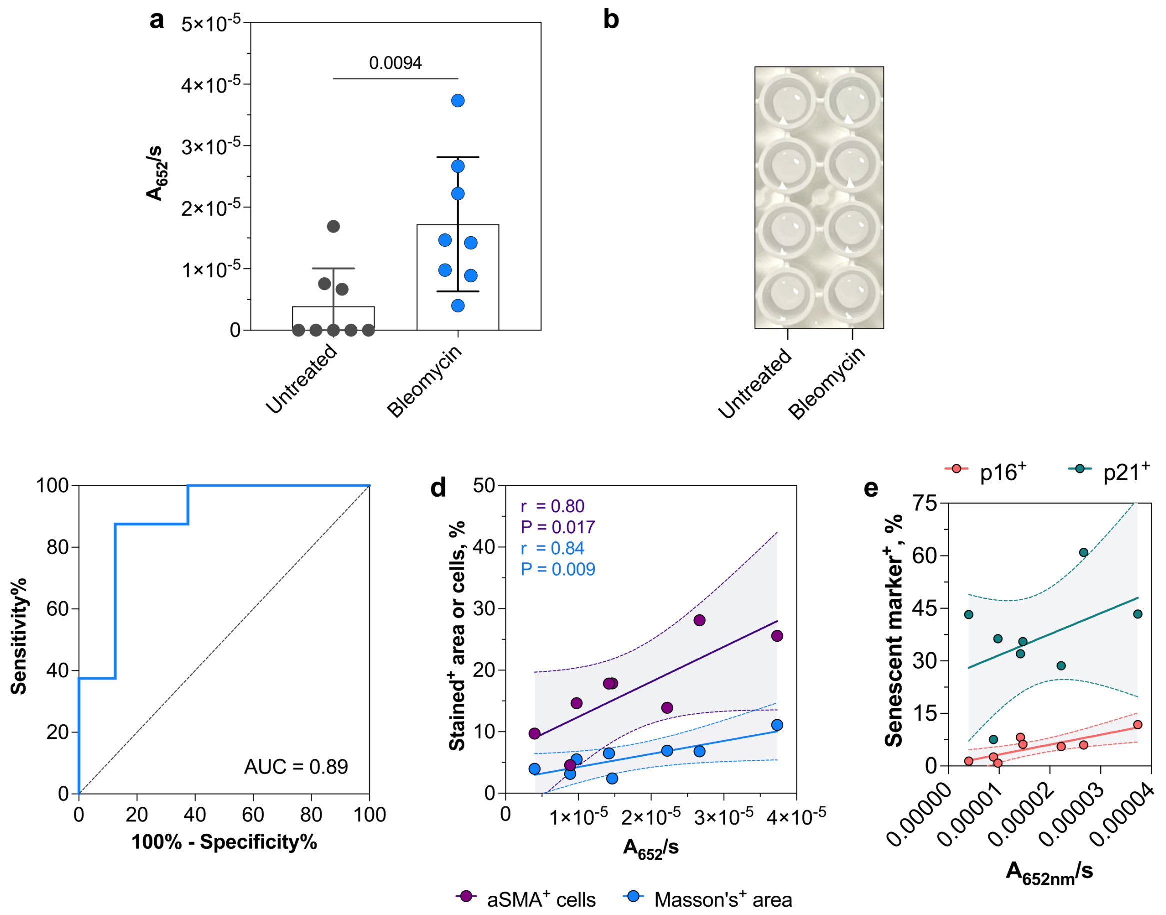

Supplementary Fig. 45. Peroxidase assay enabled spectroscopic, urinary detection of pulmonary fibrosis. a. Initial kinetic values (A_{652nm}/s) from peroxidase assay of urine samples collected from untreated or bleomycin-treated mice 2 h p.i. with nanoprobes (N = 8 mice per group, mean ± s.e.m, unpaired two-tailed t tests). **b**. Photograph of the peroxidase assay on urine samples from untreated or bleomycin-treated mice injected with nanoprobes. **c**. ROC analysis showing the diagnostic specificity and sensitivity of nanoprobes and alloy formation assay in detecting fibrosis between bleomycin-treated and untreated mice groups (AUC = 0.89, 95% CI = 0.7215 – 1). **d**. Correlation analysis between the percentage of Masson's^{+} area and α-SMA^{+} cells in the lungs and intensity of signals of the urine samples detected from peroxidase assay. Dashed lines represent the 95% confidence interval; dots represent individual mouse. **e**. Correlation analysis between the percentage of senescence marker, p21^{+} and p16^{+}, cells with the urinary signal from peroxidase assay. Dashed lines represent the 95% confidence interval; dots represent individual mouse. Pearson's coefficient (r) and P-values: r=0.4308, P=0.2866 for p21; r=0.8374, P=0.0095 for p16.

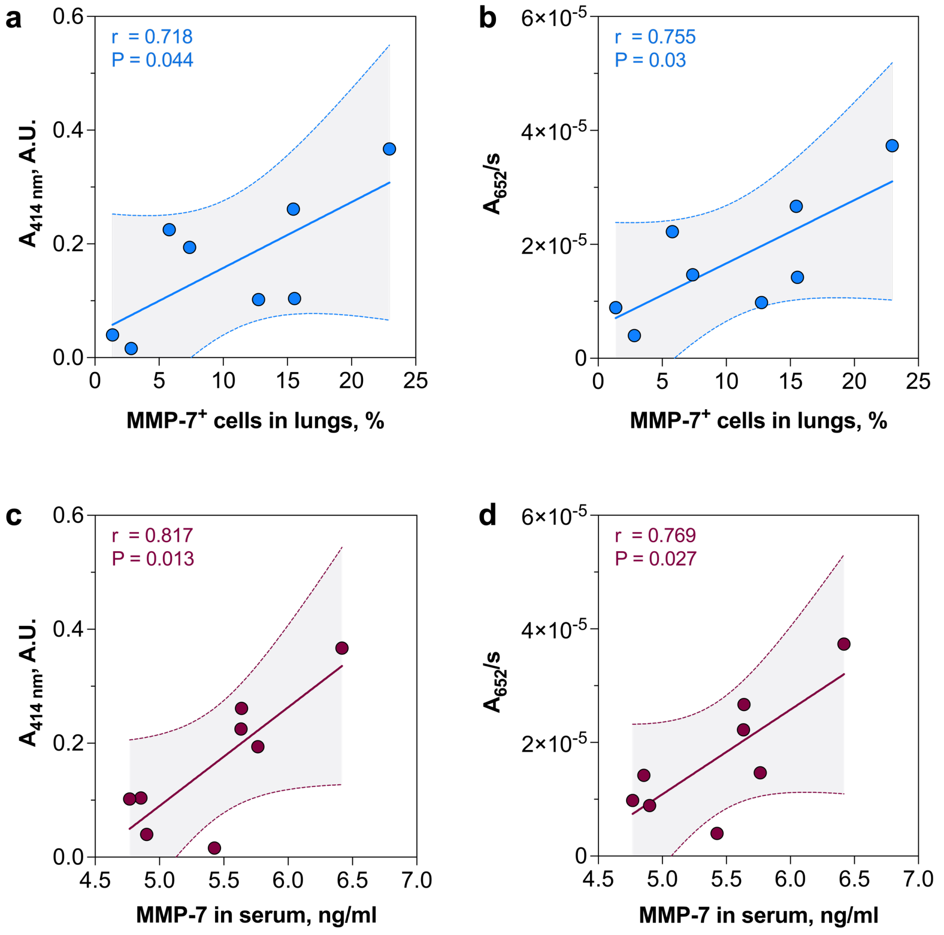

Supplementary Fig. 46. Urinary signals correlated positively with MMP-7 levels in the lungs and serum of fibrotic mice. Correlation analysis between the percentage of MMP-7$^+$ cells in the lungs (**a, b**) and MMP-7 in the serum (**c, d**) and intensity of signals of the urine samples detected from alloy formation assay (**a, c**) and peroxidase assay (**b, d**). Dashed lines represent the 95% confidence interval; dots represent individual mouse. r = Pearson's coefficient.

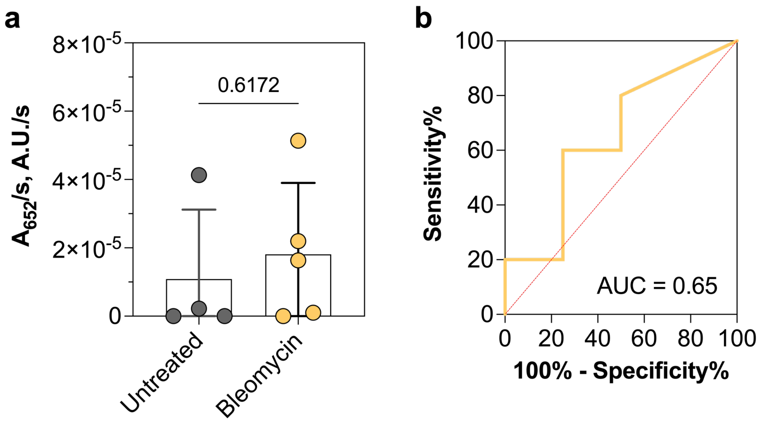

Supplementary Fig. 47. Peroxidase assay for early/incipient fibrosis experiment. a. Absorbance rate values ($A_{625\ nm}$/s) from peroxidase assay of urine samples collected from untreated or bleomycin-treated (7 days) mice 2 h p.i. with nanoprobes (*N* = 4 or 5 mice per group, mean ± s.e.m, unpaired two-tailed t tests). **b**. ROC analysis showing the diagnostic specificity and sensitivity of nanoprobes and alloy formation assay in detecting incipient fibrosis between bleomycin-treated (7 days) and untreated mice groups (AUC = 0.65, 95% CI = 0.265 – 1).

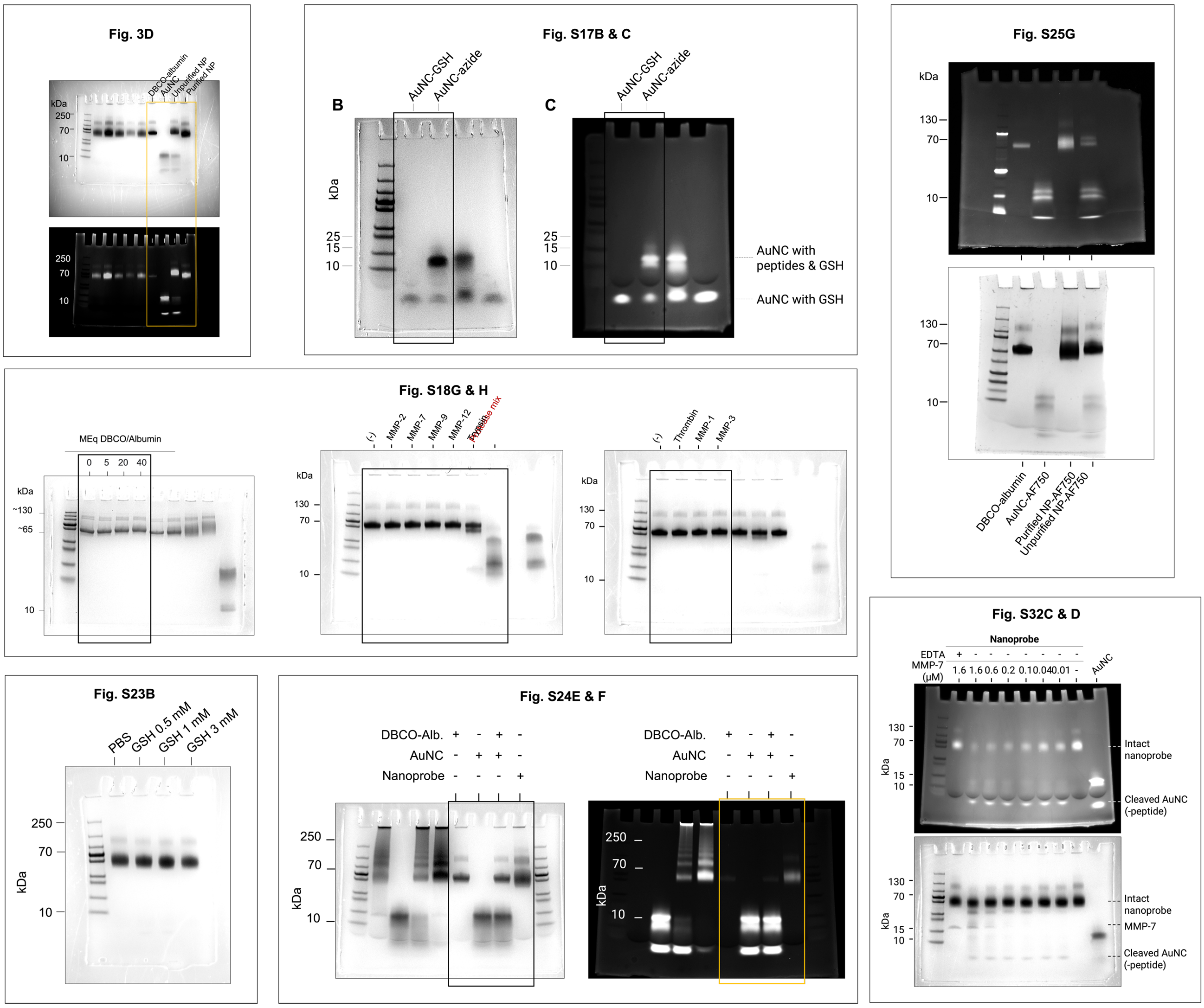

Supplementary Fig. 48. Uncropped polyacrylamide gel electrophoresis (PAGE) blots.

Supplementary Table 1. Patient data.

Patient no.	Pathology	Stage	Neoadjuvant treatment
1	Lung adenocarcinoma (multifocal)	T2a N2 M0	None
2	Lung adenocarcinoma (multifocal)	T1c N0 M0	None
3	Lung adenocarcinoma (multifocal)	T4 N1 M0	None
4	Lung adenocarcinoma (multifocal)	T3 N3 M0	Carboplatin/pemetrexed (5 cycles)
5	Lung adenocarcinoma (70% acinar & 30% micropapillary growth patterns)	T3 N0 M0	Cisplatin/vinorelbine (4 cycles)
6	Lung adenocarcinoma (30% lepidic component)	T3 N2 M0	Platinum-based chemotherapy

Supplementary Table 2. List of primary antibodies used for immunohistochemistry.

Antibody	Host species	Provider	Dilution
Anti-pRb	Rabbit	Cell signaling (D20B12)	1:1000
Anti-p21	Rabbit	Abcam (ab109520)	1:2000
Anti-MMP-7 (Human)	Rabbit	Abcam (ab207299)	1:8000
Anti-Ki-67	Rabbit	Cell Signaling (12202, D3B5)	1:1000
Anti-p16	Rabbit	Proteintech (10883-1-AP)	1:1000
Anti-αSMA	Rabbit	Cell signaling (D4K9N)	1:1000
Anti-MMP-7 (Mouse)	Rabbit	Abcam (ab302893)	1:1000

Supplementary Table 3. Parameters used for RT-qPCR.

Cycle step	Temperature, °C	Time, s	Number of cycles
Initial denaturation	95	60	1
Denaturation	95	15	40
Extension	60	30	40
Melt curve	72	300	1

Supplementary Table 4. Sequences of oligonucleotide primers employed in the amplification of target genes during RT-qPCR.

Target gene	Forward, 5'-3'	Reverse, 5'-3'
ACTB	AGAAGGATTCCTATGTGGGC	TACTTCAGGGTGAGGATGC
LMNB1	GTATGAAGAGGAGATTAACGAGAC	TACTCAATTTGACGCCCAG
CDKN1A	CAGCATGACAGATTTCTACC	CAGGGTATGTACATGAGGAG
GLB1	GACAGTACCAGTTTTCTGAG	ATAGACTCTTTCTCTAGCAGC
MMP7	GCCAGATGTTGCAGAATACTC	TATGATACGATCCTGTAGGTGAC

Supplementary Table 5. List of antibodies used for western blot. 1° = primary, 2° = secondary.

Antibody	Host species	Provider	Dilution
Anti-*β*-actin (1°)	Rabbit	Proteintech (20536-1-AP)	1:1000
Anti-pRb (1°)	Rabbit	Cell signaling (D20B12)	1:10,000
Anti-p21 (1°)	Rabbit	Abcam (ab109520)	1:1000
Anti-MMP-7 (1°)	Rabbit	Abcam (ab207299)	1:1000
HRP-conjugated AffiniPure Anti-Rabbit IgG (H+L) (2°)	Donkey	Jackson ImmunoResearch (711-035-152)	1:2000
