# Supplementary material for: Urinary detection of therapy-induced senescence and fibrosis using an injectable albumin-based nanoprobe
Source: Nat Aging. 2026 May 13;6(5):1158–76. doi: 10.1038/s43587-026-01116-z (PMC13190281; doi:10.1038/s43587-026-01116-z)
Supplement: Supplementary file 2 — Reporting Summary [file 43587_2026_1116_MOESM2_ESM.pdf]

nature portfolio

Corresponding author(s): Daniel Muñoz-Espín & Ljiljana Fruk

Last updated by author(s): 22/12/2025

Reporting Summary

Nature Portfolio wishes to improve the reproducibility of the work that we publish. This form provides structure for consistency and transparency in reporting. For further information on Nature Portfolio policies, see our Editorial Policies and the Editorial Policy Checklist.

Statistics

For all statistical analyses, confirm that the following items are present in the figure legend, table legend, main text, or Methods section.

n/a	Confirmed	
☐	☒	The exact sample size (*n*) for each experimental group/condition, given as a discrete number and unit of measurement
☐	☒	A statement on whether measurements were taken from distinct samples or whether the same sample was measured repeatedly
☐	☒	The statistical test(s) used AND whether they are one- or two-sided<br>*Only common tests should be described solely by name; describe more complex techniques in the Methods section.*
☐	☒	A description of all covariates tested
☐	☒	A description of any assumptions or corrections, such as tests of normality and adjustment for multiple comparisons
☐	☒	A full description of the statistical parameters including central tendency (e.g. means) or other basic estimates (e.g. regression coefficient) AND variation (e.g. standard deviation) or associated estimates of uncertainty (e.g. confidence intervals)
☐	☒	For null hypothesis testing, the test statistic (e.g. *F*, *t*, *r*) with confidence intervals, effect sizes, degrees of freedom and *P* value noted<br>*Give P values as exact values whenever suitable.*
☒	☐	For Bayesian analysis, information on the choice of priors and Markov chain Monte Carlo settings
☒	☐	For hierarchical and complex designs, identification of the appropriate level for tests and full reporting of outcomes
☒	☐	Estimates of effect sizes (e.g. Cohen's *d*, Pearson's *r*), indicating how they were calculated

Our web collection on statistics for biologists contains articles on many of the points above.

Software and code

Policy information about availability of computer code

Data collection	Processed and annotated single-cell RNA sequencing data from Huang et al (PMID: 39729352) was downloaded from https://figshare.com/articles/dataset/scRNA-sequencing_raw_data_of_LUAD/24797265 as stated from the original publication.
Data analysis	A Seurat object compiling all detected cells by Huang et al (PMID: 39729352) was created from the available count and meta data matrices using the CreateSeuratObject function of the Seurat package (v5.3). The epithelial cell cluster was obtained from the complete dataset using the subset function within Seurat (v5.3). Data from epithelial cells were re-processed using the standard Seurat pipeline and integrated using the Canonical Correlation Analysis (CCA) method. Uniform manifold approximation and projection (UMAP) was used to visualise the dataset in a two-dimensional space. Differential expression testing was performed using the FindMarkers function selecting “MAST” as test type. Significant upregulated genes between lung adenocarcinoma patients treated with neoadjuvant chemotherapy (NCT) (n=5) and naïve lung adenocarcinoma patients (Control, n=4) were defined by showing Log2Fold ⍰1 and significant adjusted p-value or false discovery rate (FDR) <0.05. Gene set enrichment analysis was performed using the clusterProfiler R package (v4.14.6) against the Hallmark, the C2 canonical pathway collection (C2.cp.v5.1) and Human SenMayo (PMID: 35974106) gene sets that were downloaded from the Molecular Signatures Database (https://www.gsea-msigdb.org/gsea/msigdb).

For manuscripts utilizing custom algorithms or software that are central to the research but not yet described in published literature, software must be made available to editors and reviewers. We strongly encourage code deposition in a community repository (e.g. GitHub). See the Nature Portfolio guidelines for submitting code & software for further information.

Data

Policy information about availability of data

All manuscripts must include a data availability statement. This statement should provide the following information, where applicable:

- Accession codes, unique identifiers, or web links for publicly available datasets
- A description of any restrictions on data availability
- For clinical datasets or third party data, please ensure that the statement adheres to our policy

All data associated with this study are present in the paper, the Materials and Methods section, or the Supplementary Materials.

Field-specific reporting

Please select the one below that is the best fit for your research. If you are not sure, read the appropriate sections before making your selection.

☒ Life sciences ☐ Behavioural & social sciences ☐ Ecological, evolutionary & environmental sciences

For a reference copy of the document with all sections, see nature.com/documents/nr-reporting-summary-flat.pdf

Life sciences study design

All studies must disclose on these points even when the disclosure is negative.

Sample size	*Describe how sample size was determined, detailing any statistical methods used to predetermine sample size OR if no sample-size calculation was performed, describe how sample sizes were chosen and provide a rationale for why these sample sizes are sufficient.*
Data exclusions	*Describe any data exclusions. If no data were excluded from the analyses, state so OR if data were excluded, describe the exclusions and the rationale behind them, indicating whether exclusion criteria were pre-established.*
Replication	*Describe the measures taken to verify the reproducibility of the experimental findings. If all attempts at replication were successful, confirm this OR if there are any findings that were not replicated or cannot be reproduced, note this and describe why.*
Randomization	*Describe how samples/organisms/participants were allocated into experimental groups. If allocation was not random, describe how covariates were controlled OR if this is not relevant to your study, explain why.*
Blinding	*Describe whether the investigators were blinded to group allocation during data collection and/or analysis. If blinding was not possible, describe why OR explain why blinding was not relevant to your study.*

Reporting for specific materials, systems and methods

We require information from authors about some types of materials, experimental systems and methods used in many studies. Here, indicate whether each material, system or method listed is relevant to your study. If you are not sure if a list item applies to your research, read the appropriate section before selecting a response.

Materials & experimental systems

n/a	Involved in the study	
☐	☒	Antibodies
☐	☒	Eukaryotic cell lines
☐	☐	Palaeontology and archaeology
☐	☒	Animals and other organisms
☐	☒	Human research participants
☒	☐	Clinical data
☐	☐	Dual use research of concern

Methods

n/a	Involved in the study	
☒	☐	ChIP-seq
☒	☐	Flow cytometry
☒	☐	MRI-based neuroimaging

Antibodies

Antibodies used

Antibody, Host Species, Manufacturer, Catalogue Number, Clone (where applicable)
Phosphorylated Rb (pRb), Rabbit, Cell signalling, D20B12
p21, Rabbit, Abcam, ab109520 and ab302893
MMP-7, Rabbit, Abcam, ab207299
Ki-67, Rabbit, Cell Signaling, 12202, D3B5
p16, Rabbit, Proteintech, 10883-1-AP
αSMA, Rabbit, Cell signaling, D4K9N
β-actin, Rabbit, Proteintech, 20536-1-AP

	HRP-conjugated AffiniPure Anti-Rabbit IgG (H+L), Donkey, Jackson ImmunoResearch, 711-035-152
Validation	Antibody, Catalogue Number, Validation<br>Phosphorylated Rb (pRb), D20B12, validated by IHC, WB, IF; referenced by >552 publications.<br>p21, ab109520 and ab302893, validated by IHC, WB, IF; referenced by >530 publications.<br>MMP-7, ab207299, validated by IHC, WB, IF; referenced by >23 publications.<br>Ki-67, 12202, validated by IHC; referenced by >320 publications.<br>p16, 10883-1-AP, validated by WB, IHC, IF/ICC, FC (Intra), IP, ELISA referenced by >480 publications.<br>αSMA, D4K9N, validated by IHC, WB, IF; referenced by >850 publications.<br>β-actin, 20536-1-AP, validated by IHC, WB, IF; referenced by >3968 publications.<br>HRP-conjugated AffiniPure Anti-Rabbit IgG (H+L), 711-035-152, validated by IHC, WB, ELISA; referenced by >1328 publications.

Eukaryotic cell lines

Policy information about cell lines

Cell line source(s)	A549: ATCC (Catalog. no. CCL-185).<br>SK-Mel-103: Sigma Aldrich (Catalog. no. SCC439).<br>MDA-MB-231: ATCC (Catalog. no. HTB-26).<br>PC-3: ATTC (Catalog. no. CRL-1435)<br>L1475(luc): generated from KrasG12D/WT;p53Fx/Fx mice.<br>HPF-a: ScienCell research laboratories (Catalog. no. 3310).
Authentication	A549 and HPF-a cells were authenticated by STR profiling by the Cancer Research UK Cambridge Institute. L1475(luc) cell line was validated as described in Turrell et al., 2017.
Mycoplasma contamination	All cell lines were routinely tested for mycoplasma infection by Universal Mycoplasma Detection Kit (ATCC, 30-1012K).
Commonly misidentified lines<br>(See ICLAC register)	No commonly misidentified lines were used in the study.

Palaeontology and Archaeology

Specimen provenance	*Provide provenance information for specimens and describe permits that were obtained for the work (including the name of the issuing authority, the date of issue, and any identifying information). Permits should encompass collection and, where applicable, export.*
Specimen deposition	*Indicate where the specimens have been deposited to permit free access by other researchers.*
Dating methods	*If new dates are provided, describe how they were obtained (e.g. collection, storage, sample pretreatment and measurement), where they were obtained (i.e. lab name), the calibration program and the protocol for quality assurance OR state that no new dates are provided.*

☐ Tick this box to confirm that the raw and calibrated dates are available in the paper or in Supplementary Information.

Ethics oversight	*Identify the organization(s) that approved or provided guidance on the study protocol, OR state that no ethical approval or guidance was required and explain why not.*

Note that full information on the approval of the study protocol must also be provided in the manuscript.

Animals and other organisms

Policy information about studies involving animals; ARRIVE guidelines recommended for reporting animal research

Laboratory animals	Female athymic nude mice (Crl:NU(NCr)-Foxn1nu, 11 weeks) were used for MMP-7 validation and A549 xenograft tumour studies. Female C57BL/6 mice (11–12 weeks old) were used for renal clearance, toxicity, and pharmacokinetic, and lung fibrosis studies. Female C57BL/6 mice, either 2 months or 19 months old, were used to compare expression of MMP-7 in young and aged mice. CC57BL/6 mice (12.3-week-old) were used for 'healthy tissues with cisplatin mice experiments'. Female C57BL/6J mice were used for L1475(luc) orthotopic mice experiments. Complete descriptions can be found in relevant figure legends and Methods.
Wild animals	The study did not involve wild animals.
Field-collected samples	The study did not involve field-collected samples.
Ethics oversight	All mice protocols were approved for Ethical Conduct by the Home Office England and Central Biomedical Services (CBS) of the University of Cambridge, regulated under the Animals (Scientific Procedures) Act 1986, as stated in the International Guiding Principles for Biomedical Research involving animals, which fully comply with the current Home Office legislation.

Note that full information on the approval of the study protocol must also be provided in the manuscript.

Human research participants

Policy information about studies involving human research participants

Population characteristics	The available patient information is provided in the Supplementary Table 1.
Recruitment	Human biopsies and ethical regulations. Human lung adenocarcinoma samples were collected from the Royal Papworth Hospital Research Tissue Bank (RPHRTB) after being reviewed by the RPHRTB project review committee (Project Number T02722). Lung tissue samples from patients with idiopathic pulmonary fibrosis were collected from the RPHRTB under Project Numbers T02147 and T02259.<br>RPHRTB has a derogation under the UK Human Tissue Authority (HTA) to supply samples (HTA number 12212) that are surplus to therapeutic necessity and were acquired with Research Ethics Committee-approved RPHRTB permission. Patients signed the RPHRTB general consent form, approving the use of their biopsies for research purposes and sample transfer was covered by a valid Material Transfer Agreement. Written consent was obtained for all tissue samples using Papworth Hospital Research Tissue Bank's ethical approval (East of England - Cambridge East Research Ethics Committee). Further clinical information on the adenocarcinoma samples is available in Supplementary Table 1.
Ethics oversight	Human lung adenocarcinoma samples were collected from the Royal Papworth Hospital Research Tissue Bank (RPHRTB) after being reviewed by the RPHRTB project review committee (Project Number T02722). RPHRTB has a derogation under the UK Human Tissue Authority (HTA) to supply samples (HTA number 12212) that are surplus to therapeutic necessity and were acquired with Research Ethics Committee-approved RPHRTB permission. Patients signed the RPHRTB general consent form, approving the use of their biopsies for research purposes and sample transfer was covered by a valid Material Transfer Agreement. Written consent was obtained for all tissue samples using Papworth Hospital Research Tissue Bank's ethical approval (East of England - Cambridge East Research Ethics Committee).

Note that full information on the approval of the study protocol must also be provided in the manuscript.

Dual use research of concern

Policy information about dual use research of concern

Hazards

Could the accidental, deliberate or reckless misuse of agents or technologies generated in the work, or the application of information presented in the manuscript, pose a threat to:

No	Yes	
☒	☐	Public health
☒	☐	National security
☒	☐	Crops and/or livestock
☒	☐	Ecosystems
☒	☐	Any other significant area

Experiments of concern

Does the work involve any of these experiments of concern:

No	Yes	
☒	☐	Demonstrate how to render a vaccine ineffective
☒	☐	Confer resistance to therapeutically useful antibiotics or antiviral agents
☒	☐	Enhance the virulence of a pathogen or render a nonpathogen virulent
☒	☐	Increase transmissibility of a pathogen
☒	☐	Alter the host range of a pathogen
☒	☐	Enable evasion of diagnostic/detection modalities
☒	☐	Enable the weaponization of a biological agent or toxin
☒	☐	Any other potentially harmful combination of experiments and agents
