# Supplementary figures and images for: Urinary detection of therapy-induced senescence and fibrosis using an injectable albumin-based nanoprobe
Source: Nat Aging. 2026 May 13;6(5):1158–76. doi: 10.1038/s43587-026-01116-z (PMC13190281; doi:10.1038/s43587-026-01116-z)

Fig. 2d

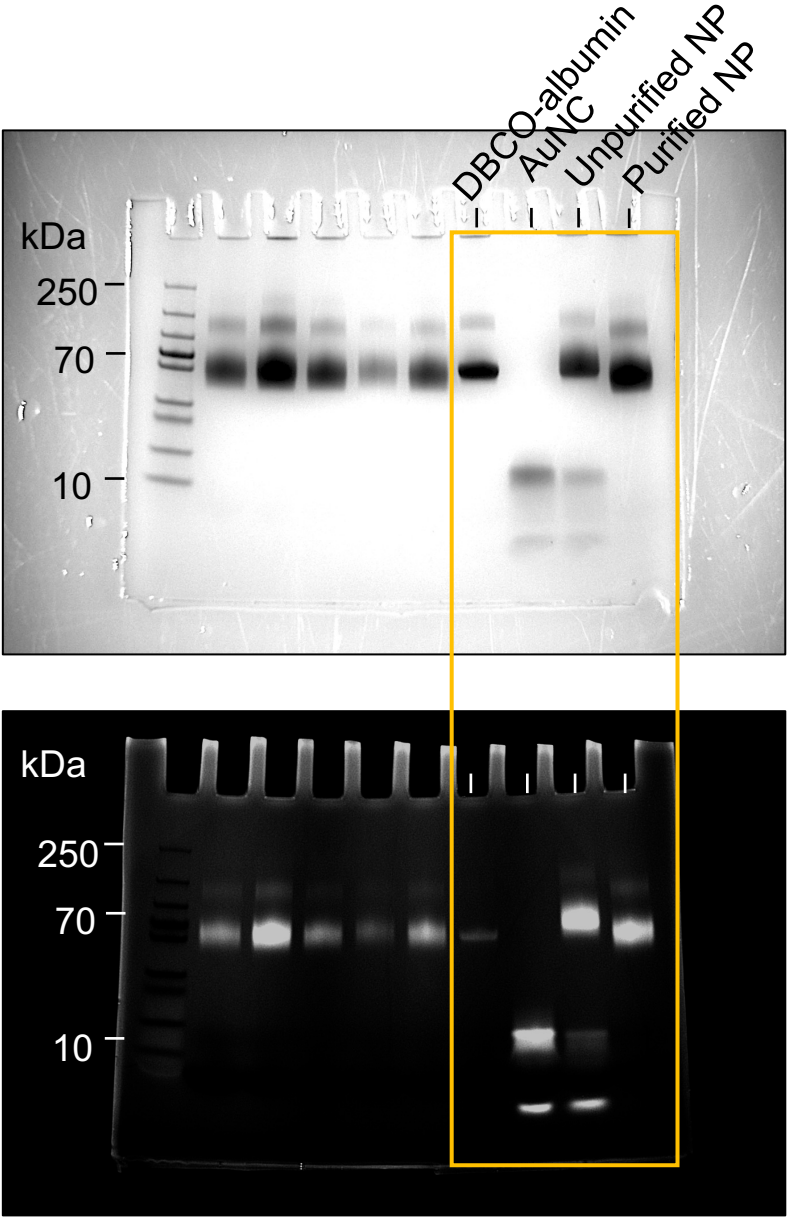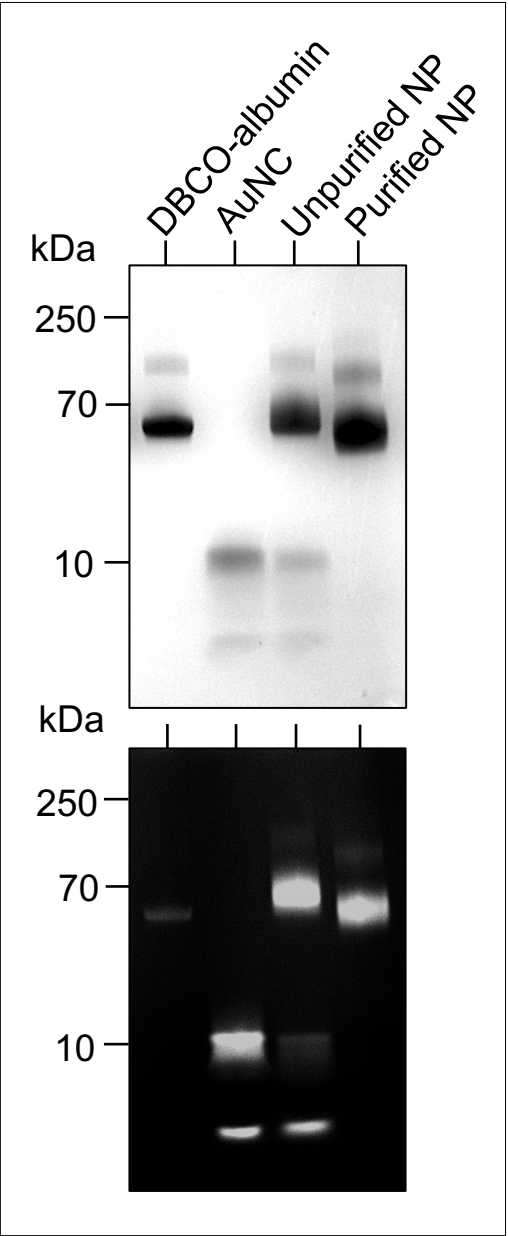

NP = Nanoprobe

Supplement: Supplementary file 6 — PDF file containing original uncropped gels for Fig. 2d. [file 43587_2026_1116_MOESM6_ESM.pdf]

Fig. 3g

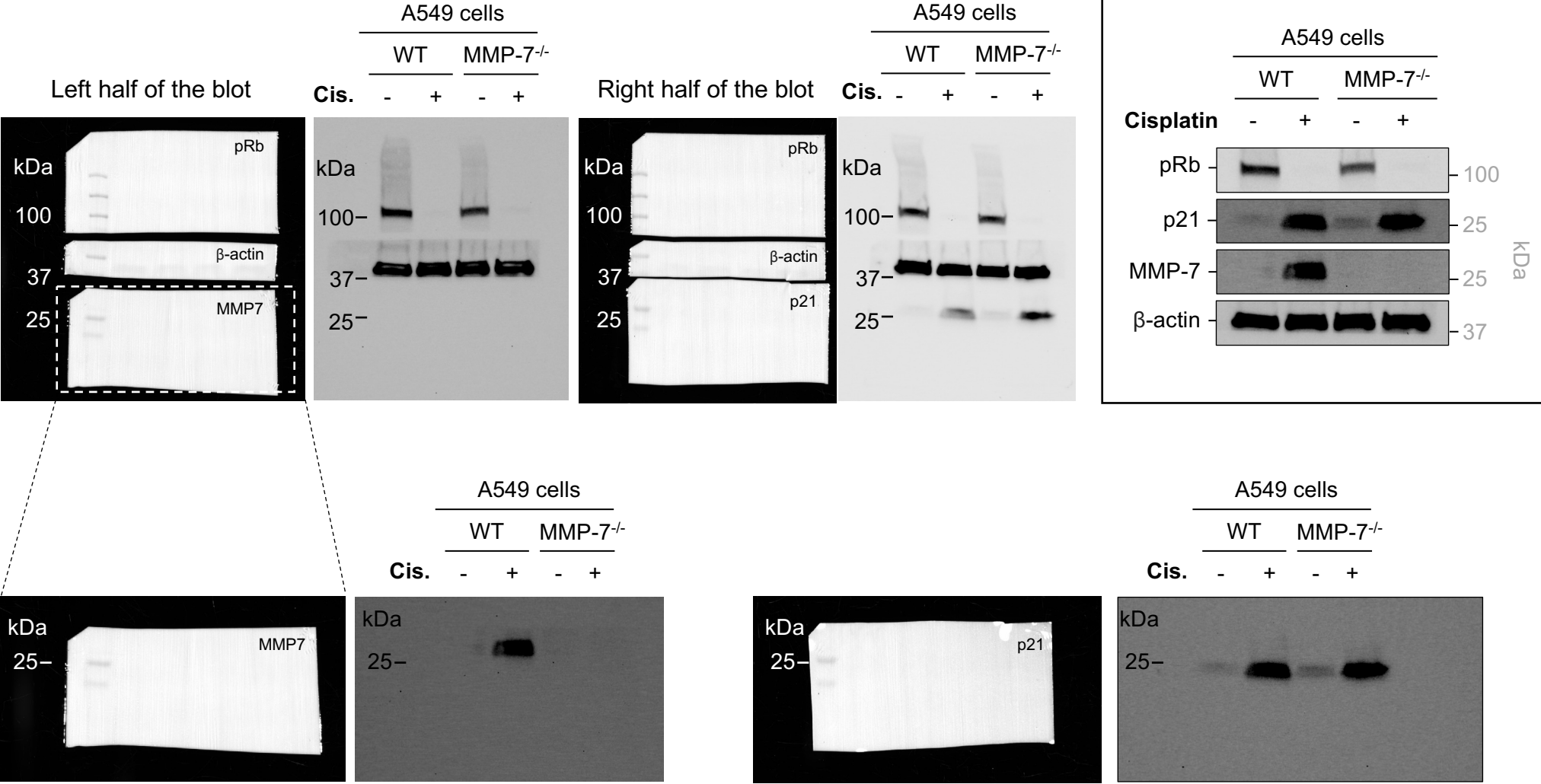

Supplement: Supplementary file 7 — PDF file containing original uncropped immunoblots for Fig. 3g. [file 43587_2026_1116_MOESM7_ESM.pdf]

Extended Data Fig. 2d

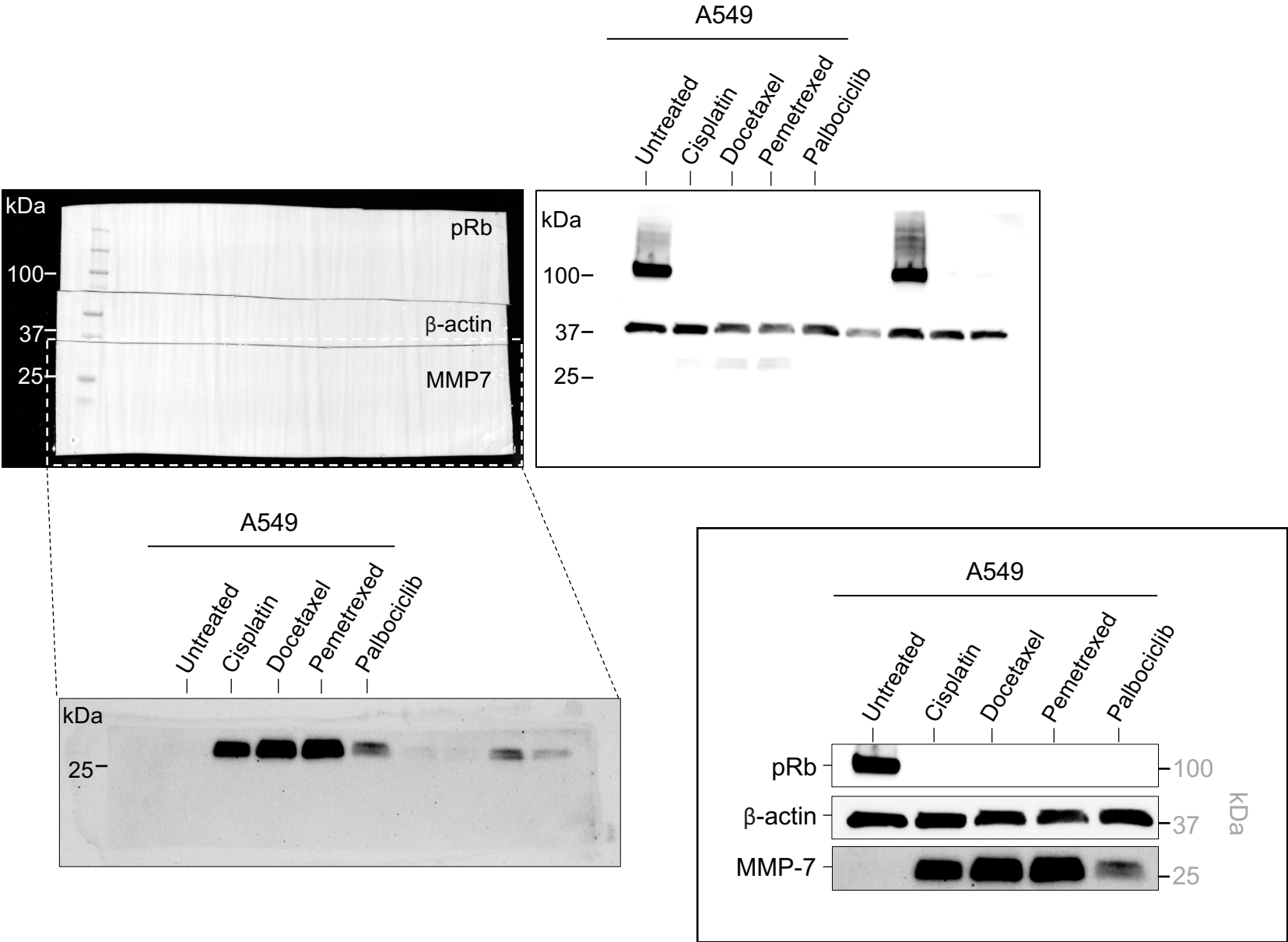

Supplement: Supplementary file 8 — PDF file containing original uncropped immunoblots for Extended Data Fig. 2d. [file 43587_2026_1116_MOESM8_ESM.pdf]
